# Contemporary Chinese newspaper discourse on translation: A mixed-methods approach to *the People’s Daily* (1949–2023)

**DOI:** 10.1371/journal.pone.0343447

**Published:** 2026-03-18

**Authors:** Xinyu Shi, Libo Huang

**Affiliations:** 1 School of Foreign Studies, Northwestern Polytechnical University, Xi’an, Shaanxi, China; 2 Foreign Language and Literature Institute, Xi’an International Studies University, Xi’an, Shaanxi, China; Tianjin University, CHINA

## Abstract

Newspaper discourse on translation forms a particular discourse that uses newspapers as a medium to discuss the products, processes, and functions of translation. It can reflect how a society perceives translation as a general phenomenon in a certain period. Adopting a mixed-methods approach to the contemporary Chinese newspaper discourse on translation, this study examines the perceived roles of translation in the *People’s Daily* between 1949 and 2023. It identifies main translation-related terms in the discourse through frequency analysis, traces the developmental trajectories of those terms through time series analysis, and investigates the discourse focuses in different periods based on corpus-assisted discourse analysis. The findings show that there are diachronic changes in the perceived roles of translation, and that such changes are closely related to the broader sociopolitical, economic, and cultural context of China. This study has some implications for expanding interdisciplinary approaches to translation studies and helps explore the interactive relationships between translation, discourse, and social changes.

## 1 Introduction

Discourse on translation, according to Cheung [1], is a term “referring to any text (works of translation included) that expresses the author’s views, ideas and theorizations on translation – on its modes of operation, its dynamics, principles and methods, and/or on the philosophy, epistemology, ontology and hermeneutics of translation”. It records how a society perceives translation phenomena in a specific historical period, shedding light on the position and function of translation in the source and/or target culture.

On that basis, newspaper discourse on translation forms a particular discourse that discusses the products, processes, and functions of translation, using newspapers as a medium. Unlike academic discourse on translation, newspaper discourse on translation exhibits a clear tendency towards popularization and practicality. Its characteristics are manifested in several aspects. First, newspaper discourse on translation targets the public as its primary readers, and thus prefers to use plain language. Second, it pays close attention to current or remarkable translation phenomena and responds rapidly to social changes. Third, using newspapers as its medium, it can reach a large audience in a short time. Fourth, covering a wide variety of fields such as politics, society, culture, art, and technology, it can reflect the role of translation in different spheres.

Newspaper discourse on translation encompasses not only some theoretical accounts of translation but also social concerns about practical issues such as the translation industry, translation technology, and translation publishing. On the one hand, it reflects how a certain society perceives translation as a general phenomenon. On the other hand, it can also shape public opinion about translation. In this sense, research into newspaper discourse on translation can reveal the constraints of socio-cultural factors on translation activities and uncover how the perceived roles of translation change over time.

Methodologically, the availability of large, continuous, and digitized newspaper archives now permits diachronic, corpus-based analysis at scale. Substantively, increasing attention to translation within contemporary communication agendas makes newspaper discourse a timely indicator of shifting priorities (see [2–6]). These conditions, together, make a systematic, periodized analysis both feasible and necessary.

But why China? China provides an analytically clear case: public discussion of translation maps onto widely recognized phases of national development (e.g., 1949–1978), and a single agenda-setting national daily—the *People’s Daily*—offers stable editorial conventions across the entire period. This configuration allows us to track how translation is framed across systemic shifts and to interpret changes in salience against a well-defined historical backdrop.

Adopting a mixed-methods approach, this paper aims to explore how translation as a general phenomenon is discussed and perceived in the contemporary Chinese newspaper discourse. It focuses on the data retrieved from the *People’s Daily* (1949–2023), one of the most representative Chinese newspapers. The specific research questions are as follows:

1) What translation-related terms are used in the *People’s Daily* discourse on translation?
2) Does the salience of the main translation-related terms change across different periods? If so, how?
3) Following the occurrences of translation-related terms in different periods, what diachronic shifts are reflected in the perceived roles of translations in China?

## 2 Research into newspaper discourse on translation

Even though newspaper discourse on translation constitutes a crucial link in the chain of translation production, circulation, and reception, it has been marginalized in translation studies. Previous studies mainly approach newspaper discourse on translation from the perspective of translation criticism, addressing such issues as translator’s (in)visibility, translation evaluation, translation quality, translation methods, as well as dissemination and reception of translated works.

Early studies of newspaper discourse on translation date back to the 1990s. Venuti [7] was one of the pioneering scholars who examined newspaper reviews of translated works. He pointed out that these reviews tended to consider fluency as the primary criterion for evaluating translated works, whereas they overlooked other possible questions like accuracy, intended audience, economic value, and literary positioning [7, p. 2]. This tendency contributed to the invisibility of translators and the marginalized status of translated works in the target culture [7, p. 2–5]. Later, based on the reviews of literary translations published in French newspapers in the 1990s, Vanderschelden [8] explored the visibility of literary translators and translated literature. The study found that, with most reviews neglecting the status of translators and translations, only a few reviews made minimal references to the translation proper, but the corresponding judgments were “vague, subjective and unsubstantiated” [8, p. 287]. In addition, the criteria for evaluating translations were simply “a set of presuppositions and subjective assumptions” which varied from one reviewer to another [8, p. 288]. Fawcett [2] investigated the translation reviews published in major British newspapers (e.g., *The Observer*) between 1992 and 1999, focusing on how translation was perceived in the British press. The findings showed that the newspaper reviews favored transparent translation and strongly disliked source-oriented modes of translation, the criticism in which lacked concrete evidence and justifiable analysis. These findings corroborated Venuti’s [7] arguments and were further investigated in follow-up studies by Gray [9], Gullin [4], and Solum [10].

Building upon these early explorations, a series of follow-up studies were carried out with a wider research scope and new methodological approaches. Bielsa [11], for example, investigated the reviews of literary translations in Spanish, American, and British newspapers from 1996 to 2012, examining the translation, dissemination, and reception of the novels by Roberto Bolaño. The study discovered that the dissemination of translated works gradually enhanced the reputation of Roberto Bolaño and his novels in cross-cultural contexts. Further, focusing on the reviews of translated works published in *The New York Times* in 1900, 1950, and 2000, Baer [3] used corpus methods to investigate the quantity, source languages, and domains of the reviewed translations in the newspaper. Based on a close reading of the reviews, the study discovered a tendency of transition across different historical periods: in 1900, the main focus was on the reproduction of the original author’s style in translated works; in 1950, the reviews focused on the role of translation in social service and intercultural communication; and in 2000, the reviews promoted readability and untranslatability. Similarly, Gray [9] constructed a corpus of translation reviews published in British and French broadsheet supplements and investigated the influence of newspaper reviews on translator’s invisibility. The study found that while most reviews still advocated for fluency and transparency in translated works, they also showed an open attitude towards diverse translation approaches, source-oriented modes of translation included. In contrast to the studies above, Gullin [4] focused on the reviews of multiple-language translations of the same novel so as to examine the reception of these translations in their respective countries. The findings showed that these reviews often equated translated works with original works, and the evaluations of translated works influenced those of the original works.

Based on the overview, it can be found that research into newspaper discourse on translation can be furthered in terms of research scope and research focus. First, existing studies mostly examine newspaper reviews of translated works but pay little attention to other article types in newspapers. In fact, newspaper discourse on translation can be presented in a wide variety of article types, such as interviews with translators, news reports on translation-related events, introductions to translation technologies, etc., with translation reviews being merely a small part of newspaper discourse on translation. Second, existing studies mainly start from the perspective of translation criticism, neglecting other potential research perspectives. By its very nature, newspaper discourse on translation is a unique discourse type that not only records public perceptions and concerns of translation, but also reflects diachronic changes in social conceptualizations of translation. Thus, newspaper discourse on translation can be approached from the perspective of how translation as a general phenomenon constructs, and is constructed by, social changes. In summary, although newspaper discourse on translation has received growing scholarly attention, there is still room for improvement in terms of expanding the research scope and exploring new research perspectives, so as to achieve a more comprehensive and in-depth understanding of newspaper discourse on translation.

## 3 Newspaper discourse on translation in Chinese contexts

Beyond Anglophone studies, some Chinese scholars treat the newspaper as an important arena where translation is publicly evaluated, normalized, and politicized. This body of work situates newspaper discourse on translation within shifting policy regimes and field dynamics, offering press-based evidence that complements descriptive translation studies. Within this line of research, the *People’s Daily* is frequently considered as a canonical site for examining how translation is positioned and problematized in state-mediated public discourse. For instance, based on the *People’s Daily* and related outlets, Li [12] reconstructed the early-PRC milieu (1950–1954) and analyzed “self-criticism of translation” with respect to its media, agents, discursive logic, and discursive effects under specific policy constraints, showing the plurality of “self-criticisms of translation”, and exploring the cultural and historical roots of “self-criticism of translation” in that period. Extending the span to 1949–1966, Geng [13, p. 137–150] investigated the *People’s Daily* discourse on translation and discovered that the central preoccupation of the translation and publishing sectors was improving translation quality to serve socialist construction. Drawing on Bourdieu’s theory of social fields, Yang [14] examined the *People’s Daily* articles (1949–1956), arguing that the translation field was strongly shaped and constrained by the field of power; when major political or economic changes occurred in the latter, their effects reverberated through the former. Furthermore, Geng and Zhou [5] applied topic modeling and corpus methods to the *People’s Daily* (1949–1966), maintaining that its discourse on translation—taken as a microcosm of the contemporary Chinese translation discourse—exhibits a deep structure shaped by “belief” and “technology”. Different from the above-mentioned studies, Zhang and Wen [6] turned to the post-2000 era and investigated important Chinese newspapers such as the *China Reading Weekly* and the *Guangming Daily*. They conducted a content analysis of Chinese newspapers (2000–2017), documenting steady growth in coverage with higher salience for translation history, translation industry, and translation theory, yet lower prominence for legal, audiovisual, and pedagogical translation. Taken together, these studies motivate our data choice and diachronic design in a policy-sensitive research tradition.

In summary, these studies have identified a recurrent focus in the discourse on translation: the peculiar link between translation and society in China. In other words, the social role of translation has always been a heatedly debated issue in the newspaper discourse. For example, in the early-PRC period, the discourse centers on improving translation quality to serve socialist construction [5,13], which can also be partly found in the self-criticisms of translation [12]. Translation is thus seen as an important power in national construction, and thus translation quality improvement is directly aligned with national development goals. In contrast, entering the 21^st^ century, translation in China is framed as an enabling capacity for international communication and cultural outreach, as suggested by the high-frequency issues in the newspaper discourse in Zhang & Wen’s [6] study. This diachronic pattern provides a substantive backdrop for our own periodized analysis and helps to interpret changes in the salience of key terms.

## 4 Data and methods

The data used in this paper was obtained from the *People’s Daily*, the official newspaper of the Central Committee of the Chinese Communist Party (CCP). As the largest newspaper in China, the *People’s Daily* provides direct information on the policies, stances, and perspectives of the CCP. It combines agenda-setting authority and nationwide circulation, with stable editorial conventions and a continuous digitized archive. These features provide a robust diachronic baseline for tracking how translation is framed across different time periods.

We constructed a monolingual corpus based on the texts collected from the *People’s Daily* database between 1949 and 2023, the titles of which included the Chinese character “译” (pinyin: *yì*, English: *translate*). The database can be accessed at https://data.people.com.cn/rmrb/20240110/1?code=2 (accessed 2024 January 10). Given that newspaper discourse on translation is public-facing and uses accessible language with few specialist theoretical terms, this retrieval helps to target article items centrally about translation. Meanwhile, restricting retrieval at the title level prioritizes topical precision: items are more likely to be centrally related to translation, whereas body-text matches often include incidental uses. After retrieval, we manually screened the list to exclude off-topic article items (e.g., “人体第二十二对染色体密码被破译” [Genetic code of the 22nd pair of human chromosomes deciphered]). We treat this precision-first strategy as a scoping choice and acknowledge that future expansions can incorporate full-text retrieval with refined positive/negative queries. The complete list of article entries in the curated corpus (n = 1,240), with dates and titles, is supplied as S1 Appendix in S1 File (original titles) and S2 Appendix in S1 File (translated titles).

All the full Chinese texts were segmented with HanLP’s pre-trained tokenizer COARSE_ELECTRA_SMALL_ZH without a user lexicon, and stop-word removal was not applied at the counting stage. Compared with other pre-trained tokenizer provided by HanLP, the COARSE_ELECTRA_SMALL_ZH tokenizer shows the best performance for our texts in terms of accuracy, segmentation granularity, and identification of out-of-vocabulary words (rare words, new words, proper nouns, etc.). According to the statistics provided by HanLP, the general performance of the COARSE_ELECTRA_SMALL_ZH tokenizer is: P: 98.34%, R: 98.38%, F1: 98.36% (https://hanlp.hankcs.com/docs/api/hanlp/pretrained/tok.html).

We then manually checked the tokenization for translation-related tokens and verified that frequent domain terms containing “译” were output as single tokens. The corpus consists of 1,240 texts in total (characters: 912,869, tokens: 489,193).

To identify diachronic shifts reflected in the perceived roles of translations in the *People’s Daily* discourse, we divided 1949–2023 into three periods anchored in widely recognized socio-historical milestones—the founding of the PRC (1949), the onset of Reform and Opening-up (1978/1979), and China’s accession to the WTO (2001). Accordingly, the three periods are as follows:

1) Period I (1949–1978): the early-PRC and pre-reform decades, with the discourse embedded in socialist nation-building and tightly coupled to policy and ideological campaigns.
2) Period II (1979–2000): the first two decades in the Reform and Opening-up phase, marked by market reforms, renewed global engagement, and a diversifying press in the early Internet era.
3) Period III (2001–2023): the post-WTO phase marked by accelerated globalization, the spread of digital news platforms, and a growing emphasis on international exchanges and cross-cultural communication.

These labels serve as descriptive scaffolds for comparison across phases; robustness checks indicate that shifting cut points by ±2 years does not change the main patterns.

The main tool used in this study is Python 3.12.5. The specific research methods are as follows:

1) *Frequency analysis*. Generate a frequency list of tokens which includes the Chinese character “译”, and select the most frequent ones as main translation-related terms for further investigation.
2) *CUSUM-based time series analysis*. Taking into consideration the different numbers of texts collected in the corpus, perform CUSUM-based time series analysis of the selected terms. CUSUM, the abbreviation of “cumulative sum control chart”, is a sequential analysis technique devised by Page [15]. It is typically used for detecting changes in value. We used an adapted version of calculation method in our study: cumulative proportion chart over period [16, p. 29], which is used to monitor the diachronic changes in cumulative proportions of certain terms or concepts. The specific calculation process includes:
  a) Calculate the frequencies of each term in each year (*f*).
  b) Count the number of texts in the corpus in each year (*n*).
  c) Divide the frequencies of each term in each year (*f*) by the number of texts in each year (*n*), so as to get normalized frequencies of the terms.
  d) On the basis of normalized frequencies, calculate and visualize cumulative usage proportions of the terms over the years from 1949 to 2023.
3) *Co-occurrence analysis*. Perform a co-occurrence analysis of the selected terms using a token-level window of ±10 tokens around each occurrence; during post-processing, manually filter out high-frequency function words and other noise to reveal which dimensions of translation the discourse foregrounds across different periods.
4) *Data-driven close reading*. Based on the quantitative statistics above, use close reading as an interpretive strategy. By focusing on the specific details of the concordances, examine the context of the terms and discern some deeper patterns and meanings present in the text.

To sum up, the study takes quantitative analysis as the starting point and conducts qualitative analysis for a deeper understanding of the *People’s Daily* discourse on translation, which integrates methods from digital humanities, corpus linguistics, and discourse analysis. By combining distant reading and close reading, we can not only identify salient features and patterns present in the discourse but also uncover the deeper meanings hidden in those features and patterns.

## 5 Results

### 5.1 Main translation-related terms in the discourse

Table 1 lists the most frequent translation-related terms in the *People’s Daily* (frequency≥100). Among these terms, “翻译” (to translate/translation) is the most prominent one, with a frequency (5370) far higher than that of any other term. Thus, we consider “翻译” as the *central* term in the discourse, with the remaining frequent tokens clustering around it as *main* terms. Given its disproportionately high frequency and marked polysemy (e.g., verb vs. noun; disciplinary register vs. policy register), as well as the broad evaluative and institutional frames it anchors, a full analysis of “翻译” is beyond the scope of the present paper and warrants a dedicated study. Here we foreground the other high-frequency terms to maintain balance across categories.

**Table 1:** The most frequent translation-related terms (frequency≥100).

No.	Term	Literal translation	Frequency
1	翻译	to translate; translating; translation	5370
2	译者	translator	678
3	译本	translation (emphasizing different versions)	620
4	译文	translation (emphasizing the textual level)	585
5	翻译家	noted translator	386
6	编译	compilation and translation	359
7	译制	film dubbing	149
8	译介	to introduce sth. via translation/translating	131
9	译名	term translation/translated term	124
10	互译	reciprocal translation	112
11	重译	re-translation	103

With regard to their frequencies in the discourse, the ten main terms in Table 1 can be further categorized into three types. High-frequency terms include “译者” (translator, 678 occurrences), “译本” (translation [emphasizing different versions], 620 occurrences), and “译文” (translation [emphasizing the textual level], 585 occurrences). Medium-frequency terms are “翻译家” (noted translator, 386 occurrences) and “编译” (compilation and translation, 359 occurrences). And relatively low-frequency terms include “译制” (film dubbing, 149 occurrences), “译介” (to introduce sth. via translation/translating, 131 occurrences), “译名” (term translation/translated terms, 124 occurrences), “互译” (reciprocal translation, 112 occurrences), and “重译” (re-translation, 103 occurrences).

Before we move on to further analysis, here are some clarifications of the terms. To begin with, both referring to the agents involved in translation, the terms “译者” and “翻译家” carry different connotations and imply different levels of expertise. “译者” is a general term for anyone who translates, regardless of their level of expertise, while “翻译家” implies a high level of professional expertise and recognition in the field of translation. In this paper, “译者” is literally rendered as “translator”, and “翻译家” as “noted translator”. Meanwhile, “译介” (to introduce sth. through translation) and “翻译” (to translate) are interchangeable in certain cases, with the former putting emphasis on the combination of introduction and translation. In what follows, we render “译介” as “to translate/translation” when appropriate.

These ten terms cover the agent of translation (“译者” and “翻译家”), translation products (“译本” and “译文”), different modes of translation (“编译”, “译制”, “译介”, and “互译”), as well as special issues in translation practice and research (e.g., “译名” and “重译”). Together, these terms can cover the basic aspects of translation as a general phenomenon in contemporary China between 1949 and 2023.

### 5.2 Cumulative proportions of main terms over time

**Fig 1:**
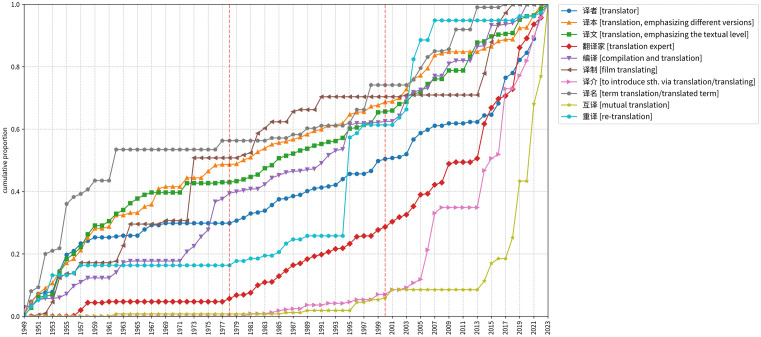
Cumulative proportions of main translation-related terms over time.

Fig 1 shows the cumulative proportions of the ten terms in the period from 1949 to 2023. The auxiliary lines in the figure demarcate different periods.

As indicated by Fig 1, the ten terms differ from each other in developmental trajectories throughout the years from 1949 to 2023. For example, the cumulative proportions of certain terms (e.g., “译名”) increase rapidly at the very outset, while those of other terms (e.g., “译介” and “互译”) remain below 0.1 until the 1990s and experience a swift growth in the 21^st^ century. Besides, during the 1960s and ’70s, the increase in cumulative proportions of most terms decelerates and even comes to a halt, yet the frequencies of a few terms (e.g., “编译” and “译制”) continue to rise in this period. Moreover, in comparison with the cumulative proportions of other terms, there is a relatively steady increase in those of “译本”, “译文”, and “译者” over the years. These distinctive traces left by the cumulative proportions of the terms demonstrate that different facets of translation have received varying degrees of attention over different time periods.

When we frame the ten terms within specific historical periods, we can identify some prominent terms in each period. Specifically, in Period I (1949–1978), “译名”, exhibiting the fastest growth in cumulative proportions (from 0 to 0.56) among all terms, is definitely the most prominent term. Meanwhile, owing to their atypical increase in cumulative proportions in the 1960s and’70s, “译制” and “编译” can also be regarded as prominent terms in this period.

In Period II (1979–2000), compared with the steady increase in cumulative proportions of most terms, the upsurge of “重译” proves to be much more conspicuous. In other words, the trends of most terms are very similar to each other, but that of “重译” stands out due to its abrupt rise between 1994 (with a cumulative proportion of 0.26) and 1997 (with a cumulative proportion of 0.61). Thus, “重译” proves to be the most prominent term in this period.

In Period III (2001–2023), almost all terms have a rapid increase in cumulative proportions, but the tendencies of the three terms “互译”, “译介”, and “翻译家” are particularly intriguing. They have quite low cumulative proportions (below 0.3) in Periods I and II, but exhibit a much steeper increase in frequency in Period III, thus being the most prominent in this period.

In addition to the above-mentioned prominent terms in each period, the high-frequency terms “译本”, “译文”, and “译者”, due to the steady increase in cumulative proportions across all three periods, can be considered as constant terms over the years.

Those prominent and constant terms in the discourse are summarized in Table 2. Based on this categorization, we will examine the discourse focuses on translation from 1949 to 2023, so as to examine what issues concerning translation are discussed in different periods. We will investigate those prominent terms (rather than all the terms) in each period to discover the most salient aspects of translation which are particularly emphasized in that very period. And then we will investigate the constant terms to discover the enduring issues over time.

**Table 2:** Prominent and constant terms in three periods.

Period	Prominent term	Constant term
Period I (1949–1978)	译名, 译制, 编译	译本, 译文, 译者
Period II (1979–2000)	重译	
Period III (2001–2023)	互译, 译介, 翻译家	

### 5.3 Discourse focuses on translation from 1949 to 2023

#### 5.3.1 Prominent terms in Period I (1949–1978).

(1) **“译名”**

Table 3 lists the top 10 words co-occurring with the term “译名” (term translation/ translated terms) from 1949 to 1978. The most notable aspect of “译名” is its standardization (统一), with the highest co-occurrence frequency of 42. Other interesting co-occurrences include “问题” (problem/issue), “词” (word), “民族” (nation), “错误” (mistake), and “读者” (reader), which may be used when discussing inconsistencies in translated terms.

**Table 3:** Top 10 words co-occurring with “译名” in Period I.

No.	Co-occurring word	Literal translation	Frequency
1	统一	standardization	42
2	问题	problem/issue	14
3	词	word	11
4	民族	nation	9
5	工作	work/job	7
6	译本	translation	7
7	著作	work/book	7
8	错误	mistake	6
9	审订	review and revise	6
10	读者	reader	5

The issue of standardizing translated terms was raised in the 1950s and remained to be a main focus thereafter. For example, Li’s [17] article entitled “On standardizing translated terms” was one of the first articles which discussed in great detail the issue of term translation. Li pointed out that standardizing translated terms was a technical and complicated issue. After listing many inconsistencies of proper name translation in books, newspapers, and magazines, Li expressed his hope that, on the one hand, the press (such as Xinhua News Agency and Xinhua Bookstore) should use standardized translated terms in its publications, and, on the other hand, the relevant authoritative government department should play a role in this area.

Similar problems also existed in the translation of film titles:

Example 1

我们最近收到报社转来的读者意见，对某些翻译片译名提出批评。经研究后，我们认为这些意见基本上是正确的，有作公开答复的必要。

国营厂翻译片的工作开始于一九四九年，在此以前，苏联影片已在各地上映，每片有一种以上的译名。我们开始翻译影片后，即统一审订译名。

我们统一审订译名的工作作得很不好。据初步检查，三年来经我们审订的翻译片译名中，有不少种是不妥的。…… [18]

We have recently received reader comments forwarded by the People’s Daily press criticizing the translation of certain film titles. After investigation, we acknowledge that these opinions are basically correct and that it is necessary to make a public reply.

The state-run film studios began translating foreign films in 1949. Prior to that, Soviet films had already been screened in many places, with each film having more than one translated title. When we started translating films, we also began to standardize, review and revise the translation of film titles.

We did a poor job in this aspect. According to preliminary inspections, many of the translations that have been reviewed and revised by us in the past three years are inappropriate....

This is a public reply from the Arts Committee of the Central Film Administration in 1952. It accepted the criticism from readers, reflected on the existence of inconsistently translated titles for films, and admitted that many revised translations of film titles are inappropriate.

Furthermore, in 1955, Bi and Xu [19] co-authored the article entitled “On standardization of Chinese in translation work”, relating standardization of translated terms with standardization of the Chinese language. After pointing out the major role played by translation in standardizing and enriching the Chinese language, Bi and Xu [19] analyzed the potential impact of inconsistent translated terms from two aspects. First, inconsistencies in term translation might directly influence readers’ understanding of the translation. Second, such inconsistencies might cause ambiguity and redundancy in the Chinese language.

Later, two articles [20,21] were published in the 1960s, specifically discussing the word “民族” (nation) from the perspective of term translation. One of the articles analyzed the use of relevant terms in original Marxist classics, examined their translations in Chinese, and ended with the conclusion that standardizing the use of “民族” (nation) in Chinese translations was vital for readers in their study of Marxist classics:

Example 2

由此可见，为了使读者在学习马克思主义经典著作（尤其是斯大林的著作）的民族问题理论时不再发生误解，“民族”一词的译名统一问题是必须解决的。 [20]

It can thus be seen that, in order to prevent misunderstandings in the study of the theories on nationalities question in Marxist classics (especially Stalin’s works), the issue of standardizing the translation of the word “民族 (nation) must be resolved.

The other article [21] introduced a forum on the use of “民族” (nation) and “部族” (tribe) in translations. The attendants of the forum had an in-depth discussion concerning the use of “民族” (nation) and “部族” (tribe) in translated Marxist classics, and finally selected “民族” (nation) as a standardized translation, in the hope of standardizing translated terms and eliminating inconsistencies in translated Marxist classics.

Along this line, we can find that the discourse in Period I (1949–1978) pays special attention to the impact of inconsistent translated terms. Existing extensively in books, newspapers, magazines, films, and even translated Marxist classics, inconsistent translated terms can not only cause difficulties for readers to understand the translations [17,20], but also result in ambiguity and redundancy in the Chinese language [19].

(2) **“译制”**

Table 4 lists the top 10 words co-occurring with the term “译制” (film dubbing) in Period I. It is not surprising that high-frequency co-occurrences of “译制” are “影片” (film/movie), “电影” (film/movie), “故事片” (narrative film), and “配音” (dubbing). What interest us most are actually the words “苏联” (Soviet Union), “东北电影制片厂” (Northeastern Film Studio), “语言” (language), and “少数民族” (ethnic minorities).

**Table 4:** Top 10 words co-occurring with “译制” in Period I.

No.	Co-occurring word	literal translation	FREQUENCY
1	影片	film/movie	49
2	工作	work/job	18
3	苏联	Soviet Union	18
4	电影	film/movie	17
5	配音	dubbing	12
6	东北电影制片厂	Northeastern Film Studio	10
7	语言	language	8
8	少数民族	ethnic minorities	8
9	我国	our country	7
10	故事片	narrative film	5

After the founding of the PRC, a large number of Soviet films were translated for and introduced to the Chinese people. The following is an excerpt of a report from the Translation Department of the Northeast, Shanghai and Beijing Films of the Film Bureau of the Central Ministry of Culture:

Example 3

一九五○年，我们国营东北、上海、北京三制片厂除完成原计划翻译四十部苏联故事片，与三十六本苏联科学教育短片外，并为配合抗美援朝保家卫国运动，超额译制了《秘密使节》、《阴谋》与《钢铁是怎样炼成的》三部故事片，苏联科学教育短片也超额完成了六本。 [22]

In 1950, our state-run film studios in Northeast China, Shanghai, and Beijing finished the originally planned translation of 40 Soviet narrative films and 36 Soviet science-educational shorts. Additionally, in support of the Campaign of Resisting U.S. Aggression and Aiding Korea to Defend Our Homeland, we also translated three extra narrative films (“Secret Envoy”, “Conspiracy” and “How Steel Is Tempered”) as well as another six science-educational shorts from the Soviet Union.

This report summarized the work progress in film translation. It clearly pointed out that translating Soviet films was in line with the Movement of Resisting U.S. and Aiding North Korea. That is, translating Soviet films was of great significance to nation building. We can find more examples:

Example 4

东北电影制片厂五年来已翻译了九十六部苏联故事影片和四十部动画片、科学教育片，对中苏文化交流起了重要的作用。东北电影制片厂译制的反映苏联人民高尚品质和人类最美好的生活的苏联影片在中国放映后，受到我国广大观众的欢迎。 [23]

Over the past five years, the Northeast Film Studio has translated 96 Soviet narrative films as well as 40 animated and science-educational films, which have played an important role in cultural exchanges between China and the Soviet Union. The Soviet films translated by the Northeast Film Studio, which reflect the noble qualities of the Soviet people and the most beautiful aspects of human life, have been warmly received by audiences across China upon their screening.

Example 5

中国电影发行公司负责人今天对新华社记者说，中国今年将翻译苏联艺术片四十六部……除故事片外，中国还将译制苏联新闻纪录片、科学教育片等近一百部。 [24]

The head of the China Film Distribution Company told Xinhua News Agency reporters today that China will translate 46 Soviet art films this year... In addition to narrative films, China will also translate nearly 100 Soviet newsreels, science-educational films, and so on.

Based on Examples 4–5, we can see that translating Soviet films seemed like an important and serious task at that period. And the types of translated films, mainly narrative films and science-educational films, not only served nation building as mentioned before, but also promoted the cultural exchanges between China and the Soviet Union. Besides, the Northeast Film Studio made a great contribution to translating Soviet films.

Apart from this, much effort was exerted to translate Chinese films into other languages, especially the languages of ethnic minorities in China:

Example 6

新疆各族人民都迫切希望能够看到愈来愈多的用他们的语言译制的祖国影片。 [25]

The people of all ethnic groups in Xinjiang eagerly hope to watch more and more domestic films translated into their languages.

Example 7

故事片《李双双》已经译制成蒙古语片，不久将在内蒙古草原上放映。 [26]

The narrative film “Li Shuang-shuang” has been translated into Mongolian and will soon be screened in Inner Mongolia.

Example 8

我国电影《白毛女》和《党的女儿》最近在北京电影制片厂用马里邦巴拉语译制成功。 [27]

China’s films ‘The White-haired Girl’ and ‘Daughters of the Party’ have recently been successfully translated into the Bambara language at the Beijing Film Studio.

The excerpts above show that the work of translating films into ethnic minority languages started in the early 1950s and continued in the 1960s. Afterwards, it gained increasing attention in the 1970s:

Example 9

广西壮族自治区各级电影部门积极用当地少数民族语言，配音译制电影。目前已用壮语、侗语等配音译制了十五部影片。这些影片在各少数民族地区放映后，受到热烈欢迎。

……

广西壮族自治区电影部门在用少数民族语言配音译制影片的同时，还积极发展少数民族地区的电影放映事业，使各少数民族人民群众能比较及时地看到本民族语言配音译制的电影。 [28]

Film agencies at all levels in Guangxi Zhuang Autonomous Region are actively dubbing films in local minority languages. So far, 15 films have been dubbed in Zhuang and Dong languages. These films have been warmly welcomed upon screening in minority areas.

......

Film agencies in Guangxi Zhuang Autonomous Region, while dubbing films into minority languages, are also actively developing film screening undertakings in minority areas, ensuring that the ethnic minority masses can watch the films dubbed into their native languages in time.

Based on the analysis above, we can find that film translation proved to be a significant undertaking in the period from 1949 to 1978. The primary task was translating Soviet films into China, and the secondary task was translating films into ethnic minority languages. Correspondingly, there existed two purposes in film translation: the first one was to promote socialist construction by translating films from the Soviet Union, and the second purpose was to strengthen national unity by translating films into the languages of ethnic minorities.

(3) **“编译”**

Table 5 lists the top 10 words co-occurring with the term “编译” (compilation and translation) in Period I. We can see that the most notable co-occurrence of “编译” is “出版” (publishing), with the highest co-occurrence frequency of 53. Other interesting co-occurrences include “科学” (science), “教科书” (textbook), “苏联” (Soviet Union), “教材” (textbook), and “藏文” (Tibetan language).

**Table 5:** Top 10 words co-occurring with “编译” in Period I.

No.	co-occurring word	literal translation	frequency
1	出版	publishing	53
2	工作	work/job	24
3	科学	science	12
4	教科书	textbook	9
5	苏联	Soviet Union	9
6	工作者	worker/staff	9
7	人员	personnel/staff	8
8	图书	book	8
9	教材	textbook	6
10	藏文	Tibetan language	6

The reason why “出版” (publishing) and “科学” (science) co-occur frequently with “编译” is attributed to the establishment and activities of a special group, i.e., “中国科学院编译出版委员会” (Committee on Compilation, Translation, and Publication of the Chinese Academy of Sciences). This committee was established on May 19th, 1956, which was an important event in translation circles. Its principles and tasks included:

Example 10

决定科学出版工作的方针和任务；审查、批准科学出版工作的计划并且领导和监督计划的执行；组织近代学术论著丛刊的编纂工作以及组织编订各学科科学名词和解决科学著作编译出版工作中的其他重大问题。 [29]

Determining the principles and tasks for scientific publishing; reviewing and approving plans for scientific publishing, and directing and supervising their implementation; organizing the compilation and translation of modern academic anthology series, determining scientific terminology in all disciplines, and resolving other major issues in the compilation, translation, and publication of scientific works.

Adhering to the above principles, this committee played a key role in compiling, translating and publishing scientific works in the 1950s:

Example 11

大家在发言中提出，为了贯彻“百家争鸣”方针，今后编译出版工作，必须充分反映中国的和世界各国科学研究成果，以及学术上的争论。 [30]

In their speeches, the attendants proposed that, in order to implement the policy of ‘Contention of all schools of thought’, future work on compilation, translation, and publication must fully reflect the scientific research achievements and academic debates in China and other countries all over the world.

This excerpt above is about a plenary meeting held by the committee on September 5th, 1956, concerning how to serve scientific research through the compilation, translation, and publication of scientific works. The attendants stressed the importance of the scientific research results and academic debates at home and abroad.

Apart from scientific works, Soviet textbooks and plays were also in the scope of compilation, translation and publication during the 1950s:

Example 12

……旧教科书的观点、材料与方法比较陈旧，新编译的苏联中学教科书的观点、材料与方法都比较进步，和东北中学生的程度也相接近。 [31]

The views, materials and methods in the old textbooks are relatively outdated, while those of the newly compiled and translated Soviet secondary school textbooks are more progressive and are closer to the level of students in Northeastern secondary schools.

Example 13

中国戏剧出版社决定有计划地编译出版苏联著名作家剧作集丛刊，作为对十月革命节的献礼。 [32]

The China Theatre Publishing House has decided to systematically compile, translate, and publish a series of plays by famous Soviet writers as a tribute to the October Revolution.

Examples 12–13 above show that compiling, translating and publishing Soviet works was an effective and practical way to update the textbooks used by secondary students and promote bilateral relations between China and the Soviet Union.

Additionally, book compilation, translation, and publication in minority languages (such as Tibetan and Mongolian) has remained a focal point in the 1950s, and gained increased attention in the 1970s. Such work was improved gradually in terms of the development of translation institutions, staff recruitment, as well as the capability of compiling and translating in minority languages:

Example 14

委员会还为拉萨小学的学生编译了算术、藏文等课本十多种，为拉萨市人民医院、邮电局编译了“藏语业务会话”…… [33]

The Committee has also compiled and translated over 10 textbooks such as arithmetic and the Tibetan language for students in primary schools of Lhasa, as well as ‘Practical Conversations in Tibetan’ for the Lhasa People’s Hospital and Telecommunications Bureau...

Example 15

几年来，八省、自治区为落实党的民族政策，加强蒙古文图书编译出版工作，普遍建立、健全了编译机构，增加了编译人员，同时提高了蒙古文印刷能力。 [34]

Over the past few years, in order to implement the Party’s ethnic policies and improve the work on Mongolian book compilation, translation, and publishing, eight provinces and autonomous regions have generally established and improved their translation institutions, increased the number of translators, and improved the printing capabilities in Mongolian.

According to Examples 14–15, it seems that the work on compilation and translation mainly served three purposes in Period I (1949–1978). Firstly, it helped to promote educational development. Second, it contributed to international friendship with the Soviet Union. Third, it could strengthen unity between different ethnic groups. All the three purposes pointed to the socialist construction between 1949 and 1978, the early years of the People’s Republic of China.

#### 5.3.2 Prominent term in Period II (1979–2000).

“重译” (retranslation) is the most prominent term in the period from 1979 to 2000. Table 6 lists the top 10 words co-occurring with the term “重译” (retranslation) in Period II. The most noticeable co-occurrence of “重译” is “名著” (classic), with the highest co-occurrence frequency of 45. It is natural that retranslation co-occur frequently with *classics*, since the reputation of original works is often one of the primary factors which determine the success of their translations. The other interesting high-frequency co-occurrence is “热” (fever), with a co-occurrence frequency of 16. The remaining words in Table 6 are low-frequency co-occurrences of the term “重译”.

**Table 6:** Top 10 words co-occurring with “重译” in Period II.

No.	Co-occurring word	Literal translation	Frequency
1	名著	classic	45
2	热	fever	16
3	问题	problem/issue	9
4	出版	publishing	8
5	文化	culture	7
6	世界	world	5
7	文学	literature/literary	5
8	图书	book	4
9	市场	market	4
10	现象	phenomenon	4

The period from 1979 to 2000 witnessed the publication of many retranslations, such as those of *Red Star Over China*, *Le Père Goriot*, *La Dame aux Camélias*, *Notre-Dame de Paris*, *Le Rouge et le Noir*, *Die Leiden des jungen Werthers*, and Marxist classics, etc., covering a wide range of literature. This growing trend of retranslating famous works (especially world-famous literary classics) was gradually noticed and dispersedly debated in the public in the 1980s.

Wang [35] was a pioneer who elaborated upon the issue of retranslation. He considered the retranslation boom as a prominent cultural phenomenon which reflected the cultural needs of Chinese readers in that special period. He goes on by relating the retranslation boom to relevant publishing departments:

Example 16

但是，近年来，面对读者对世界文学名著需求的上升之势，不少出版部门一方面受出版利益的吸引，另一方面出于版权问题的考虑，便开始在名著重译上做文章，重译本由此大量出现。 [35]

However, in recent years, faced with the rising demand for world-famous literary classics, many publishing departments, attracted by the interests on the one hand and out of copyright considerations on the other hand, have started to take advantage of retranslating classics, leading to the emergence of a large number of retranslations.

Wang partially attributed the emergence of numerous retranslations to certain publishing departments which were attracted and driven by the interests. Then, he made an in-depth analysis of the problems in retranslation and pointed out three main problems with retranslation: (a) the publishers’ seeking of profits, (b) the hasty organization of translator teams and the lack of academic ability, and (c) repeated publications. These factors turned retranslation into an abnormal phenomenon in Chinese culture. Publishers, according to Wang, are primarily responsible for the retranslation boom. By providing some suggestions at the end of the article, Wang [35] called on the government to enhance the management of the publishing mechanism.

Wang’s article [35] was the most representative one that systematically and retrospectively reflected on the growing trend of retranslating classics (especially world-famous literary works) and on the irresponsible publication of retranslations. It served as a warning against low-quality retranslations in the period from 1979 to 2000.

#### 5.3.3 Prominent terms in Period III (2001–2023).

(1) **“互译”**

Table 7 lists the top 10 words co-occurring with the term “互译” (reciprocal translation) in Period III. The most noticeable co-occurrence of “互译” is “出版” (publishing), with the highest co-occurrence frequency of 85. Other interesting co-occurrences are “经典” (classics), “签署” (sign), “备忘录” (memorandum), “两国” (two countries), “亚洲” (Asia), “中国” (China), and “国家” (nation).

**Table 7:** Top 10 words co-occurring with “互译” in Period III.

No.	Co-occurring word	Literal translation	Frequency
1	出版	publishing	85
2	经典	classics	68
3	著作	work/book	58
4	签署	sign	46
5	备忘录	memorandum	45
6	两国	two countries	29
7	亚洲	Asia	26
8	中国	China	22
9	国家	nation	22
10	作品	work/book	20

Relating the term “互译” with the high-frequency co-occurrences in Table 7, we may come to a preliminary conclusion that those words jointly help to create such a context where a series of memoranda of cooperation on reciprocal translation and publication were signed between China and other countries (especially Asian countries). There is an early example in the discourse:

Example 17

2013年5月，中国国家新闻出版广电总局与俄罗斯出版与大众传媒署在莫斯科联合签署了《“中俄经典与现当代文学作品互译出版项目”合作备忘录》。双方商定，6年内相互翻译并出版对方国家不少于50种经典作品，或双方共出版不少于100种图书。 [36]

In May 2013, China’s State Administration of Press, Publication, Radio, Film and Television and Russia’s Federal Agency for Press and Mass Media jointly signed the ‘Memorandum of Cooperation on the Reciprocal Translation and Publication of Chinese-Russian Classic and Contemporary Literary Works’ in Moscow. The two sides agreed to translate and publish no less than 50 classic works from each other’s country, or co-publish no less than 100 books within 6 years.

This China-Russia memorandum of cooperation in 2013 represented the beginning of reciprocal translation and publication between China and other countries. Further, in 2019, the Asian Classics Translation Program was initiated, in accordance with the important proposal put forward by General Secretary Xi Jinping at the Conference on Dialogue of Asian Civilizations:

Example 18

亚洲经典著作互译计划为各国间的交流互鉴提供了一个平台。截至目前，我国已与18个亚洲国家签署图书互译出版合作协议，互译出版了70多种精品图书。 [37]

The Asian Classics Translation Program has provided a platform for exchanges and mutual learning among Asian countries. To date, China has signed cooperation agreements on reciprocal translation and publication with 18 Asian countries, and over 70 quality books have been mutually translated and published.

The Asian Classics Translation Program aimed to promote mutual cultural translation and civilizational exchanges among the 47 Asian countries. Following the initiation of this program, a large number of memoranda of cooperation were signed between China and Asian countries. To date, China has signed memoranda of cooperation on the reciprocal translation and publication of classical works with Singapore, Pakistan, South Korea, Iran, Laos, Armenia, Kyrgyzstan, Azerbaijan, Mongolia, Yemen, Thailand, Jordan, Nepal, Saudi Arabia, Turkey, Vietnam, North Korea, and Qatar:

Example 19

2021年1月5日，中巴双方宣布签署《中华人民共和国国家新闻出版署与巴基斯坦伊斯兰共和国国家遗产和文化署关于经典著作互译出版的备忘录》…… [38]

On January 5, 2021, China and Pakistan announced the signing of the ‘Memorandum Between the National Press and Publication Administration of the People’s Republic of China and the National Heritage and Culture Division of the Islamic Republic of Pakistan on the Reciprocal Translation and Publication of Classical Works’….

Example 20

2021年1月18日，中韩双方以交换文本的方式签署了《中华人民共和国国家新闻出版署与大韩民国文化体育观光部关于经典著作互译出版的备忘录》…… [39]

On January 18, 2021, China and South Korea signed the ‘Memorandum Between the National Press and Publication Administration of the People’s Republic of China and the Ministry of Culture, Sports and Tourism of the Republic of Korea on the Reciprocal Translation and Publication of Classical Works’ through an exchange of documents.…

This series of memoranda on reciprocal translation and publication of classics show that China is paying increasing attention to the role played by translation in bridging an inter-cultural dialogue between China and other Asian countries on the one hand, and in enhancing China’s relationship with surrounding countries on the other hand.

(2) **“译介”**

Table 8 lists the top 10 words co-occurring with the term “译介” (to introduce sth. via translation/translating) in Period III. The most noticeable co-occurrences of “译介” are “文学” (literature) (with the highest co-occurrence frequency of 65), “文化” (culture) (with a co-occurrence frequency of 54), and “中国” (China) (with a co-occurrence frequency of 53). Other interesting co-occurrences are “中华” (China/Chinese), “当代” (contemporary), “传播” (spread), “工程” (project), and “世界” (world).

**Table 8:** Top 10 words co-occurring with “译介” in Period III.

No.	Co-occurring word	Literal translation	Frequency
1	文学	literature	65
2	文化	culture	54
3	中国	China	53
4	作品	work/book	39
5	中华	China/Chinese	30
6	当代	contemporary	17
7	传播	spread	12
8	工程	project	11
9	工作	work/job	11
10	世界	world	10

The frequent co-occurrences of “文学” and “译介” can be mainly attributed to the fact that, entering the 21^st^ century, a growing number of people have noticed unbalanced literary exchanges between China and other countries:

Example 21

据中国作家协会副主席金炳华介绍，当前中外文学作品译介存在 “信息单行道” 格局。大量外国文学作品能很快地被译介到国内来，而中国文学译介出去的就少得多。 [40]

According to Jin Binghua, Vice President of China Writers Association, there is currently a ‘one-way street’ pattern in the translation and introduction of literary works between China and other countries. A large number of foreign literary works can be quickly translated and introduced to China, but far fewer Chinese literary works are translated and introduced abroad.

Such a “one-way street” pattern in literary exchanges implied that it was easier for the Chinese audience to get access to translated foreign literature, but foreign readers might have little or no access to translated Chinese literature. Even though some Chinese literature has been translated into foreign countries, there are still some problems:

Example 22

过去虽也有一些书籍被译成外文出版，但零零散散，不成系统。国外也有对中华文化著作的译介，但品种屈指可数，有的纯属猎奇，错误很多；有的为了达到某种政治目的，故意篡改、歪曲，影响十分恶劣。 [41]

In the past, although some books have been translated into foreign languages and published, they were sporadic and lacked systematicity. While there have been translations and introductions of Chinese cultural works abroad, the number is exceedingly limited. Some of these works are purely driven by curiosity and contain many errors; others have been deliberately altered or distorted for certain political purposes, resulting in highly detrimental effects.

According to Example 22, Chinese literature was not widely received in the system of world literature, and thus the position of Chinese literature on the world stage was unsatisfactory. For one thing, the number of translated Chinese literary works in foreign countries was limited; for another, the existing translations were disappointing in terms of their translation quality and literary influence, and sometimes even deviated considerably from the original Chinese works. In order to improve this situation, China launched a project entitled “中国当代文学百部精品译介工程” (100 Contemporary Chinese Literary Masterpieces Translation Project):

Example 23

中国作家协会将启动 “中国当代文学百部精品译介工程”，推动更多的当代文学作品走出国门。 [42]

China Writers Association is set to launch the ‘100 Contemporary Chinese Literary Masterpieces Translation Project’ to promote more contemporary literary works to go global.

Example 24

旨在推动中国文学走向世界、树立中华民族崭新形象的 “中国当代文学百部精品译介工程”，目前已确定首批译介作品，将分别介绍到俄罗斯、波兰、古巴等3个欧美国家。 [40]

The ‘100 Contemporary Chinese Literary Masterpieces Translation Project’, aimed at promoting Chinese literature globally and establishing a new image for the Chinese nation, has already selected the first batch of works for translation. These works will be introduced to European and American countries including Russia, Poland, and Cuba.

According to Examples 23–24, the “100 Contemporary Chinese Literary Masterpieces Translation Project” aimed to introduce Chinese literature to foreign countries and thus promote the Chinese literary position in the world, so as to improve the overseas promotion of contemporary Chinese literature.

In the 2010s, there also emerged an increasing demand for cultural translation. As pointed out by Jiang [43], there was, first and foremost, an asymmetry between China and other countries in the translation and dissemination of cultural information (not limited to literature). Additionally, cultural translation in the past was narrowed down to and even equated with the translation of voluminous literary classics, the outcomes of which were not encouraging due to a quite limited audience.

However, as advocated by Jiang [43] and Xu [44], the focuses of cultural translation could be quite varied:

Example 25

从传播内容来看，除了传统出版、影视机构的大部头作品之外，更多外国人对中国文化的兴趣往往来自碎片化的信息、文章乃至故事、评论、视频和图片，他们想“采购”的，往往不是“成套设备”，而是“零售商品”，这部分工作其实也不可小视。……凡此种种看似不完整、不系统、跨界、短平快、消费性极强的文化信息需求，恰恰很少得到中国对外文化译介扶持体系的关注和支持，其结果，就是导致中国文化的国际形象常常过于死板紧张，缺少灵活变通。 [43]

In terms of dissemination content, aside from voluminous works by traditional publishing and film agencies, foreign interest in Chinese culture often stems from fragmented information, articles, stories, reviews, videos, and images. What they want to ‘purchase’ is often not ‘complete sets of equipment’ but ‘retail goods.’ This part of the work should not be underestimated. Various forms of demand, on the seemingly incomplete, unsystematic, cross-border, short-term, and highly consumable cultural information, rarely gain the attention and support from China’s system of outward cultural translation and introduction. As a result, China’s international cultural image often appears too rigid and tense, lacking flexibility and adaptability.

Example 26

推动中华文化走出去，做好中华文化译介工作至关重要。这里强调的中华文化译介，不仅包括文学作品的译介，也包括中华文化典籍的译介，还包括能够展示当代中国鲜活思想的哲学社会科学研究成果的译介。 [44]

It is crucial to promote Chinese culture abroad and do a good job in translating Chinese culture. The Chinese culture, emphasized here, includes not only literary works but also Chinese cultural classics as well as philosophical and social science research outcomes that can showcase contemporary China’s vibrant thoughts.

Cultural translation covered the translation of various text types, ranging from full-length books (such as literary works and cultural classics) to fragmented information (such as reviews, images, and videos). This extension from literary translation to cultural translation is significant for introducing Chinese culture (not merely Chinese literature) to the world. In addition, He Mingxing, a scholar of international news and communication, put forward the practice of multilingual cultural translation:

Example 27

……将多语种对外译介的重要性上升到国家对外文化传播的战略高度，尽早考虑人才队伍的年轻化、国际化，推动多语种人才和文化传播的战略性规划。 [45]

… [we need to] elevate the importance of multilingual translation to the strategic height of national cultural dissemination, consider the early development of a young, international talent team, and promote the strategic plans concerning multilingual talents and cultural dissemination.

More often than not, cultural translation means to translate and introduce Chinese culture to English-speaking countries, due to the global influence of the English language. Considering growing cultural exchanges between China and other countries all over the world (non-English speaking countries included), multilingual cultural translation is a step forward, on the basis of cultural translation, to promote Chinese culture to the world at large.

The shift from literary translation to cultural translation (and even to multilingual cultural translation) in the period from 2001 to 2023 indicates that China has been continuously adapting to the needs of new situations and making adjustments accordingly. Thus, translation proves to occupy an important position in improving literary communication as well as cultural exchanges between China and other countries.

(3) **“翻译家”**

Table 9 lists the top 10 words co-occurring with the term “翻译家” (noted translator) in Period III. The most noticeable co-occurrences of “翻译家” are “文学” (literature), with the highest co-occurrence frequency of 83. Other interesting co-occurrences are “中国” (China), “作家” (writer), “文化” (culture), “青年” (youth), “出版家” (publisher), and “学者” (scholar).

**Table 9:** Top 10 words co-occurring with “翻译家” in Period III.

No.	Co-occurring word	Literal translation	Frequency
1	文学	literature	83
2	中国	China	42
3	作家	writer	42
4	作品	work/book	34
5	著名	famous	28
6	文化	culture	22
7	青年	youth/young	19
8	资深	experienced	16
9	出版家	publisher	16
10	学者	scholar	15

The frequent co-occurrences of “翻译家” and “文学” were mainly used to recognize the contribution of noted translators in China’s literary translation:

Example 28

中国文学作为世界文学重要的组成部分，是一个客观的存在。然而，中国文学若不经过汉学家、翻译家的努力，那么它作为世界文学的构成部分就很难实现。 [46]

Chinese literature, as an important part of world literature, is an objective existence. However, without the efforts of sinologists and noted translators, it would be difficult to realize the role of Chinese literature as an integral part of world literature.

Example 29

2日，在柏林举行的第二十届世界翻译大会会员代表大会上，中国文学翻译家许渊冲荣获国际翻译界最高奖项之一——国际翻译家联盟（国际译联）2014 “北极光” 杰出文学翻译奖，成为该奖项1999年设立以来首位获此殊荣的亚洲翻译家。 [47]

On the 2nd [of August], at the 20th World Congress of the International Federation of Translators held in Berlin, noted Chinese literary translator Xu Yuanchong was awarded one of the highest honors in the international translation community – the 2014 International Federation of Translators (FIT) “Aurora Borealis” Prize for Outstanding Translation of Fiction Literature. He is the first Asian translator to receive this honor since the establishment of the award in 1999.

Noted translators played a vital role in establishing Chinese literature as a significant component of world literature. As China’s Nobel-winning novelist Mo Yan [46] noted, Chinese literature, though an objective existence, could only realize its full potential through the dedicated efforts of sinologists and noted translators. A prime example of such contributions is Xu Yuanchong, who was awarded the 2014 “Aurora Borealis” Prize for Outstanding Translation of Fiction Literature by the International Federation of Translators [47]. This recognition underscored the crucial role that noted translators played in bridging Chinese literature with the global literary community.

Given this special role of noted translators, there also emerged the discussion of the standards or requirements for a “good” literary translator. Mira Ahmed, an Egyptian sinologist and literary translator, pointed out that a “good” literary translator must accurately reflect the original author’s style and diction in translation, convey the cultural background and thinking characteristics of the original work, and have strong expressive abilities in both languages [48,49]. Those standards would ensure that readers cannot easily distinguish between the original text and the translation.

In order to better translate and introduce Chinese literature to the world, the relationship between noted translators and writers was also mentioned:

Example 30

翻译是技术问题，也是学术问题，更是情感问题。要把中国文学准确地、传神地翻译成外文，应该不断加深翻译家与中国作家之间的了解，也要加深翻译家同行间的了解。 [46]

Translation is a technical issue, an academic issue, and an emotional issue. To accurately translate Chinese literature into foreign languages, we should constantly deepen the mutual understanding between noted translators and Chinese writers, as well as the mutual understanding among noted translators.

According to Mo Yan [46], the mutual understanding between noted translators and Chinese writers should be deepened. The enhanced mutual understanding could allow noted translators to better grasp writers’ intentions and style, leading to more faithful and evocative translations. Additionally, it helped foster collaboration and trust, which could result in higher-quality translations that resonated more deeply with international audiences.

It is worth noting that, faced with the limited number of noted translators, the public also discussed the cultivation of young translators:

Example 31

目前，文学翻译主要依靠两支队伍，一支是老一代翻译家，另一支是中青年译者。令人遗憾的是，老一代翻译家队伍的人数正日渐减少，优秀的中青年译者也是供不应求。 [50]

Currently, literary translation mainly relies on two groups: one group is the older generation of noted translators, and the other group consists of young and middle-aged translators. Unfortunately, the number of the older generation of noted translators is gradually decreasing, and there is a shortage of outstanding young and middle-aged translators.

Example 32

目前从事中译外工作的中坚力量主要是老一辈翻译家，优秀中青年译者严重不足，翻译队伍“断层”令人担忧。 [51]

The current backbone of those engaged in outward translation work is mainly the older generation of translators, with a severe shortage of outstanding young and middle-aged translators. This ‘gap’ in the translation team is worrying.

The limited (and also decreasing) number of noted translators led the public to reflect on how to develop young translators for literary translation. According to the excerpts above, the field of literary translation relied heavily on noted translators. Unfortunately, the number of noted translators was dwindling, and there was a significant shortage of outstanding young and middle-aged translators, resulting in a gap in the translation team. Therefore, the cultivation of young translators was crucial to ensure the continuity and quality of Chinese literary translation.

The analysis above shows that the significant contributions made by noted translators in the field of Chinese literary translation have received continued attention in Period III (2001–2023). On this basis, discussions have emerged concerning the standards for good translators, the relationship between noted translators and writers, and the cultivation of young translators.

#### 5.3.4 Constant terms across different periods.

The three terms “译本”, “译文”, and “译者”, which cover the product and agent of translation, can be taken as constant terms from 1949 to 2023, since there is a relatively steady increase in their cumulative proportions. In essence, these three terms are the basic terms in translation studies. And they are unavoidable when we describe and explain translation phenomena. Thus, not surprisingly, these terms prove to be the consistent focus across different periods.

Due to space limitations, the following is a generalization about the use of “译本”, “译文”, and “译者” from 1949 to 2023 based on co-occurrence results, paying special attention to differences among the periods.

Table 10 lists the top 10 words co-occurring with the three terms in different periods. The most frequent co-occurrence words are highlighted in grey.

**Table 10:** Top 10 words co-occurring with “译本”, “译文”, and “译者” in three periods.

keyword	Period I (1949–1978)	Period II (1979–2000)	Period III (2001–2023)
译本	出版 (publication, 91), 著作 (works, 33), 毛泽东选集 (*Selected Works of Mao Tse-tung*, 32), 新 (new, 27), 中文 (Chinese, 22), 中国 (China, 20), 列宁 (Lenin, 19), 新华社 (Xinhua News Agency, 19), 斯大林 (Stalin, 18), 发行 (issue, 16)	出版 (publication, 46), 新 (new, 30), 我国 (our country, 14), 文学 (literature, 13), 英文 (English, 13), 译者 (translator, 12), 全 (complete, 12), 名著 (classic, 11), 中国 (China, 11), 中文 (Chinese, 11)	出版 (publication, 27), 新 (new, 24), 读者 (reader, 20), 文学 (literature, 20), 中国 (China, 20), 全 (complete, 19), 译者 (translator, 19), 不同 (different, 17), 原著 (original work, 14), 作品 (works, 13)
译文	读者 (reader, 20), 译者 (translator, 19), 原文 (source text, 17), 出版 (publication, 15), 错误 (mistake, 14), 提高 (improve, 14), 中国 (China, 13), 文学 (literature, 13), 正确 (correct, 13), 原作 (original work, 12)	原文 (source text, 25), 质量 (quality, 19), 出版 (publication, 17), 准确 (accurate, 13), 全集 (complete works, 12), 列宁 (Lenin, 11), 恩格斯 (Engels, 10), 文献 (literature, 10), 马克思 (Marx, 9), 著作 (works, 9)	原文 (source text, 24), 质量 (quality, 19), 修订 (revision, 18), 准确 (accurate, 17), 穆旦 (Mu Dan, 16), 著作 (works, 13), 读者 (reader, 13), 译者 (translator, 12), 马克思 (Marx, 11), 恩格斯 (Engels, 10)
译者	出版 (publication, 22), 出版社 (publisher, 20), 译文 (translated text, 19), 出版者 (publisher, 16), 工作 (work, 15), 新 (new, 15), 译本 (translation, 12), 读者 (reader, 11), 态度 (attitude, 10), 熟悉 (familiar, 10)	出版 (publication, 17), 作品 (works, 13), 工作 (work, 13), 文学 (literature, 12), 译本 (translation, 12), 新 (new, 11), 读者 (reader, 10), 中国 (China, 8), 译文 (translation, 7), 作者 (author, 7), 问题 (problem, 7)	文学 (literature, 44), 作品 (works, 36), 中国 (China, 32), 读者 (reader, 25), 作者 (author, 22), 问题 (problem, 20), 要求 (requirement, 19), 译本 (translation, 19), 语言 (language, 18), 文化 (culture, 18)

As indicated by Table 10, the three terms “译本”, “译文”, and “译者” are inextricably interwoven in the *People’s Daily* discourse on translation. For one thing, the three terms share many high-frequency co-occurrence words such as “出版” (publication), “文学” (literature), “读者” (reader), and “中国” (China). For another, the three terms even serve as co-occurrence words of each other in certain cases.

However, those constant terms are discussed from different perspectives in different periods. Specifically, with regard to “译本”, although the primary focus on translations remains to be *publication* (“出版”) over the three periods, Period I mainly concentrates on the translations of the works by political leaders such as “毛泽东” (Mao Zedong), “列宁” (Lenin), and “斯大林” (Stalin), whereas Periods II and III pay more attention to the translations of *literary works* (“文学”). Besides, more agents involved in translation activities, such as the *translator* (“译者”) and *reader* (“读者”), are highlighted in Periods II and III.

As to “译文”, the primary focus on translated texts shifts from the *reader* (“读者”) in Period I to *original text* (“原文”) in Periods II and III. Particularly, after 1979, the *quality* (“质量”) of translated texts (especially *accuracy* [“准确”]) attracts more attention. Additionally, the genres under discussion shift from *literary works* (“文学”) in Period I to Marxist works in Periods II and III. That means, the translation quality of Marxist works gains its importance after 1979 in the discourse. Furthermore, Period III also witnesses more discussions about *revision* (“修订”), the *reader* (“读者”) and the *translator* (“译者”) of translated texts.

With respect to “译者”, the primary focus on translators changes from *publication* (“出版”) in Periods I and II to *literature* (“文学”) in Period III. Moreover, in the close context of “译者”, the three co-occurrence words *China* (“中国”), *reader* (“读者”), and *author* (“作者”) prove to be particularly emphasized after 2001. Thus, it seems that, in recent years, the relationships between the translator, China, the reader, and the author have gained increasing interest in the public.

Based on those observations, it is safe to say that, even though translations and translators have been the constant focus, we can still discover some shifts over the years. First, after 1979, literary translation and translated literature has grown into an important focus in the discourse. Second, translation quality (more specifically, accuracy) has been particularly highlighted in the period between 1979 and 2023. Third, translators’ relationships with literary works, China, the reader, and the author are becoming increasingly close from Period I to Period III.

## 6 Discussion

The ten main terms identified in the *People’s Daily* discourse on translation are closely connected with the basic aspects of translation, including the agent of translation, translation products, different modes of translation, as well as certain special issues in translation practice and research. Those ten terms vary in their developmental trajectories as they have distinctive cumulative proportions over the years. Thus, some terms tend to be more prominent in a specific period, while others have been constantly discussed throughout the past 75 years.

Now, if we reflect on the findings in light of China’s broader sociopolitical, economic, and cultural context, we can discover some changes in the perceived roles of translation across different periods.

In Period I (1949–1978), i.e., the early years of China’s socialist construction, the Chinese public pays special attention to the political function of translation. This period witnesses the heated discussion of three major issues: (i) standardizing translated terms, (ii) film translation, and (iii) compilation and translation. All the three issues are closely related to socialist construction after the establishment of the PRC. For example, standardizing translated terms, especially those in the translations of Marxist classics, helps lay the foundation for readers to correctly understand the thoughts of Marx, Engels, Lenin, and Stalin (see [17,19]). Similarly, translating Soviet films, together with compiling and translating Soviet books, allows China to align itself closely with the Soviet Union and, further, strengthens the political and ideological bond between the two countries (particularly during the period from 1949 to the early 1960s). Additionally, based on the educational and propagandistic value of translations, translating films and books into minority languages contributes to enhanced unity between different ethnic groups in China.

In Period II (1979–2000), following the implementation of the Reform and Opening-up policy, the Chinese public restores the multi-perspectival discussion about translation, and particularly reflects upon translation’s role in repositioning world literature in China. The issue of retranslation is more prominent in this period. Due to historical reasons, a large number of world classics are translated and retranslated to compensate for the slow cultural development in the 1960s and ’70s. And retranslating world classics becomes a noticeable trend in the publishing industry in the 1990s, leading to some serious problems such as repeated publications and poor-quality translations. Such a retranslation boom is critically discussed by the Chinese public from different perspectives. For example, Wang [35], one of the most representative articles, regards it as a response to the needs of Chinese readers for world classics, points out that the proliferation of hastily produced, low-quality retranslations is driven by commercial interests, and calls for improved quality of retranslations. In this sense, it can be said that translation is expected to play dual roles in, on the one hand, meeting the needs of Chinese readers for world classics, and on the other hand, ensuring the quality of world classics introduced into China. That is, faced with the audience’s enthusiasm for world classics, (re)translations should still have a high quality.

In Period III (2001–2023), after China’s accession to the WTO, the Chinese public is more concerned with the role of translation in promoting literary exchanges and cultural communication between China and other countries in the world. Three issues prove to be rising topics in the discourse: (i) reciprocal translation between China and other countries, (ii) introducing foreign literature and culture into China via translation, and (iii) the function of noted translators in introducing Chinese literature to the world. It can be found that all the three issues are related with strengthening the relationship between China and the world at large in terms of literature and culture. For one thing, with the advancements in technology, communication, and transportation, the 21^st^ century is characterized by increasing interconnectedness across national borders. For another, emerging as a major global power, China has been wielding significant influence in various spheres, including economics, politics, technology, and culture. Against this background, translation has to respond to the literary and cultural opportunities and challenges facing China in the 21^st^ century, and thus its role in mediating China’s literary identity and facilitating cross-cultural understanding becomes increasingly crucial. In this sense, translation needs to foster appreciation for Chinese literature and culture in the world and enrich the Chinese cultural landscape with diverse voices from other countries.

The above-discussed roles of translation in different periods are also partly reflected in the changing contexts of “译本”, “译文” and “译者”, which have been analyzed in the previous Subsection 4.3.4. For example, the close contexts of “译本”, “译文” and “译者” after 1979 highlight literary translation/translated literature as well as translation quality, which roughly corresponds to the perceived role of translation in Period II in terms of meeting the needs of Chinese readers for world classics and ensuring the quality of world classics introduced into China.

## 7 Conclusion and implications

This study combines frequency analysis, time series analysis, and corpus-assisted discourse analysis, adopting a mixed-methods approach to the contemporary Chinese newspaper discourse on translation (1949–2023). It examines how the Chinese public perceives translation as a general phenomenon over the years. The study discovers diachronic changes in the perceived roles of translation in different periods, and those identified changes are closely related to China’s broader sociopolitical, economic, and cultural context.

The study has some implications for expanding interdisciplinary approaches to translation studies. By integrating the methods from digital humanities, corpus linguistics and discourse analysis, the study takes quantitative analysis as the starting point, and conducts qualitative analysis for a deeper understanding of the *People’s Daily* discourse on translation. This combination of distant reading and close reading is helpful not only in identifying significant features and patterns present in the discourse, but also in uncovering the deeper meanings hidden in those features and patterns. Thus, the study promotes interdisciplinary dialogues between translation studies, digital humanities and discourse analysis, and helps expand interdisciplinary approaches to translation studies.

Meanwhile, the study also contributes to the exploration of the interactive relationships between translation, discourse, and social changes. Translation, as a communicative act, plays a crucial part in shaping public discourse and facilitating social changes, while at the same time, it is also influenced by the prevailing discourse and the broader social context in which it takes place. Newspaper discourse on translation, in this sense, is an important discourse form that reflects such dynamic and complex relationships.

Focusing on the *People’s Daily* discourse on translation, this paper takes a tentative step towards framing the interactive relationships within the specific context of China and investigates the diachronic changes in the perceived roles of translation between 1949 and 2023. Given the polysemy and salience of “翻译”, we have examined this focal term in a separate, dedicated analysis.

Beyond “译” lexemes, future work can incorporate conceptual term sets (e.g., “信”, “达”, and “雅”) as secondary queries to surface evaluative discussions that may not contain the morpheme “译.” Follow-up research can pair these with negative filters to limit topical drift and replicate the pipeline across additional outlets to assess robustness.

Additionally, in future research, the interactive relationships between translation, discourse, and social changes can be framed in many different contexts and can be explored from more perspectives. Follow-up studies can triangulate these findings with additional newspapers (e.g., the *Guangming Daily*) and extend retrieval beyond titles to full-text queries with expanded positive term sets and exclusion rules for off-topic hits. Furthermore, comparative analyses across outlets can assess the stability of co-occurrence structures and periodization.

## Supporting information

S1 File S1 Appendix. Complete list of article entries. S2 Appendix. Complete list of article entries (English version).
(ZIP)
