# Supplementary material for: Contemporary Chinese newspaper discourse on translation: A mixed-methods approach to the People’s Daily (1949–2023)
Source: PLoS One. 2026 Mar 18;21(3):e0343447. doi: 10.1371/journal.pone.0343447 (PMC12998856; doi:10.1371/journal.pone.0343447)
Supplement: S1 File — S1 Appendix. Complete list of article entries. S2 Appendix. Complete list of article entries (English version). (ZIP) [file pone.0343447.s001.zip › Supporting Information/S1 Appendix. Complete list of article entries.pdf]

| No. | Date | Title |
|---|---|---|
| 1 | 1949.06.11 | 扫除殖民地习气　编译中文工业教材　清华工学院组委员会研究讨论刘仙洲教授所提草案 |
| 2 | 1949.07.24 | 波总统贝鲁特　在波兰解放日的演辞　译自波兰通讯社七月二十一日电讯稿 |
| 3 | 1949.07.26 | 美帝国主义在台湾　节译自六月一日“新时代”杂志 |
| 4 | 1949.08.04 | 列宁斯大林著作即在波出版译本 |
| 5 | 1949.08.10 | 纽约和哈卜根小镇　摘译自“美国真貌” |
| 6 | 1949.08.15 | 我们亲爱的莫斯科　译自一九四九年二月五日苏联文学报 |
| 7 | 1949.10.24 | 捷共编译中国人民政协文献　毛泽东主席特撰序文　这些文献将帮助欧洲人民认识新中国 |
| 8 | 1949.11.14 | 苏联－－强大的工业与集体农业国家　摘译自苏联共产主义青年团真理报 |
| 9 | 1949.11.15 | 苏联－－强大的工业与集体农业国家－－摘译自苏联共产主义青年团真理报－－ |
| 10 | 1949.11.27 | 新译俄文鲁迅选集 |
| 11 | 1949.12.03 | 革大四百余干部　业余学习俄文　要求九个月后有普通读译能力 |
| 12 | 1950.01.08 | 毛泽东主席名著深受缅人民欢迎　“论人民民主专政”缅译本畅行 |
| 13 | 1950.01.10 | 全国体育总会筹委会举行常务会议　设秘书处和起草、研究、宣传编译、全运设计四个委员会 |
| 14 | 1950.02.08 | 译《莫斯科性格》的一点感想 |
| 15 | 1950.03.15 | 谈西文人名地名的翻译 |
| 16 | 1950.03.26 | 用严肃的态度对待翻译工作 |
| 17 | 1950.03.26 | 潮锋出版社出版的所谓“全部新译本”《钢铁是怎样炼成的》 |
| 18 | 1950.04.09 | 认真改进翻译工作 |
| 19 | 1950.04.26 | 德人民热情纪念列宁诞辰　列宁全集德译本即将发行 |
| 20 | 1950.04.28 | 关于“苏联”的译名　“苏联”、“苏俄”、“俄国”不容混用 |
| 21 | 1950.05.03 | 马・恩・列・斯著作中译本目录 |
| 22 | 1950.05.03 | 关于译名统一 |
| 23 | 1950.05.21 | 译诗两首 |
| 24 | 1950.05.23 | 我政协共同纲领俄译本在苏出版　莫斯科举行中国诗歌晚会 |
| 25 | 1950.05.31 | 关于张译《费尔巴哈与德国古典哲学的终结》的批 |

| | | |
|---|---|---|
| | | 评与自我批评　王若水给本刊的两封信 |
| 26 | 1950.05.31 | 对三联书店版《贝多芬传》译序和附录的几点意见 |
| 27 | 1950.06.16 | 加强翻译工作者的联系　“翻译通报”即将出版 |
| 28 | 1950.07.08 | 翻译通报第一期出版 |
| 29 | 1950.07.12 | 要多出几本好的翻译书！ |
| 30 | 1950.08.14 | 《翻译通报》第二期出版 |
| 31 | 1950.09.06 | 翻译界应坚持批评 |
| 32 | 1950.11.22 | 翻译通报第五期　备各地科学工作者填表函索 |
| 33 | 1950.12.23 | 翻译通报一卷六期备翻译工作者填表索取 |
| 34 | 1950.12.23 | 燃料工业部编译　电气技术新书出版　新华、三联两书店代售 |
| 35 | 1951.01.14 | 苏出版“实践论”译本 |
| 36 | 1951.03.04 | 电影局三制片厂翻译片工作者报告工作 |
| 37 | 1951.03.28 | 世界和平理事会　关于缔结和平公约的宣言订正译文 |
| 38 | 1951.03.31 | 在侵略朝鲜的战争中　麦克阿瑟公然使用蒋匪人员在台湾征用翻译人员和空军军官赴朝 |
| 39 | 1951.04.11 | 论翻译理论的建设 |
| 40 | 1951.05.27 | 评张宗炳译《原理与方法》修订本 |
| 41 | 1951.06.07 | 真理报发表彼特罗夫专文　介绍鲁迅文集的俄译本　指出鲁迅作品贯穿着对人民的热爱和对压迫者的憎恨 |
| 42 | 1951.06.14 | 试用新编译的自然科学教科书的体验 |
| 43 | 1951.06.15 | 毛主席的《实践论》　泰国《文讯》刊出泰文译文 |
| 44 | 1951.06.25 | 《战斗的中国》的两个译本 |
| 45 | 1951.07.22 | 缅甸人民十分关注新中国　《实践论》等名著译成缅文　仰光正筹组中缅友好协会 |
| 46 | 1951.07.27 | 翻译书籍的引用文字应注明中译本出处 |
| 47 | 1951.08.06 | 不纯洁不健康的译文举例 |
| 48 | 1951.08.12 | 格利佛游记的《译后散记》 |
| 49 | 1951.08.18 | 著作或译作都要注明著成的年月 |
| 50 | 1951.10.10 | 把最好的工作成果献给伟大的祖国　把译好的“米丘林选集”献给毛主席 |
| 51 | 1951.10.25 | 大家都来积极参加马克思、恩格斯、列宁、斯大林著作中文译本的审查 |
| 52 | 1951.11.10 | 毛泽东选集　第一批已全部发行完毕　现正译为少数民族文字 |
| 53 | 1951.11.11 | 放映内容较深的翻译影片时应印发说明书 |
| 54 | 1951.11.26 | 被俘美军士兵贝尔的信和录音广播的译文 |
| 55 | 1951.11.29 | 出版总署召开第一届全国翻译工作会议 |

| | | |
|---|---|---|
| 56 | 1951.12.14 | 毛主席的“实践论”已译成朝鲜文出版 |
| 57 | 1952.01.19 | 粗枝大叶官僚主义作风为害严重　中央贸易部发生两大浪费事件　防疫药“三吨”误写“三百吨”积压资金四十多亿元“米茶砖”误译为“黑茶砖”使国家损失二十二亿元 |
| 58 | 1952.03.03 | 鲁迅译的果戈理的巨著“死魂灵”重印出版 |
| 59 | 1952.03.12 | 关于翻译片名庸俗化的答复 |
| 60 | 1952.04.11 | 新疆人民出版社大量发行民族文字书刊　“毛泽东选集”的翻译出版工作正积极进行中 |
| 61 | 1952.04.28 | 语文读本的编译者应重视读本内容 |
| 62 | 1952.05.14 | 外国史地教科书中人名和地名翻译应该统一 |
| 63 | 1952.05.17 | “毛泽东选集”第一卷苏联出版俄文译本 |
| 64 | 1952.05.20 | 苏联人民踊跃购买“毛泽东选集”俄译本 |
| 65 | 1952.05.28 | 序史剧“屈原”的俄文译本 |
| 66 | 1952.05.30 | 人民教育出版社编审部　接受统一教科书译名的建议 |
| 67 | 1952.06.12 | 苏联将出版俄译“通鉴纲目” |
| 68 | 1952.06.22 | 毛泽东选集在日本受到热烈欢迎　捷克斯洛伐克出版“中国共产党的三十年”译本 |
| 69 | 1952.07.03 | 中国影片经理公司总公司　注意关于翻译片的宣传解释工作 |
| 70 | 1952.08.18 | “学习译丛”一九五二年第七号内容介绍 |
| 71 | 1952.09.09 | 介绍“学习译丛” |
| 72 | 1952.10.31 | 斯大林同志伟大新著在各国大量印行　“苏联社会主义经济问题”中译本即将出版 |
| 73 | 1952.11.07 | 斯大林的演说和马林科夫的报告　中译本定今日出版 |
| 74 | 1952.11.19 | 苏联书刊受到我国广大人民热烈欢迎　三年来全国出版的苏联书籍中译本共约三千一百多种 |
| 75 | 1952.12.23 | 新华社关于苏联共产党简称译名的更正 |
| 76 | 1952.12.26 | 中央高等教育部领导高等学校及有关机关　进行苏联高等学校教材翻译工作 |
| 77 | 1953.01.05 | 我们已能翻译俄文教材了 |
| 78 | 1953.04.01 | 纠正介绍苏联先进农业科学书籍在翻译出版中的浪费 |
| 79 | 1953.04.19 | 新疆人民出版社用三种民族文字翻译毛泽东选集第二卷 |
| 80 | 1953.04.22 | “资本论”的中译本重版即将发行 |
| 81 | 1953.05.10 | 朝鲜出版毛泽东选集第一卷上册译本 |
| 82 | 1953.05.17 | 介绍最近出版的斯大林著作单行中译本 |
| 83 | 1953.05.27 | 对翻译出版苏联农业科学书籍工作中发生浪费的检 |

| | | |
|---|---|---|
| | | 讨 |
| 84 | 1953.06.08 | 朝鲜停战谈判行政性会议继续举行　双方参谋人员和翻译人员举行行政性会议 |
| 85 | 1953.08.04 | 外国语学校改进教学工作培养翻译人材 |
| 86 | 1953.08.26 | 关于加强用少数民族语翻译影片工作的问题 |
| 87 | 1953.09.05 | 苏联出版“毛泽东选集”俄译本第三卷 |
| 88 | 1953.09.12 | 中立国监察委员会波兰委员写信给克拉克　抗议美方劫持中立国视察小组波兰译员 |
| 89 | 1953.10.16 | 巴黎“人道报”记者贝却敌报道　美国观察员和蒋匪译员公然阻挠解释工作 |
| 90 | 1953.11.05 | 解释帐篷中美方“代表”和“翻译”的丑态 |
| 91 | 1954.03.19 | 中共中央马克思恩格斯列宁斯大林著作编译局举办马克思列宁主义经典著作展览会 |
| 92 | 1954.04.05 | 我国今年将译制四十多部苏联等国的影片 |
| 93 | 1954.04.16 | 科学教育电影制片厂　今年将摄制和翻译许多部科学教育影片 |
| 94 | 1954.04.22 | 五部藏语翻译影片在藏族地区开始发行 |
| 95 | 1954.05.06 | 苏联书籍在我国翻译出版数量飞速增长 |
| 96 | 1954.05.17 | “神曲”和“莎士比亚戏剧集”　中译本重印出版 |
| 97 | 1954.05.19 | “斯大林全集”第九卷中译本出版 |
| 98 | 1954.06.13 | 达尔文著作“物种起源”　中译本第一分册已出版 |
| 99 | 1954.06.17 | 宪法草案译成蒙、藏、维、哈和朝鲜等族文字 |
| 100 | 1954.06.27 | 苏联国家文艺书籍出版局东方部　翻译和出版中国文学作品 |
| 101 | 1954.08.16 | 加强对文学翻译工作的领导 |
| 102 | 1954.08.16 | “不求甚解”的翻译态度 |
| 103 | 1954.08.29 | 中国作家协会召开全国文学翻译工作会议 |
| 104 | 1954.08.29 | 为发展文学翻译事业和提高翻译质量而奋斗－一九五四年八月十九日在全国文学翻译工作会议上的报告摘要 |
| 105 | 1954.08.29 | 谈文学翻译工作 |
| 106 | 1954.09.08 | 关于“为进一步提高苏联文学而斗争”的译文的检讨 |
| 107 | 1954.11.09 | 东北电影制片厂译制了大量苏联影片 |
| 108 | 1954.12.12 | 政治经济学教科书译本将在明年四月左右出版 |
| 109 | 1955.01.29 | 我文化代表团为仰光华侨演出　缅甸翻译协会宴请我文化代表团 |
| 110 | 1955.02.28 | 马克思恩格斯列宁斯大林著作　今年将陆续出版三十二种中文译本 |
| 111 | 1955.03.24 | 许多重要文件已译成藏文 |

| | | |
|---|---|---|
| 112 | 1955.04.12 | “波立特选集”等中译本出版 |
| 113 | 1955.04.23 | 亚洲国家文学作品中译本增多 |
| 114 | 1955.07.06 | 科学技术书籍的翻译质量必须提高 |
| 115 | 1955.07.06 | 对于译名的两点意见 |
| 116 | 1955.08.30 | 翻译出版更多的外国优秀著作 |
| 117 | 1955.10.19 | 介绍“印度的艺术与建设” 印度纪录电影制片厂出品中央新闻纪录电影制片厂译制 |
| 118 | 1955.11.07 | 中译苏联文艺书籍大量出版 |
| 119 | 1955.12.02 | 读“巴甫洛夫选集”中译本 |
| 120 | 1955.12.06 | 关于翻译工作中的汉语规范化问题 |
| 121 | 1955.12.09 | 看纪录影片“日本和平的歌声” 日本纪录片制作协议会、日本音乐中央总会摄制 长春电影制片厂译制 |
| 122 | 1955.12.25 | “斯大林全集”第十二卷中译本出版 |
| 123 | 1955.12.28 | “列宁全集”第一卷中译本出版 |
| 124 | 1956.01.12 | 马克思、恩格斯、列宁、斯大林全集 1960 年前将全部译成中文 |
| 125 | 1956.01.14 | “马克思恩格斯文选”第二卷中译本开始发行 |
| 126 | 1956.03.09 | 苏共代表大会文件 中译本出版 |
| 127 | 1956.05.21 | 中国科学院编译出版委员会成立 |
| 128 | 1956.07.10 | 诗经选译 |
| 129 | 1956.07.13 | 中医研究院编译中医经典著作 |
| 130 | 1956.07.17 | “资本论”朝鲜文译本出版 |
| 131 | 1956.07.19 | 北京翻译工作者举行“翻译标准”座谈会 |
| 132 | 1956.08.22 | 纳赛尔著的“革命哲学”一书译成中文 |
| 133 | 1956.09.01 | 日本译注出版“满文老档” |
| 134 | 1956.09.02 | “狄德罗哲学选集” 陈修斋、王太庆、江天骥译三联书店出版陈修斋 |
| 135 | 1956.09.02 | 拉·梅特里的“人是机器” 顾寿观译 王太庆校三联书店出版 |
| 136 | 1956.09.05 | 合理地使用翻译人材 |
| 137 | 1956.09.06 | 尼赫鲁著作“印度的发现”中文译本出版 |
| 138 | 1956.09.07 | 科学院编译出版委员会 讨论编译出版工作怎样贯彻“百家争鸣” |
| 139 | 1956.09.09 | 玄妙的译文 |
| 140 | 1956.09.16 | 诗经今译 |
| 141 | 1956.10.05 | 法国学者译注拉施特“集史”中印度史和法兰克史部分 |
| 142 | 1956.10.07 | 波兰人民喜爱中国作品 翻译五十种书籍发行达百 |

| | | |
|---|---|---|
| | | 万册 |
| 143 | 1956.10.12 | 劳动部关于积压俄文翻译人员问题的答复 |
| 144 | 1956.10.21 | 译名问题 |
| 145 | 1956.10.23 | 苏联科学院讨论生产自动化　会议集中讨论了使用电子计算机的问题，表演了自动化翻译的技术，还谈到了一种新的知识部门——信息论 |
| 146 | 1956.10.26 | 译名要尽量做到统一 |
| 147 | 1956.10.28 | 关于阎译 |
| 148 | 1956.11.02 | 范缜“神灭论”发今译 |
| 149 | 1956.11.16 | 读“古诗今译” |
| 150 | 1956.11.23 | 介绍“个性心理学”　苏联 A·G·费多罗娃等著　贾明等译　科学出版社出版 |
| 151 | 1956.11.28 | 如何改进和发展翻译工作 |
| 152 | 1956.11.28 | 欢迎古代哲学论文的今译 |
| 153 | 1956.12.03 | 诗经今译 |
| 154 | 1956.12.15 | 一个不用翻译的国际会议　第四十一次国际世界语大会印象记 |
| 155 | 1956.12.28 | 读“关于阎译” |
| 156 | 1957.01.03 | “原子医学译丛”创刊 |
| 157 | 1957.01.11 | 翻译人员和外语学校学生的希望和要求 |
| 158 | 1957.01.25 | 阿富汗民间诗歌选译 |
| 159 | 1957.02.14 | 我国将译制一百多部苏联影片 |
| 160 | 1957.02.16 | 吠陀诗句的古代汉译 |
| 161 | 1957.02.17 | 苏联将出版我国古今名著　“诗经”“红楼梦”等首次译成俄文 |
| 162 | 1957.02.26 | 苏联大力发展同各国的文化联系　今年将有五千名文化工作者到国外访问　目前在出版翻译书籍方面占世界第一位 |
| 163 | 1957.02.27 | 科学出版社扩大出书范围　外国科学译著的出版开始增多 |
| 164 | 1957.02.28 | 应该严肃地对待经典著作的翻译——谈“俄国资本主义的发展”曹译本的一些问题 |
| 165 | 1957.03.26 | 瘫痪青年江幼农坚持自学十二年　写成并翻译近百万字的书籍 |
| 166 | 1957.04.02 | 改进翻译工作 |
| 167 | 1957.04.13 | 重新安排了翻译人员的工作 |
| 168 | 1957.04.23 | 战友——一个俄语翻译员的日记片断 |
| 169 | 1957.04.24 | 关于歌德作品初期的中译 |
| 170 | 1957.05.25 | “资本论”译者王亚南入党 |

| | | |
|---|---|---|
| 171 | 1957.06.01 | 翻译和出版 |
| 172 | 1957.06.17 | 卫莱－－一位有成绩的中国文学翻译家 |
| 173 | 1957.08.06 | 人民卫生出版社编译“谢切诺夫选集” |
| 174 | 1957.08.16 | 哲学、社会科学许多名著将陆续译成中文出版 |
| 175 | 1957.08.20 | “文艺理论译丛”第一期出版 |
| 176 | 1957.09.02 | 1957 年世界文化名人著作中译本陆续出版 |
| 177 | 1957.09.21 | “人类不平等的起源和基础”中译本出版 |
| 178 | 1957.09.26 | 出版苏联文学名著中译本 |
| 179 | 1957.10.15 | 纪念十月革命四十周年　编译出版苏联作家剧作集 |
| 180 | 1957.10.15 | 文艺报、译文出专号 |
| 181 | 1957.10.15 | 湘西编译苗文书籍 |
| 182 | 1957.10.19 | 大量译制苏联影片 |
| 183 | 1957.11.21 | 三辑“戏剧理论译文集” |
| 184 | 1957.12.21 | 外贸部应统一翻译出版进口设备说明书 |
| 185 | 1958.01.26 | 苏联出版“诗经”俄译本 |
| 186 | 1958.02.04 | “西方名著提要”中译本出版 |
| 187 | 1958.02.27 | 介绍“文艺理论译丛” |
| 188 | 1958.03.06 | 译风也要改 |
| 189 | 1958.03.26 | 哲学研究所鼓足干劲　修订计划加速编译进度 |
| 190 | 1958.04.14 | 谈谈翻译 |
| 191 | 1958.05.29 | 向翻译家和评论家进一言 |
| 192 | 1958.11.27 | 赫鲁晓夫关于国民经济控制数字的报告提纲　人民出版社即将翻译出版 |
| 193 | 1958.11.30 | 赫鲁晓夫的报告提纲译成中文出版 |
| 194 | 1958.12.25 | 从“按劳分配，按需分配”的正译想起的 |
| 195 | 1959.01.24 | 从“译文”到“世界文学” |
| 196 | 1959.02.01 | 赫鲁晓夫在苏共代表大会上的报告　中文译本即将在我全国各地印行 |
| 197 | 1959.02.18 | 经典著作要介绍，译文要评论 |
| 198 | 1959.03.07 | 简评“五四时期期刊介绍”　中共中央马克思、恩格斯列宁、斯大林著作编译局研究室编　人民出版社出版 |
| 199 | 1959.03.25 | “堂吉呵德”全译本出版 |
| 200 | 1959.03.28 | 对于“资产阶级法权”一语译法的意见 |
| 201 | 1959.04.01 | 漫谈译名 |
| 202 | 1959.04.22 | 译成八十八种语文　发行了三亿多册　列宁的著作向亿万人传布了革命真理 |
| 203 | 1959.06.03 | 科学书籍和翻译书籍的出版一九五六年苏联均占第一 |
| 204 | 1959.06.08 | 需要在翻译园地上修枝剪叶 |

| | | |
|---|---|---|
| 205 | 1959.06.17 | 国外学术论文摘要　“学术译丛”第六期今日出版，下面是其中三篇译文的摘要 |
| 206 | 1959.07.11 | 英国出版“楚辞”英译本 |
| 207 | 1959.07.18 | 计算技术研究所等单位正积极研究机器翻译问题 |
| 208 | 1959.07.18 | 苏联对机器翻译的研究取得很大进展 |
| 209 | 1959.09.05 | 英国物理学家贝尔纳的巨著　“历史上的科学”中译本即出版 |
| 210 | 1960.03.27 | 苏联观众喜爱中国影片　“万水千山”等影片已译成俄语 |
| 211 | 1960.12.22 | 电子计算机翻译科学文献　用氧钻采矿加速掘进速度　焦炭干熄装置生产效率巨大　用仪器帮助游泳者改进技术 |
| 212 | 1961.01.23 | 袁枚的《黄生借书说》译意 |
| 213 | 1961.02.10 | 中译《巴黎公社会议记录》即将出版 |
| 214 | 1961.02.11 | 《邹忌讽齐王纳谏》译意 |
| 215 | 1961.05.04 | 林则徐译书 |
| 216 | 1961.05.20 | 从译意风中听到的－－在亚非作家会议东京紧急会议上 |
| 217 | 1961.05.28 | 马亚文字之谜初步揭开　苏科学家用电子计算机译解出部分文字 |
| 218 | 1961.07.06 | 商务印书馆等有关出版社　翻译出版外国哲学社会科学著作 |
| 219 | 1961.07.18 | 从严译《原富》按语看严复的经济思想 |
| 220 | 1961.10.18 | 柬埔寨出版“雪花飘飘”译本 |
| 221 | 1961.11.10 | 翻译鲁迅的诗 |
| 222 | 1961.11.14 | 把电报译好后再送给收报人 |
| 223 | 1961.11.16 | 日本共产党中央决定　出版毛泽东选集日译本 |
| 224 | 1961.11.21 | 翻译出版中国书籍 |
| 225 | 1961.11.29 | 译印毛泽东选集日文版合同在京签订 |
| 226 | 1961.11.29 | 毛泽东选集第四卷日文版译完　廖承志同志举行庆祝酒会并欢送安斋库治同志等 |
| 227 | 1962.01.06 | 福建师院编译帝国主义侵略福建资料 |
| 228 | 1962.01.27 | 文化部和民委会邀集民族语文翻译工作者座谈　讨论提高“毛泽东选集”译文质量 |
| 229 | 1962.02.25 | 谈谈机器翻译 |
| 230 | 1962.05.22 | 黑格尔著《精神现象学》（上卷）中译本出版 |
| 231 | 1962.05.27 | 划时代的翻译事业－－谈“毛泽东选集”第四卷日文版翻译工作 |
| 232 | 1962.06.14 | 关于“民族”一词的使用和翻译情况 |
| 233 | 1962.06.14 | “民族”一词的译名统一问题的讨论 |

| | | |
|---|---|---|
| 234 | 1962.06.14 | 僮族长篇抒情诗译成汉文初稿 |
| 235 | 1962.06.24 | 古马亚文手稿译释本 |
| 236 | 1962.06.28 | 资产阶级古典政治经济学著作的翻译出版概况 |
| 237 | 1962.08.07 | 卢梭著作在我国的翻译和出版情况 |
| 238 | 1962.08.19 | 剧本评点－－一个中译英文独幕剧的前言 |
| 239 | 1962.09.05 | 全国人大常委会举行第六十二次会议　听取张致祥关于毛泽东著作的翻译、出版和发行情况的报告 |
| 240 | 1962.09.10 | 云南少数民族翻译干部正在成长 |
| 241 | 1962.09.10 | 上海译完两部古巴故事影片 |
| 242 | 1962.09.17 | 阿尔巴尼亚翻译出版《毛泽东论文学与艺术》 |
| 243 | 1962.10.19 | 政协文教组举行编译工作者座谈会 |
| 244 | 1962.10.30 | 从无神论著作的译本说起 |
| 245 | 1962.12.02 | 蒙古族英雄史诗《江格尔传》译成汉文 |
| 246 | 1962.12.18 | 艾地同志近作两本翻译出版 |
| 247 | 1963.02.01 | 阿尔巴尼亚翻译出版《杜甫诗选》 |
| 248 | 1963.03.05 | 《再论陶里亚蒂同志同我们的分歧》出版单行本　红旗杂志人民日报的四篇文章和社论译成少数民族文字 |
| 249 | 1963.03.21 | 内蒙古医学院编译完成《蒙药学》 |
| 250 | 1963.03.29 | 伏契克著作译成多种文字出版 |
| 251 | 1963.05.01 | 《金日成选集》第六卷中译本出版 |
| 252 | 1963.05.05 | 我国重视马克思恩格斯著作的翻译出版 |
| 253 | 1963.05.30 | 一批故事影片译成蒙古语片 |
| 254 | 1963.06.22 | 云南翻译纳西族文化遗产《东巴经》 |
| 255 | 1963.09.09 | 《金日成选集》第五卷译成中文出版 |
| 256 | 1963.10.24 | 三部古农书校译完毕 |
| 257 | 1964.01.06 | 《特殊任务》的译制工作做得好 |
| 258 | 1964.01.11 | 《野坂参三选集》战时篇中译本出版 |
| 259 | 1964.01.26 | 《金日成选集》第四卷中译本出版 |
| 260 | 1964.02.26 | “美国援助不发达国家的‘大神话’”－－摘译自二月十三日美国《国民前卫》周刊 |
| 261 | 1964.03.18 | 毛主席支持巴拿马人民谈话　意大利出版社译成意文出版 |
| 262 | 1964.03.25 | 印度尼西亚翻译出版鲁迅选集 |
| 263 | 1964.04.08 | 关于《共产党宣言》的一处译文 |
| 264 | 1964.05.02 | 毛主席著作《论人民民主专政》译本在锡出版 |
| 265 | 1964.05.17 | 锡兰翻译毛主席两篇著作　《论人民民主专政》已经译成僧伽罗文出版，《中国革命和中国共产党》正在继续翻译 |
| 266 | 1964.06.06 | 《白毛女》和《党的女儿》译成非洲语　文化部向配 |

| | | |
|---|---|---|
| | | 音的马里艺术家赠感谢状 |
| 267 | 1964.06.08 | 《红岩》被译成日本盲文 |
| 268 | 1964.07.08 | 无产阶级在其自发斗争阶段是否反对资产阶级的生产关系？——关于《共产党宣言》一处译文的意见 |
| 269 | 1964.07.09 | 毛主席著作《中国社会各阶级的分析》锡兰译成泰米尔文出版受读者热烈欢迎 |
| 270 | 1964.07.20 | 我翻译出版《战斗的南越》诗集 |
| 271 | 1964.07.27 | 《毛泽东选集》第四卷译成盲文出版 |
| 272 | 1964.09.07 | 新建北京外语专科学校开学　培养归国华侨学生为中学外语师资和翻译人材 |
| 273 | 1964.09.18 | 成功的尝试，友谊的结晶——记影片《白毛女》和《党的女儿》邦巴拉语译制工作 |
| 274 | 1964.09.20 | 柏拉图著作的翻译出版情况 |
| 275 | 1964.11.14 | 瑞典翻译出版《鲁迅选集》 |
| 276 | 1965.02.02 | 平托律师辩护词揭露美蒋集团直接策动和参与对我人员迫害　美蒋伪造信件歪曲译文手法卑鄙之极我人员行为正当非造谣诽谤所能抹黑 |
| 277 | 1965.05.20 | 约翰逊日子难过——摘译自五月十六日美国《时代》周刊 |
| 278 | 1965.06.03 | 《关于国际共产主义运动总路线的论战》译成四种少数民族文字即将出版 |
| 279 | 1965.06.05 | 杭州制成晶体管八路同声传译设备 |
| 280 | 1965.08.01 | 学习阮文追烈士的光辉榜样　介绍烈士事迹的《象他那样生活》一书已由我人民文学出版社翻译出版 |
| 281 | 1965.09.04 | 新共机关刊物全文刊载　毛主席著作《人的正确思想是从那里来的？》　毛主席两著作译成僧伽罗文在锡兰出版　《毛泽东军事文选》瑞典文版在瑞典发行 |
| 282 | 1965.09.11 | 毛主席又一著作译成僧伽罗文在锡兰出版 |
| 283 | 1965.11.16 | 关于“对抗消失了，矛盾还会存在”一语的译法 |
| 284 | 1965.12.17 | “危险的信号”——摘译自十二月六日《纽约先驱论坛报》 |
| 285 | 1965.12.30 | 澳共（马列主义者）主席希尔著作　《回顾与展望》中译本即将出版 |
| 286 | 1966.02.18 | 美国征兵难——摘译自二月四日美国《时代》周刊 |
| 287 | 1966.02.21 | 意《东风》季刊登载毛主席著作　还译载了中国报刊有关政治经济文化科学的文章 |
| 288 | 1966.03.19 | 《毛泽东同志论帝国主义和一切反动派都是纸老虎》　已译成柬文并在金边出版和发行 |
| 289 | 1966.03.31 | 谈马克思关于两种权利的一段话及其译文 |
| 290 | 1966.04.16 | 毛主席词《沁园春》(长沙)　伊拉克诗人译成阿拉 |

| | | |
|---|---|---|
| | | 伯文发表 |
| 291 | 1966.04.17 | 《中国文学》英文版和法文版　发表毛主席十首诗词的英译文和法译文 |
| 292 | 1966.05.06 | 日本人民赞王杰学王杰　广大读者热烈欢迎《王杰日记》在东京翻译出版　表示要学习王杰革命精神同美帝和修正主义斗争 |
| 293 | 1966.06.21 | 毛主席诗词十八首　译成阿拉伯文在叙利亚出版 |
| 294 | 1966.07.26 | 阿尔巴尼亚《十一月》杂志译载毛主席诗四首 |
| 295 | 1966.09.04 | 毛主席著作《关心群众生活，注意工作方法》在锡兰译成泰米尔文出版 |
| 296 | 1967.01.24 | 毛主席的光辉著作“老三篇”在锡兰翻译成僧伽罗文出版 |
| 297 | 1967.03.04 | 法国《新人道报》发表“老三篇”　毛主席三篇光辉著作在印度译成马拉雅兰文出版 |
| 298 | 1967.06.01 | 触说赵太后（译文） |
| 299 | 1967.10.11 | 毛主席的话句句是真理威力无穷　《毛主席语录》在开罗和冰岛出版受到革命人民热烈欢迎　毛主席光辉诗词在巴基斯坦译成孟加拉文出版 |
| 300 | 1968.03.30 | 智利一革命组织出版毛主席“老三篇”等光辉著作把“老三篇”运用到革命斗争中去　毛主席光辉著作译成僧伽罗文出版受到锡兰革命人民热烈欢迎 |
| 301 | 1968.03.31 | 毛泽东思想是光辉灿烂的太阳　《毛泽东选集》第一卷日文版新译本在日本受到热烈欢迎 |
| 302 | 1968.05.22 | 日本东京、山口县革命人民集会热烈庆祝《毛泽东选集》第一卷日文版新译本在日本发行　毛主席把马列主义发展到崭新的阶段　决心把毛泽东思想用到日本革命实践中去，为日本革命胜利而奋斗 |
| 303 | 1968.06.23 | 日本京都革命人民隆重集会热烈庆祝《毛泽东选集》日文版新译本在日本发行　毛泽东思想是战胜帝修反的最锐利思想武器　革命人民决心结合斗争实践努力学习和运用毛泽东思想 |
| 304 | 1968.07.30 | 《毛泽东选集》第一卷西班牙文版在拉美发行受到革命人民热烈欢迎　毛主席光辉著作在东巴基斯坦译成孟加拉文出版 |
| 305 | 1968.10.17 | 霍查同志三篇重要讲话译成中文将在我国全国各地陆续发行　阿《人民之声报》三月二十四日重要文章同时出版 |
| 306 | 1968.11.02 | 毛主席两篇光辉著作在锡兰翻译出版 |
| 307 | 1968.11.04 | 《中国共产党第八届扩大的第十二次中央委员会全会公报》用五种少数民族文字翻译出版 |
| 308 | 1968.12.01 | 毛主席的光辉著作在巴基斯坦出版　《论人民民主 |

| | | |
|---|---|---|
| | | 专政》、《毛主席论人民战争》译成孟加拉文在达卡出版　毛主席的亲密战友林副主席重要著作《人民战争胜利万岁》译成孟加拉文出版 |
| 309 | 1969.01.23 | 《红灯记》剧本翻译成阿文本　影片《钢琴伴唱〈红灯记〉》在地拉那上映受到广大群众欢迎 |
| 310 | 1969.01.29 | 伟大领袖毛主席的光辉诗篇《毛主席诗词》译成蒙古、藏、朝鲜三种少数民族文字出版 |
| 311 | 1969.03.05 | 《毛泽东著作选读》(甲种本)在锡兰翻译成僧伽罗文出版　译者赞扬毛主席是当代最伟大的马克思列宁主义者，毛主席著作是一切革命人民的宝书 |
| 312 | 1969.03.30 | 革命现代京剧样板戏《红灯记》剧本已译成尼泊尔文在加德满都出版 |
| 313 | 1969.06.09 | 《毛主席语录》西班牙文版在墨西哥出版　林副主席的政治报告和中共党章在锡兰译成当地语文出版 |
| 314 | 1969.06.21 | 热烈的祝贺　阿尔巴尼亚纪录影片　上海革命电影译制厂译制 |
| 315 | 1969.07.22 | 毛主席四篇光辉著作在印度出版　林副主席的政治报告和中共党章孟加拉文译本在印出版 |
| 316 | 1969.10.01 | 随着世界无产阶级和各国人民革命运动的蓬勃发展各国革命人民积极翻译出版毛主席著作　毛主席著作已在六十个国家和地区用七十种文字翻译出版一千多种版本 |
| 317 | 1969.12.25 | 拉美革命人民积极出版毛主席光辉著作　近几年内拉美革命人民冲破帝修反重重迫害翻印和翻译出版的毛主席著作达一百多种版本　拉美革命者指出："传播毛泽东思想，就是推动革命前进" |
| 318 | 1970.04.02 | 毛主席光辉著作《反对本本主义》在加德满都译成尼泊尔文出版　受到尼泊尔人民的热烈欢迎 |
| 319 | 1970.05.26 | 毛主席庄严声明《全世界人民团结起来，打败美国侵略者及其一切走狗!》已译成蒙古、藏等五种少数民族文字出版 |
| 320 | 1970.09.15 | 《中国共产党第九届中央委员会第二次全体会议公报》用五种少数民族文字和十一种外国文字翻译出版 |
| 321 | 1970.09.20 | 毛主席的光辉著作《论政策》已译成僧伽罗文在锡兰出版 |
| 322 | 1970.10.06 | 毛主席的光辉著作和庄严声明在锡兰翻译出版受到热烈欢迎　我党九届二中全会公报在锡兰翻译出版 |
| 323 | 1971.04.01 | 《无产阶级专政胜利万岁》一书已用蒙古、藏、维吾尔、哈萨克、朝鲜等少数民族文字翻译出版 |
| 324 | 1971.12.26 | 我国用五种少数民族文字翻译出版《共产党宣言》 |

| | | |
|---|---|---|
| 325 | 1972.04.22 | 毛主席诗词意文译本在意大利出版发行 |
| 326 | 1972.08.11 | 我驻赞临时代办侯启文和翻译张鑫奎不幸逝世　赞比亚总统卡翁达、副总统乔纳等先后到我使馆吊唁 |
| 327 | 1972.11.07 | 为了适应广大党员、干部、工农兵和革命知识分子看书学习的需要　马克思恩格斯列宁斯大林著作近两年来在我国大量编译出版 |
| 328 | 1973.04.20 | 原蒋帮陆军少校、中华航空公司编译　赵明哲先生起义归来 |
| 329 | 1973.07.26 | 广西各级电影部门积极发展少数民族地区电影放映事业 |
| 330 | 1973.09.09 | 金日成主席的讲话《进一步加强我国社会主义制度》和《朝鲜民主主义人民共和国社会主义宪法》中译本出版 |
| 331 | 1973.12.09 | 叙利亚翻译出版鲁迅作品 |
| 332 | 1974.08.11 | 新疆人民出版社翻译出版少数民族文字的批林批孔书籍 |
| 333 | 1974.10.06 | 《在社会主义大道上前进》社论用蒙古、藏、维吾尔、朝鲜、哈萨克文翻译出版 |
| 334 | 1974.12.03 | 日本共产党（左派）中央　重新翻译出版《共产党宣言》 |
| 335 | 1975.07.24 | 阿、罗翻译出版我小说《闪闪的红星》 |
| 336 | 1975.10.10 | 金日成同志两篇讲话中译本出版 |
| 337 | 1975.11.09 | 马克思《数学手稿》译成中文出版 |
| 338 | 1975.11.21 | 西藏青海等五省、区召开藏文图书翻译出版协作会议　决定翻译出版更多更好的藏文读物 |
| 339 | 1976.04.30 | 《毛主席诗词》英译本出版 |
| 340 | 1976.08.12 | 我国蒙古文图书编译出版事业发展快 |
| 341 | 1976.11.06 | 巴基斯坦和孟加拉国继续举行活动悼念毛主席　毛主席为被压迫人民作出杰出贡献　埃塞俄比亚出版毛主席著作的阿姆哈拉语译本 |
| 342 | 1977.05.09 | 《独立的罗马尼亚。1877》中译本出版 |
| 343 | 1977.06.23 | 华主席叶副主席接见《毛泽东选集》第五卷翻译出版人员少数民族学员解放军学员等八千多人 |
| 344 | 1977.07.04 | 华主席叶副主席会见并宴请参加《毛泽东选集》第五卷翻译工作的外国专家　李先念、汪东兴同志一起会见并宴请　会见和宴会充满亲切友好的气氛 |
| 345 | 1977.08.10 | 马克思：《机器。自然力和科学的应用》（手稿摘译）在《自然科学争鸣》杂志发表 |
| 346 | 1977.09.21 | 华主席的讲话和文章在挪威翻译出版 |
| 347 | 1977.10.04 | 波尔布特同志在金边庆祝柬共成立十七周年大会上讲话译成中文将出版 |

| | | |
|---|---|---|
| 348 | 1977.10.19 | 希望重印鲁迅译著及其它 |
| 349 | 1977.11.14 | 希腊《人民斗争》周刊发表文章指出　毛主席三个世界理论以马列主义为基础　《毛泽东选集》第五卷希腊文版在希腊翻译出版 |
| 350 | 1977.11.22 | 内蒙古用蒙、汉文字编译出版一批科技图书 |
| 351 | 1977.12.12 | “资产阶级法权”应改译为“资产阶级权利” |
| 352 | 1978.04.09 | 《处女地》译后记 |
| 353 | 1978.04.16 | 波尔布特同志在庆祝柬共成立　十七周年大会上报告的中译本出版 |
| 354 | 1978.05.11 | 《红楼梦》译成维吾尔文出版 |
| 355 | 1978.05.17 | 罗马尼亚翻译出版我国唐朝著名诗人的作品 |
| 356 | 1978.07.25 | 业余时间译稿是“搞自留地”吗？ |
| 357 | 1978.08.09 | 铁托同志在南共联盟十一大的报告和闭幕词翻译出版 |
| 358 | 1978.08.09 | 齐奥塞斯库同志在罗共中央全会上的讲话翻译出版 |
| 359 | 1978.10.14 | 马克思主义经典著作翻译家曹葆华同志病逝 |
| 360 | 1978.10.23 | 教育部、财政部、国家劳动总局联合发出通知　规定高等学校教师兼课和编译教材应给予报酬 |
| 361 | 1978.12.01 | 《统一的罗马尼亚民族国家的形成》中译本出版 |
| 362 | 1979.01.04 | 江苏成立技术资料翻译复制公司 |
| 363 | 1979.02.14 | 呼和浩特市组织业余翻译网 |
| 364 | 1979.02.21 | 《纪念爱因斯坦译文集》出版发行 |
| 365 | 1979.03.05 | 英译本《周恩来诗选》在香港出版 |
| 366 | 1979.03.31 | 大力促进文学翻译工人的发展 |
| 367 | 1979.04.10 | 日本翻译出版诗集　《周总理青少年时代的诗十四首》 |
| 368 | 1979.04.28 | 著名翻译家傅雷同志追悼会在上海举行 |
| 369 | 1979.05.08 | 《红楼梦》英文全译本出版 |
| 370 | 1979.09.23 | 毛泽东同志《同音乐工作者的谈话》一书已用五种少数民族语文翻译出版 |
| 371 | 1979.10.03 | 叶剑英同志《在庆祝中华人民共和国成立三十周年大会上的讲话》出版　还将用五种少数民族语文翻译出版 |
| 372 | 1979.11.08 | 中国翻译出版服务公司在北京成立 |
| 373 | 1979.11.18 | 雕像与木偶——伏契克《绞刑架下的报告》译后随想 |
| 374 | 1979.11.24 | 中国对外翻译出版公司成立 |
| 375 | 1979.12.14 | 《世界经济译丛》第十期出版 |
| 376 | 1979.12.15 | 《新译简注“蒙古秘史”》出版 |
| 377 | 1979.12.19 | 外国文学丛刊《译林》创刊 |

| | | |
|---|---|---|
| 378 | 1979.12.22 | 已故美国著名作家埃德加·斯诺所著　《西行漫记》重译本出版 |
| 379 | 1980.01.04 | 双腿残疾志更坚　刻苦自学攻英语　朱泱正式担任商务印书馆译著编辑 |
| 380 | 1980.01.28 | 《未来预测学译文集》出版 |
| 381 | 1980.02.26 | 外国哲学名著丛书编委会讨论翻译工作 |
| 382 | 1980.03.03 | 《翻译通讯》正式创刊 |
| 383 | 1980.03.13 | 《宪章运动史》翻译出版 |
| 384 | 1980.03.22 | 放在口袋里的翻译机 |
| 385 | 1980.04.17 | 《满文老档》的整理和翻译 |
| 386 | 1980.04.24 | 美《科学技术百科全书》译本即将出版 |
| 387 | 1980.05.12 | 青年译者的劳作 |
| 388 | 1980.06.10 | 做烧饼的工人当上了大学英语翻译（图片） |
| 389 | 1980.06.14 | 辽宁成立科学技术翻译公司广泛联系翻译人才　将在职的、业余的及闲散的翻译人员组织起来，为科研、生产和外贸服务 |
| 390 | 1980.07.14 | 《东西方的经济计划》中译本即将出版 |
| 391 | 1980.07.22 | 亚里士多德著《动物志》翻译出版 |
| 392 | 1980.07.25 | 《日中两千年》中译本出版 |
| 393 | 1980.07.26 | 养殖蚯蚓科技资料正在编译 |
| 394 | 1980.07.28 | 黑格尔《自然哲学》翻译出版 |
| 395 | 1980.08.11 | 英文翻译陶祖骥利用业余时间搭桥引路　促进中外技术交流　扩大对外贸易 |
| 396 | 1980.08.21 | 《经济学和公共目标》翻译出版 |
| 397 | 1980.08.21 | 我党忠诚的无产阶级革命战士、原中央编译局副局长　陈昌浩追悼会在北京举行 |
| 398 | 1980.08.25 | 《日本经济增长讲话》翻译出版 |
| 399 | 1980.08.25 | 不列颠百科全书中译本（简编）将在我国陆续出版 |
| 400 | 1980.09.04 | 《历代政治人物传记译注》将重印 |
| 401 | 1980.09.11 | 朱光潜委员希望　重新校译马列经典著作 |
| 402 | 1980.09.18 | 翻译界也来个“百花齐放” |
| 403 | 1980.10.06 | 《琉善哲学文选》选译出版 |
| 404 | 1980.11.06 | 商务印书馆将重印严译名著八种 |
| 405 | 1980.11.11 | 马坚译《古兰经》即将出版 |
| 406 | 1980.11.15 | 《中国震撼世界》翻译出版 |
| 407 | 1980.11.20 | 复员战士管豪自学当上翻译 |
| 408 | 1980.11.28 | 毛泽东诗词英语新译本《磅礴》在港出版 |
| 409 | 1980.12.18 | 重视国产故事片的译制工作 |
| 410 | 1981.01.12 | 商务印书馆辑印《汉译世界学术名著丛书》 |

| | | |
|---|---|---|
| 411 | 1981.01.25 | 售货员宗永强利用工余翻译地质专著　广州地理研究所破格吸收他进所工作 |
| 412 | 1981.02.03 | 《近代心理学历史导引》中译本出版 |
| 413 | 1981.02.14 | 奥斯托依奇大使举行酒会　庆祝《铁托选集》中译本出版 |
| 414 | 1981.03.23 | 《论德国》翻译出版 |
| 415 | 1981.03.30 | 重视对茅盾业绩的研究——《黎明时期的文学——中国现实主义作家·茅盾》译后记 |
| 416 | 1981.04.10 | 巴金的名著《家》译成德文出版 |
| 417 | 1981.04.10 | 我国最近有一批图书译成外文出版 |
| 418 | 1981.04.20 | 《美学译文》第一辑问世 |
| 419 | 1981.05.02 | 《科学小说译丛》出版 |
| 420 | 1981.05.09 | 《罗马尼亚共产党纲领》中译本出版 |
| 421 | 1981.05.09 | 介绍《电影艺术译丛》 |
| 422 | 1981.07.11 | 《傅雷译文集》将出版 |
| 423 | 1981.07.16 | 《花城译作》 |
| 424 | 1981.07.17 | 《中国共产党中央委员会关于建国以来党的若干历史问题的决议》等三个文件译成外文出版 |
| 425 | 1981.08.17 | 中文版《黑格尔全集》编译委员会在京成立 |
| 426 | 1981.08.25 | 《日本刑法、日本刑事诉讼法、日本律师法》翻译出版 |
| 427 | 1981.09.04 | 苏联翻译出版《鲁迅选集》 |
| 428 | 1981.09.18 | 日本八旬老人译出我古典医书《黄帝内经》 |
| 429 | 1981.10.05 | 《亚里士多德的三段论》出版中译本 |
| 430 | 1981.10.06 | 跛少年的译作 |
| 431 | 1981.10.09 | 《现代世界伦理学》编译出版 |
| 432 | 1981.10.12 | 《左传译文》出版 |
| 433 | 1981.10.24 | 青海省电影译制厂积极为少数民族服务 |
| 434 | 1981.11.01 | 我大使向马耳他总统赠送总统诗集中译本 |
| 435 | 1981.11.19 | 《傅雷译文集》简介 |
| 436 | 1981.11.22 | 《红楼梦》法文译本在巴黎出版 |
| 437 | 1981.12.08 | 在日本翻译出版新版《鲁迅全集》　人民文学出版社与日本学习研究社、曙光社在北京签订合同 |
| 438 | 1981.12.08 | 《鲁迅著译系年目录》 |
| 439 | 1981.12.20 | 金日成同志在朝鲜劳动党六大上的报告中译本出版 |
| 440 | 1982.02.01 | 《张闻天早年译剧选》序 |
| 441 | 1982.02.05 | 写在《汉译世界学术名著丛书》刊行之际 |
| 442 | 1982.03.16 | 民族出版社翻译出版一批文学名著 |
| 443 | 1982.03.31 | 内蒙古自治区重视蒙语影片译制和发行工作 |

| | | |
|---|---|---|
| 444 | 1982.04.05 | 《太平天国史译丛》第一辑出版 |
| 445 | 1982.06.24 | 中国翻译工作者协会在京成立　王震乌兰夫等出席并讲话 |
| 446 | 1982.08.11 | 译错两个字　供书受影响 |
| 447 | 1982.11.24 | 为藏民服务的译电员季克栋 |
| 448 | 1983.01.05 | 外语学院招收联合国译员训练班学员 |
| 449 | 1983.02.07 | 从《新水浒》想到《译报》 |
| 450 | 1983.02.22 | 翻译外国文学作品浅见 |
| 451 | 1983.03.09 | 中国对外翻译出版公司建立十周年 |
| 452 | 1983.03.15 | 中国笔会中心组织翻译塞浦路斯五篇小说 |
| 453 | 1983.03.15 | 马克思恩格斯文艺论著在中国的翻译和出版 |
| 454 | 1983.04.19 | 提高文学翻译质量　扶持文学翻译人才　《译林》《外国语》杂志举办译文征文评奖 |
| 455 | 1983.05.16 | 《世界史纲》新译本出版 |
| 456 | 1983.05.21 | 杨尚昆伍修权指出：翻译工作者要为国家多作贡献 |
| 457 | 1983.05.24 | 四千年前的哈拉帕“印章文字”初步破译 |
| 458 | 1983.07.31 | 我国成批培养符合联合国要求的高级专门翻译人才 |
| 459 | 1983.08.02 | 《圣经》被译成 1，763 种语言和方言 |
| 460 | 1983.08.17 | 不用翻译的一次国际会议－－第六十八届国际世界语大会侧记 |
| 461 | 1983.08.30 | 日本一份古代珍贵史料已译出 |
| 462 | 1983.10.12 | 著名文学翻译家、鲁迅研究专家孙用逝世 |
| 463 | 1983.10.17 | 《西方哲学研究名著丛书》正筹备编译 |
| 464 | 1983.11.09 | 获奖报告文学《中国姑娘》在日本翻译出版 |
| 465 | 1983.11.16 | 中国译协和北京市译协举行翻译界人士座谈会 |
| 466 | 1983.12.13 | 哥伦比亚《时代报》刊载　贝坦库尔总统翻译的毛主席词 |
| 467 | 1983.12.14 | 四川用藏、彝语言译制中外影片 |
| 468 | 1983.12.15 | 联合国委托开办的第六期译训班明年初招生 |
| 469 | 1983.12.25 | 《刘少奇选集》（上卷）译成五种外文 |
| 470 | 1983.12.28 | 微电脑控制的汉字自动译报机制成 |
| 471 | 1983.12.31 | 《邓小平文选》日译本（北京版）开始发行 |
| 472 | 1983.12.31 | 外国地名译音应统一 |
| 473 | 1984.01.06 | 朝鲜翻译出版我国小说《红岩》 |
| 474 | 1984.01.16 | 播种友谊的人－－记国际旅行社西安分社翻译导游鱼新平 |
| 475 | 1984.01.17 | 初译者的怀念 |
| 476 | 1984.01.19 | 全国翻译导游工作会议结束　万里陈慕华谷牧亲切会见全体代表 |
| 477 | 1984.01.23 | 袖珍电子翻译器 |

| | | |
|---|---|---|
| 478 | 1984.01.27 | 《江格尔》汉译本 |
| 479 | 1984.02.17 | 《我的根在中国》中译本序 |
| 480 | 1984.02.23 | 谈谈我的翻译工作 |
| 481 | 1984.03.14 | 庆祝张仲实翻译研究马列著作五十周年 |
| 482 | 1984.03.26 | 国家旅游局决定追认鱼新平为“全国模范翻译导游”在旅游系统开展学习鱼新平活动 |
| 483 | 1984.04.06 | 适应当前需要　我国自行编译　新版《列宁全集》今年开始出版　全集共六十卷，年内出版四卷，一九九〇年出齐 |
| 484 | 1985.04.10 | 北京市宣武医药卫生技术服务中心　科技翻译函授部招生 |
| 485 | 1984.04.13 | 中年工程师王同亿用十三种外语编译十二部科技词典 |
| 486 | 1984.05.07 | 《大趋势》中译本出版 |
| 487 | 1984.05.18 | 介绍《文幼章传》中译本 |
| 488 | 1984.05.25 | 《太平天国史译丛》简介 |
| 489 | 1984.06.15 | 我国已为联合国培养一百名高级翻译人员 |
| 490 | 1984.06.18 | 《世界文学名著新译丛书》 |
| 491 | 1984.07.04 | 美国日本制成新的自动翻译机并投放市场 |
| 492 | 1984.07.10 | 计算机藏文字处理系统研制成功　西藏译制七部藏语影片 |
| 493 | 1984.07.31 | 计算机翻译前景乐观 |
| 494 | 1984.08.19 | 访欧洲译者之家 |
| 495 | 1984.08.27 | 鲁迅与《三人》译本 |
| 496 | 1984.09.17 | 对外友协等庆祝《罗摩衍那》中文全译本出版 |
| 497 | 1984.09.23 | 《邓小平文选》英译本出版 |
| 498 | 1984.09.24 | 我国自行编译的第一部《列宁全集》一至四卷出版 |
| 499 | 1984.10.03 | 日本研制成自动翻译系统 |
| 500 | 1984.11.24 | 全国少数民族语译制片表彰大会在京举行　乌兰夫等到会表示祝贺 |
| 501 | 1984.11.30 | 联合国译训班第七期将招生 |
| 502 | 1984.12.05 | 译者的眼光、出版社的气魄及其他 |
| 503 | 1984.12.12 | 《史记》翻译、注释工作正加紧进行 |
| 504 | 1984.12.17 | 李健吾译的《莫里哀喜剧》 |
| 505 | 1984.12.20 | 热心民族文化交流的人－－访维吾尔文版《唐诗一百首》译者亚森·阿瓦孜 |
| 506 | 1984.12.21 | 二十四史中有关新疆部分正被翻译成维吾尔文 |
| 507 | 1985.03.09 | 她把心留在祖国－－记旅日华侨翻译家林芳（俞馥英） |

| | | |
|---|---|---|
| 508 | 1985.03.29 | 南共联盟主席接见《铁托自述》中文译者 |
| 509 | 1985.03.30 | 《自然辩证法》第五个中译本出版 |
| 510 | 1985.04.10 | 北京市宣武医药卫生技术服务中心　科技翻译函授部招生 |
| 511 | 1985.04.16 | 从日译本《鲁迅全集》想起的 |
| 512 | 1985.04.23 | 在日译本《鲁迅全集》出版的时候 |
| 513 | 1985.05.07 | 《杜甫诗今译》*序 |
| 514 | 1985.05.11 | 《史记注译》序 |
| 515 | 1985.06.05 | 傅雷亲属向法国三个图书馆赠《傅雷译文集》 |
| 516 | 1985.07.02 | 我古代算经《九章算术》译成法文 |
| 517 | 1985.07.11 | 成如容易却艰辛——记卫生部中医研究院编译工作者杨本文 |
| 518 | 1985.07.16 | 日研制成功计算机日译英系统 |
| 519 | 1985.07.29 | 中青年成为文学翻译主力军　我国文学翻译事业空前繁荣 |
| 520 | 1985.08.14 | 全国民族语文翻译学术讨论会结束 |
| 521 | 1985.08.30 | 中国共产党优秀党员、老一辈马列著作翻译家　柯柏年遗体告别仪式在京举行 |
| 522 | 1985.09.16 | 战士“翻译官” |
| 523 | 1985.10.25 | 对列宁关于“爱国主义”的一处论述的译文的订正 |
| 524 | 1985.11.17 | 三部中医经典著作首次译成英文 |
| 525 | 1985.12.07 | 我国三部古代哲学著作被译成波斯文 |
| 526 | 1985.12.18 | 编译家郑易里研究成一种电脑汉字编码新方法 |
| 527 | 1985.12.25 | 我爱瞿译《海燕》 |
| 528 | 1986.01.07 | 北京外国语学院联合国译员训练部第八期即将招生 |
| 529 | 1986.01.20 | 湘版《散文译丛》将陆续问世 |
| 530 | 1986.02.24 | 介绍《美学译文丛书》 |
| 531 | 1986.03.10 | 杰出翻译家的丰碑——《傅雷译文集》出版 |
| 532 | 1986.03.19 | 北京市工业系统人才开发中心翻译部　为引进技术提供翻译服务 |
| 533 | 1986.04.22 | 自动翻译电话 |
| 534 | 1986.04.27 | 改革和开放给翻译工作带来了春天　中国译协第一次全国代表会议在京闭幕 |
| 535 | 1986.05.05 | 我国翻译出版界辛勤努力三十年　马恩全集五十卷中文版全部出齐　收入二千多篇著作，四千多封书信，四百多件文献资料 |
| 536 | 1986.05.14 | 杰出的语言学家、教育家、诗人和翻译家　王力教授遗体告别仪式在京举行 |
| 537 | 1986.05.18 | 翻译工作的发展和面临的新课题 |
| 538 | 1986.06.08 | 宏大的翻译工程　丰富的理论宝库 |

| | | |
|---|---|---|
| 539 | 1986.06.24 | 《诗苑译林》 |
| 540 | 1986.07.13 | 《冰心著译选集》自序 |
| 541 | 1986.07.20 | 《苏联军事百科全书》中译本出版 |
| 542 | 1986.07.21 | 一个伟大的民主主义者的自白－－斯诺家书选译 |
| 543 | 1986.07.22 | 一个伟大的民主主义者的自白－－斯诺家书选译 |
| 544 | 1986.07.23 | 一个伟大的民主主义者的自白－－斯诺家书选译 |
| 545 | 1986.08.02 | 北京发现斯诺《战时苏联游记》中译本 |
| 546 | 1986.08.07 | 《外国著名思想家译丛》开始出版 |
| 547 | 1986.09.01 | 欧洲共同体将研制电脑速译系统 |
| 548 | 1986.09.02 | 玛雅文字被破译 |
| 549 | 1986.09.06 | 中美合作编译的大型综合性工具书　《简明不列颠百科全书》中文版出齐 |
| 550 | 1986.09.11 | 《浮士德》的业余翻译家 |
| 551 | 1986.09.15 | 罗马尼亚出版《诗经》选译本 |
| 552 | 1986.09.26 | 促进比中文化交流　比翻译发行一期《人民日报》 |
| 553 | 1986.10.07 | 西班牙国王向翻译家杨绛、教师列塞亚授勋 |
| 554 | 1986.10.10 | 《朝鲜劳动党简史》中译本出版 |
| 555 | 1986.10.13 | 名著《亚玛街》翻译出版 |
| 556 | 1986.11.22 | 一期《人民日报》译成荷兰文出版 |
| 557 | 1986.11.24 | “资产阶级权利”的误译和误解 |
| 558 | 1986.11.28 | 费孝通《江村经济》译成中文出版 |
| 559 | 1986.11.28 | 编译队伍三百多人　明春出书二十余种　《二十世纪文库》将译介当代社科名著 |
| 560 | 1986.12.06 | 联合国委托我国开办的译员训练部再次招生 |
| 561 | 1986.12.20 | 埃洛杜伊翻译《诗经》荣获西班牙国家翻译奖 |
| 562 | 1986.12.26 | 《苏联百科词典》中译本即将出版 |
| 563 | 1987.01.07 | 新编剑桥世界近代史翻译完成　今年上半年开始分卷出版发行 |
| 564 | 1987.01.16 | 多年致力于翻译歌德和海涅著作　冯至教授在西德获奖 |
| 565 | 1987.01.18 | “经济学”译名的首创人－－孙中山 |
| 566 | 1987.01.25 | 融水苗族自治县电影公司立足苗乡　五人办个苗语“电影译制厂” |
| 567 | 1987.01.27 | 王贤才独立译《希氏内科学》巨著　卫生部授予他医学翻译工作特别奖 |
| 568 | 1987.02.09 | 捷翻译出版《红楼梦》 |
| 569 | 1987.02.15 | “经济学”译名的首创者并非孙中山 |
| 570 | 1987.02.26 | 新版《苏联百科词典》翻译出版 |
| 571 | 1987.02.28 | 有感于翻译设奖 |
| 572 | 1987.02.28 | 钱钟书的一些作品被译成法文 |

| | | |
|---|---|---|
| 573 | 1987.03.06 | 填补空白的新书——喜读《大唐西域记今译》 |
| 574 | 1987.03.18 | 诗评与译诗——与王佐良教授一席谈 |
| 575 | 1987.03.24 | 瑞典翻译发行《人民日报》 |
| 576 | 1987.03.26 | 瑶族史诗《密洛陀》译成汉文 |
| 577 | 1987.03.29 | 青海译制多部藏语影片 |
| 578 | 1987.04.02 | 我国第一台机器译英文模仿人思维 |
| 579 | 1987.04.02 | 一批马列著作毛泽东著作　用五种民族语文翻译出版 |
| 580 | 1987.04.03 | 上海电影译制厂 30 年译片近千部 |
| 581 | 1987.04.21 | “甘肃荣誉公民”艾黎自传中译本出版 |
| 582 | 1987.04.24 | 文学翻译改革漫议 |
| 583 | 1987.05.05 | 新疆每周译制两部少数民族语影片　维吾尔、哈萨克语新片能与汉语新片同时上映 |
| 584 | 1987.05.07 | 翻译苏联革命文学　影响几代有志青年　首都举行老作家曹靖华学术座谈会 |
| 585 | 1987.06.04 | 《艺术与幻觉》中译本将出版 |
| 586 | 1987.06.10 | 余音还在我心回荡——《劫后人语》中译记 |
| 587 | 1987.06.13 | 《建设有中国特色的社会主义》等两本书　用五种少数民族文字翻译出版 |
| 588 | 1987.06.19 | 我为联合国培养 160 多名高水平译员 |
| 589 | 1987.06.28 | 美国友人宋德史夫人译成《中国史漫游》　向我历史博物馆赠送 5000 册 |
| 590 | 1987.06.28 | 《武经七书注译》评介 |
| 591 | 1987.07.11 | 抗日战争中的《每日译报》 |
| 592 | 1987.07.28 | 女记者杨刚遗作《挑战》面世　英文自传小说　遗存美国多年　译成中文发表 |
| 593 | 1987.08.02 | 上马迎敌焰　下马治兵书——访《孙子》译注者郭化若将军 |
| 594 | 1987.08.06 | 我国著名翻译家曹靖华　获苏联各国人民友谊勋章 |
| 595 | 1987.08.12 | 我国近年译制藏语影片 250 多部 |
| 596 | 1987.08.14 | 英国电信局研制成功翻译电话 |
| 597 | 1987.08.15 | 人大常委会法制工作委员会主持编译　《中华人民共和国法律汇编》英文本出版 |
| 598 | 1987.08.21 | 民族语文翻译工作者聚集哲里木交流学术 |
| 599 | 1987.08.23 | 鲁迅与谷虹儿——谈新发现的一页鲁迅译稿 |
| 600 | 1987.09.08 | 著名翻译家、作家曹靖华逝世 |
| 601 | 1987.09.26 | 百年草叶何青青——读《草叶集》中文全译本 |
| 602 | 1987.10.18 | 给翻译文学以应有的地位 |
| 603 | 1987.10.19 | 40 多年前由孙承佩译成中文　斯诺《战时苏联游记》重新出版 |

| | | |
|---|---|---|
| 604 | 1987.10.22 | 《上海译报》明年改出四开八版 |
| 605 | 1987.10.25 | 上海应有高效率的编译所 |
| 606 | 1987.10.28 | 我国连环画《真假猴王》在波兰翻译出版 |
| 607 | 1987.11.06 | 中国翻译工作者协会已正式加入国际译联 |
| 608 | 1987.11.10 | 读戈宝权新译作《普希金诗集》 |
| 609 | 1987.11.10 | 苏联诗歌翻译座谈会在京举行 |
| 610 | 1987.12.02 | 《诺贝尔经济学奖获得者主要著作译丛》即将出版 |
| 611 | 1987.12.07 | 《人啊，人!》书名翻译 |
| 612 | 1987.12.11 | 中日联合开发多国语言自动翻译系统 |
| 613 | 1987.12.13 | 谁把 Philosophy 译为“哲学”的? |
| 614 | 1987.12.24 | 《文化人类学名著译丛》将出版 |
| 615 | 1988.01.07 | 著名翻译家和编辑出版家姜椿芳同志逝世 |
| 616 | 1988.01.13 | 著名翻译家戈宝权获普希金文学奖 |
| 617 | 1988.01.21 | 翻译误译引出笑声（图片） |
| 618 | 1988.01.28 | 四代五口人　译文七百万　彝族一农户是道地“翻译世家” |
| 619 | 1988.02.13 | 有关布哈林七种中译本将出版 |
| 620 | 1988.02.16 | 《天京之变》英译本在美出版 |
| 621 | 1988.02.22 | 马克思笔下《资本论》第一卷原貌　德文第一版首次在我国翻译出版 |
| 622 | 1988.02.22 | 青山情－－读《青山青》中译本 |
| 623 | 1988.03.16 | 我国翻译出版　戈尔巴乔夫的《改革与新思维》 |
| 624 | 1988.03.31 | “高级翻译” |
| 625 | 1988.04.08 | 为“荣老板”找“翻译” |
| 626 | 1988.04.23 | 中国有个董先生－－记中国国际旅行社翻译董应烈 |
| 627 | 1988.05.08 | 英国展出自动翻译电话系统 |
| 628 | 1988.05.09 | 印度名著中译本等两书出版　印驻华大使在京举行招待会 |
| 629 | 1988.05.13 | 《一个美国人看旧中国》中译本出版 |
| 630 | 1988.06.02 | 我国 8 年出版法学教材 111 种　《国际法》一书正被国外翻译出版 |
| 631 | 1988.06.09 | 挖掘研究民间艺术和民俗取得重大成果　靳之林破译一批原始文化符号 |
| 632 | 1988.06.20 | 石棚山原始图画文字得到破译　燕山得名由来殷商命名缘起有新依据 |
| 633 | 1988.06.29 | 自动翻译技术在法国复苏　6 种西方文字对译软件进入电脑 |
| 634 | 1988.07.01 | 影片《末代皇帝》译制完成 |
| 635 | 1988.07.30 | 陈拱诒瘫痪 30 多年翻译百万余字　获“残疾人自学成才”荣誉证书 |

| | | |
|---|---|---|
| 636 | 1988.07.31 | 发现八十八年无人读懂　敦煌舞谱破译有新突破 |
| 637 | 1988.08.11 | 《世界名著鉴赏大辞典》全部译成中文 |
| 638 | 1988.08.13 | 西德一研究人员声称：他破译了玛雅文字 |
| 639 | 1988.09.08 | 英汉翻译何处寻　智能机器有“译星”　一种英汉机器翻译系统应用广泛 |
| 640 | 1988.09.13 | 用少数民族文字翻译出版各类著作四百余 |
| 641 | 1988.10.23 | 中国古代的翻译 |
| 642 | 1988.10.23 | 《水浒》的译名 |
| 643 | 1988.10.25 | 中青年文学翻译交流会 |
| 644 | 1988.10.26 | 文学翻译事业喜中见忧　专家呼吁提高译本质量 |
| 645 | 1988.11.05 | 自动翻译技术在法国发展迅速　加绍公司推出六种西方文字对译软件 |
| 646 | 1988.11.17 | 《牛津法律大辞典》中译本出版 |
| 647 | 1988.11.30 | 《周易参同契》英译本出版 |
| 648 | 1989.01.09 | 《国际政治学汉译名著》丛书将出版 |
| 649 | 1989.01.25 | 牛津法律大辞典和它的译者 |
| 650 | 1989.01.31 | 中国艺术研究院首次评选研究成果　一批优秀专著、论文、评论、资料、译著获奖 |
| 651 | 1989.02.28 | 破译蜜蜂通讯密码　人造蜜蜂引来真蜂 |
| 652 | 1989.03.10 | 日试制出多种语言同译系统 |
| 653 | 1989.04.05 | 一部世界名著译本中包含着：苦涩、凄凉、辛酸　《静静的顿河》校译忆往 |
| 654 | 1989.04.14 | 文学作品进出口逆差　对外译介亟待加强 |
| 655 | 1989.05.03 | 赛珍珠《大地》三部曲中译本出版 |
| 656 | 1989.05.14 | 翻译家张锡俦逝世 |
| 657 | 1989.05.24 | 西夏封建政权以法治国　《西夏法典》译本出版 |
| 658 | 1989.07.19 | 邓小平论述和四中全会公报译成少数民族文字出版发行 |
| 659 | 1989.08.06 | 上海译文出版社数年如一日　置社会效益于首位以图书质量求读者 |
| 660 | 1989.08.08 | 罗选译出版中国古代小说 |
| 661 | 1989.09.09 | 中国第一部介绍马克思学说的译著——《泰西民法志》 |
| 662 | 1989.10.03 | 少数民族文学作品汉译培训班开学 |
| 663 | 1989.10.20 | 《二十世纪外国教育名著译丛》 |
| 664 | 1989.10.24 | 《史记注译》在西安问世 |
| 665 | 1989.10.31 | 贵州“彝族甲骨文”被破译 |
| 666 | 1989.11.24 | 钱玉趾破译“巴蜀符号”　为探索古蜀文明开辟了新径 |
| 667 | 1989.12.06 | 翻译公司的兴起 |

| | | |
|---|---|---|
| 668 | 1989.12.10 | 在马克思主义指导下吸收外国文化　学者座谈汉译世界学术名著　这部丛书对我国学术文化有基本建设意义 |
| 669 | 1989.12.14 | 缅作家译《红楼梦》获缅甸国家文学奖 |
| 670 | 1990.01.20 | 百年译事有专史－－读《中国翻译文学史稿》 |
| 671 | 1990.01.21 | 靠真本领下硬功夫节能　江泽民译著《机械制造厂电能的合理使用》出版 |
| 672 | 1990.02.05 | 译诗家飞白撰成《诗海》 |
| 673 | 1990.02.18 | 美国电话电报公司称　它能向全球提供电话同声翻译服务 |
| 674 | 1990.02.20 | 七种民族语文翻译出版　马列著作选读系列读物 |
| 675 | 1990.03.02 | 邓小平同志的重要论著译成多种民族文字出版 |
| 676 | 1990.03.25 | 优秀口译人才难得 |
| 677 | 1990.03.25 | 母女切磋同声翻译 |
| 678 | 1990.04.20 | 富于学术价值的译本－－读田德望译《神曲・地狱篇》 |
| 679 | 1990.04.25 | 小说《孔子》中译本近日出版 |
| 680 | 1990.05.29 | 有这么一处风景－－记靳建国和靳译罗素文集 |
| 681 | 1990.07.28 | 西游取回真经　历载精译成卷　唐玄奘译撰全集珍本出版 |
| 682 | 1990.08.08 | 七百年前蒙古族抒情诗　《金帐桦皮书》译成汉文 |
| 683 | 1990.08.22 | “五四”以来最大的古籍今译系列出版　《古代文史名著选译丛书》举行首发式 |
| 684 | 1990.08.23 | 译联第１２次世界大会召开　我代表当选为理事会理事 |
| 685 | 1990.09.09 | 李约瑟巨著《中国科学技术史》中译本发行 |
| 686 | 1990.09.21 | 为中国歌曲插上翅膀－－记亚运歌曲英文翻译张庆年 |
| 687 | 1990.10.04 | 记者当翻译 |
| 688 | 1990.11.13 | 别开生面的李贺诗“破译” |
| 689 | 1990.12.05 | 学者呼吁：对外宣传英译水平有待提高 |
| 690 | 1990.12.05 | 《围城》：第八种译本是电视 |
| 691 | 1990.12.08 | 大陆译的《资本论》在台出版 |
| 692 | 1991.01.02 | 汉字与盲文转译电脑化　盲人有望读到当日报纸 |
| 693 | 1991.01.10 | 《世界名著鉴赏大辞典》中文译本出版 |
| 694 | 1991.01.11 | 《希波克拉底文集》中译本问世 |
| 695 | 1991.03.27 | 同声传译 |
| 696 | 1991.03.31 | 范维信获巴西“国外最佳翻译奖” |
| 697 | 1991.04.07 | 《玛纳斯》柯尔克孜文本及汉译本出版 |

| | | |
|---|---|---|
| 698 | 1991.04.20 | 西藏电影译制业 |
| 699 | 1991.04.26 | 首都举行座谈会　庆祝新版《列宁全集》出版发行　李瑞环说这是马列经典著作编译出版的一大成果 |
| 700 | 1991.05.02 | 传递真理之火——中共中央马恩列斯著作编译局采访纪事 |
| 701 | 1991.09.11 | 电话翻译机 |
| 702 | 1991.10.25 | 《中国历代名著全译丛书》出版　精选先秦至清代经史子集名著５０种，１９９５年出齐 |
| 703 | 1991.11.11 | 这也是一种翻译艺术 |
| 704 | 1991.12.04 | 马克思《历史学笔记》译成中文　这部重要著作为后人了解马克思的历史观点和历史方法留下了重要的依据和丰富的材料 |
| 705 | 1991.12.04 | 由《文白对照全译〈资治通鉴〉》想起的 |
| 706 | 1991.12.07 | 唐宋破译歌曲将出选集 |
| 707 | 1991.12.11 | 面壁十余载　破译千古谜　尹博灵将石鼓文译成白话史诗 |
| 708 | 1991.12.16 | 柴屋灯火——致《共产党宣言》的第一个中译者陈望道先生 |
| 709 | 1992.01.04 | 六旬农民李全振译、唱京剧《甘露寺》 |
| 710 | 1992.01.05 | 马克思恩格斯著作翻译出版之最 |
| 711 | 1992.01.14 | 李鹏接见国旅导游翻译会议代表时说　中国旅游业发展潜力很大 |
| 712 | 1992.01.21 | 中宣部表彰《列宁全集》二版编译人员 |
| 713 | 1992.02.04 | 《二十六史精粹今译》获好评 |
| 714 | 1992.02.23 | 第一个把毛泽东著作译成英文的人 |
| 715 | 1992.02.24 | 一部展现历史画卷的珍贵文献——为马克思《历史学笔记（19 世纪 80 年代初）》中译本的问世而作 |
| 716 | 1992.03.11 | 莫云译者痴须解其中味——访中医典籍翻译罗希文 |
| 717 | 1992.04.04 | 再现伟大翻译家的生平 |
| 718 | 1992.04.08 | 首届日译中翻译奖在京揭晓 |
| 719 | 1992.04.11 | 译林梦寻——记译林出版社 |
| 720 | 1992.05.06 | 用精取宏图文并茂——插图本《世界文学史》编译感言 |
| 721 | 1992.05.09 | 纪伯伦全集中译本 |
| 722 | 1992.05.27 | 《石油·金钱·权力》译成中文 |
| 723 | 1992.06.12 | 佳木斯建翻译服务公司 |
| 724 | 1992.06.27 | 高立公司高起点开发高科技产品英汉、日汉电脑翻译系统问世 |
| 725 | 1992.07.19 | 智能型机器翻译系统通过鉴定 |
| 726 | 1992.08.31 | 《日本学者研究中国史论著选译》出版 |

| | | |
|---|---|---|
| 727 | 1992.10.12 | “天书”敦煌曲谱被破译 |
| 728 | 1992.10.15 | 唐诗好译本 |
| 729 | 1992.11.07 | 有翻译功能的电话 |
| 730 | 1992.11.08 | 意大利翻译出版郭沫若诗集 |
| 731 | 1992.12.06 | 马克思《历史学笔记》中译本翻译出版前后——纪念张友渔同志 |
| 732 | 1993.01.15 | 电话自动翻译系统试验成功 |
| 733 | 1993.01.28 | 具有翻译功能的新型复印机 |
| 734 | 1993.01.30 | “你好”变“哈罗”电话可自译 |
| 735 | 1993.02.21 | 谁能破译八种神秘文字 |
| 736 | 1993.02.22 | 马克思《历史学笔记》中译本翻译出版的前前后后——纪念张友渔同志 |
| 737 | 1993.03.02 | 马恩全集中文二版编译工作展开江泽民杨尚昆李鹏题词祝贺中央编译局成立四十周年 |
| 738 | 1993.03.18 | 中国对外翻译出版公司喜庆二十华诞 |
| 739 | 1993.03.20 | 女翻译和服务员（图片） |
| 740 | 1993.04.10 | 陈肇雄研制机器翻译有重大突破智能型机译理论技术创五个世界第一 |
| 741 | 1993.05.02 | 让更多的中国人了解萨马兰奇——写在《萨马兰奇与奥林匹克》中译本发行前夕 |
| 742 | 1993.05.14 | 中央人民广播电台启用新的英文译名 |
| 743 | 1993.05.29 | 重庆大学建成中国当代翻译家档案库 |
| 744 | 1993.06.12 | 《江格尔》汉文全译本出版 |
| 745 | 1993.06.15 | 郭沫若著译版本展举行 |
| 746 | 1993.06.26 | 《文白对照全译二十五史纲鉴》出版 |
| 747 | 1993.07.28 | 《毕译左拉名著文库》出版 |
| 748 | 1993.08.02 | 《中国古代名著今译丛书》开始面世 |
| 749 | 1993.08.09 | 德国研制口头翻译机 |
| 750 | 1993.08.12 | 真挚的朋友——《日中关系十八年》中译本序 |
| 751 | 1993.09.18 | 中软公司推出汉英汉日机器翻译系统 |
| 752 | 1993.10.22 | “天经”译哈文恩泽穆斯林《古兰经》有了哈萨克文版 |
| 753 | 1993.10.22 | 译著的诞生 |
| 754 | 1993.10.22 | 充满魅力的评赏新译 |
| 755 | 1993.10.25 | 《海外邓小平研究》译丛问世 |
| 756 | 1993.10.28 | 为人间增添一丝温暖——谈朱光潜译维柯《新科学》 |
| 757 | 1993.12.03 | 毕尽心血译左拉 |
| 758 | 1993.12.11 | 《美国法典》翻译出版 |
| 759 | 1993.12.17 | 漫谈“随笔译丛”的文化品质 |
| 760 | 1993.12.27 | 新型微机编译系统研制成功 |

| | | |
|---|---|---|
| 761 | 1993.12.27 | 李蕴创造破译甲骨文新方法 |
| 762 | 1994.01.05 | 《诸子精粹今译》出版 |
| 763 | 1994.01.25 | 同声翻译计算机将问世 |
| 764 | 1994.03.07 | 电话建议译名著满厅杜鹃添春色 |
| 765 | 1994.03.22 | 日推出智能型日英互译机 |
| 766 | 1994.04.19 | 英译《老子》《诗经》的人－－记大连外国语学院汪榕培教授 |
| 767 | 1994.04.22 | 商务印书馆致力于介绍各国思想文化精华汉译世界学术丛书出版３００种 |
| 768 | 1994.04.29 | 数学大师破译著名密码 |
| 769 | 1994.05.26 | 快译通杯网球赛举行 |
| 770 | 1994.06.11 | 《日本通商产业政策史》中译本出版 |
| 771 | 1994.06.14 | 中美两家出版单位在京签署版权协议《世界百科全书》中文版开始编译 |
| 772 | 1994.07.13 | 《白凡逸志》中译本出版 |
| 773 | 1994.07.15 | 《政治哲学史》首次出版中译本 |
| 774 | 1994.07.22 | 多功能飞行数据记录器译码设备问世 |
| 775 | 1994.07.29 | 全译《尤利西斯》 |
| 776 | 1994.08.02 | 迟到的辉煌－－《"毕译左拉"名译名著精选文库》小记 |
| 777 | 1994.08.02 | 译电员马虎　喜讯变噩耗 |
| 778 | 1994.09.20 | 诗琳通翻译中国小说《蝴蝶》在泰发行 |
| 779 | 1994.10.08 | 垒球赛后记者战翻译乒乓球场小将扮明星 |
| 780 | 1994.10.08 | "译星"又出新版本 |
| 781 | 1994.11.02 | 古籍今译岂可草率为之 |
| 782 | 1994.11.19 | 近千卷东巴象形古籍翻译完毕 |
| 783 | 1994.12.10 | 《三国演义》英文本问世中国古典小说四大名著英译本出齐 |
| 784 | 1995.01.16 | 欧盟语种多译桥忙不迭 |
| 785 | 1995.01.24 | 关于古籍今译问题 |
| 786 | 1995.02.01 | 开放的中国如何对待外来文化－－从《汉译世界学术名著丛书》的出版谈起 |
| 787 | 1995.02.04 | 杭州热心支持译制片事业 |
| 788 | 1995.02.09 | 千卷东巴古籍翻译完毕 |
| 789 | 1995.03.01 | "知识产权"的翻译是否正确 |
| 790 | 1995.04.03 | 引进一部外版书比派团出国考察便宜得多科技译著出版滑坡堪忧 |
| 791 | 1995.04.20 | 中外学者研讨《尤利西斯》萧乾姤姤叙说翻译经过 |
| 792 | 1995.05.13 | 关于名著重译问题 |
| 793 | 1995.05.29 | 精选中华文明史上最有代表性的一百种书，译成外 |

| | | |
|---|---|---|
| | | 文，推向海外给世界一个惊喜——《大中华文库》座谈会侧记 |
| 794 | 1995.06.01 | 戴妮丝遗体告别仪式在京举行李风白—戴妮丝优秀外文图书翻译基金即将建立 |
| 795 | 1995.06.25 | 精雕细刻十六年——访《文心雕龙》译者桑德拉·拉瓦尼诺 |
| 796 | 1995.06.25 | 音译词使用要规范化 |
| 797 | 1995.07.11 | 翻译出版界的一项大工程 |
| 798 | 1995.08.01 | 亚洲翻译家论坛在京举行 |
| 799 | 1995.08.29 | 加入国际版权公约后翻译书怎么办？电子出版社：对外合作出版大有可为 |
| 800 | 1995.09.02 | 非洲妇女手执翻译机 |
| 801 | 1995.10.16 | 英华缀出一座“世界精神公园”——《汉译世界学术名著丛书》出版３００种纪略 |
| 802 | 1995.10.17 | 愿将宏愿献余生——《伊利亚特》和译者罗念生 |
| 803 | 1995.10.24 | 我机器翻译研究有重大突破　英汉汉英计算机全文翻译系统问世并走向实用 |
| 804 | 1995.10.27 | 党的思想理论建设的重要工程——新版马列著作编译工作纪实 |
| 805 | 1995.10.27 | 马克思主义经典著作编译和研究最新成果新版马列著作出版 |
| 806 | 1995.10.28 | 马列著作编译事业的丰硕成果——祝贺新版马列著作出版发行 |
| 807 | 1995.10.29 | 安徒生童话故事全集新译本首发 |
| 808 | 1995.11.24 | 《堂吉诃德》新译本首发式暨研讨会在京举行 |
| 809 | 1995.12.04 | 部分作家、翻译家、出版工作者呼吁尊重著译者权利 |
| 810 | 1995.12.13 | 《爱因斯坦全集》翻译出版工程启动 |
| 811 | 1995.12.21 | 马列著作编译事业新成果（一）——《马克思恩格斯选集》中文第二版简介 |
| 812 | 1995.12.22 | 马列著作编译事业新成果（二）——《列宁选集》中文第三版简介 |
| 813 | 1995.12.26 | 马列著作编译事业新成果（三）——《马克思恩格斯全集》中文第二版简介 |
| 814 | 1996.01.30 | 学界高手移来他山之石《汉译世界学术名著丛书》出至２６０种 |
| 815 | 1996.03.07 | 贺《人间喜剧》全译本出版 |
| 816 | 1996.04.02 | 西安翻译培训学院育才逾万 |
| 817 | 1996.04.10 | 你可曾想起我——通译软件的自述 |
| 818 | 1996.04.28 | 他译中国文学——访乌克兰翻译家奇尔科 |

| | | |
|---|---|---|
| 819 | 1996.06.10 | 环球通汉英翻译软件面世 |
| 820 | 1996.06.29 | 全国第二次少数民族文学翻译会议在内蒙古赤峰召开 |
| 821 | 1996.08.06 | 翻译家、专家座谈翻译 |
| 822 | 1996.08.09 | “不配英文翻译”浅议 |
| 823 | 1996.08.22 | 亚俱杯申花显威七比一大胜快译通队 |
| 824 | 1996.09.05 | 亚洲俱乐部杯足球赛上海申花再胜快译通 |
| 825 | 1996.09.11 | 史诗《玛纳斯》汉译基本完成专家认为我国天山北部是《玛纳斯》的故乡 |
| 826 | 1996.11.01 | 汪榕培完成四部英译中国古代经典著作 |
| 827 | 1996.11.07 | 通译软件互译速度快 |
| 828 | 1996.11.11 | 《史记》韩文全译本在汉城出版 |
| 829 | 1996.11.18 | 四川推出英汉翻译机 |
| 830 | 1996.11.29 | 《中国第一王朝的崛起－－中华文明和国家起源之谜破译》 |
| 831 | 1996.12.08 | “卡拉ＯＫ”译名说 |
| 832 | 1997.01.22 | 精博兼顾雅俗共赏《科学大师佳作系列》中译本面世 |
| 833 | 1997.01.27 | 邮电局译错电报应否承担赔偿责任？ |
| 834 | 1997.02.05 | 反思科学与人类的命运－－“科学与人译丛”的探索 |
| 835 | 1997.02.12 | 《工商管理精要》译丛出版 |
| 836 | 1997.02.15 | 《经济学》第十四版翻译出版 |
| 837 | 1997.05.04 | 让历史作证－－江苏人民出版社加紧翻译出版《拉贝日记》 |
| 838 | 1997.05.14 | 通译之路 |
| 839 | 1997.06.26 | 香港所有成文法中文文本翻译完成中英文法律文本享有同等地位 |
| 840 | 1997.06.28 | 打官司不请翻译了 |
| 841 | 1997.08.18 | 中国古史中的“哥德巴赫猜想”何时破译 |
| 842 | 1997.08.25 | 西安翻译培训学院面向社会需求育人十年从严治教万名学子成才 |
| 843 | 1997.09.04 | 尘封六十年面世成证言《拉贝日记》翻译出版 |
| 844 | 1997.10.05 | 关于我的译文 |
| 845 | 1997.11.18 | 众体裁百花齐放多语种比翼齐飞第五届少数民族文学创作奖颁奖２４个民族的６０位作家、３位译作者获奖；珞巴、基诺、塔塔尔三个民族的作家作品首次上榜 |
| 846 | 1997.11.24 | 袖珍电译机首创成功 |
| 847 | 1997.11.27 | 儒家经典无国界希伯莱文译四书 |

| | | |
|---|---|---|
| 848 | 1997.12.10 | 了解美国文化的桥梁《蓝登大学词典》有了中译本 |
| 849 | 1997.12.13 | 《近代文史名著选译丛书》 |
| 850 | 1997.12.16 | 王金铃翻译实践研讨会在京召开 |
| 851 | 1998.01.12 | 《人权百科全书》翻译出版 |
| 852 | 1998.02.21 | 《共产党宣言》的署名和第一个英文译本 |
| 853 | 1998.03.05 | 让文化经典娓娓道来－－“野骆驼译丛”读后 |
| 854 | 1998.03.31 | 因特网翻译装置 |
| 855 | 1998.06.12 | 又一翻译软件问世 |
| 856 | 1998.07.02 | 为了唤起真正的自尊－－记英文版《南京暴行》中译本的问世 |
| 857 | 1998.07.13 | 天津“通译”支持信息工程 |
| 858 | 1998.07.26 | 译名的误区 |
| 859 | 1998.08.28 | 翻译陷于困境－－欧盟语言问题扫描之二 |
| 860 | 1998.09.29 | 诗琳通公主译作《妙笔生花》出版 |
| 861 | 1998.10.13 | 拓民办高教之　荒育复合高级人才－－西安翻译培训学院纪实 |
| 862 | 1998.11.02 | “知识经济经典汉译丛书”出版 |
| 863 | 1998.11.07 | 我为远山正瑛先生当翻译 |
| 864 | 1998.11.09 | 邓小平著作已译成七种少数民族文字 |
| 865 | 1998.11.10 | 突出听说读写译　大学公共英语教改初见成效 |
| 866 | 1998.12.04 | 面向 21 世纪国际翻译研讨会举行 |
| 867 | 1998.12.08 | 我国翻译工作者超过 50 万 |
| 868 | 1998.12.29 | 《达尔文进化论全集》中译本出齐 |
| 869 | 1999.01.06 | 作家翻译家叶君健逝世 |
| 870 | 1999.01.29 | 《纲鉴易知录》注释今译本评介 |
| 871 | 1999.02.12 | 著名作家翻译家萧乾逝世 |
| 872 | 1999.03.09 | 译文本供不应求 |
| 873 | 1999.04.21 | 徐放新诗翻译《唐诗三百首》出版 |
| 874 | 1999.05.18 | 《西域考古图记》有了汉译本 |
| 875 | 1999.06.15 | 《历代诗词曲千首精译》 |
| 876 | 1999.06.18 | 古籍今译管见 |
| 877 | 1999.06.18 | 古书今译现状堪忧 |
| 878 | 1999.07.16 | 神笔译名篇－－评《冰心译文集》 |
| 879 | 1999.07.24 | 翻译软件重新定位 |
| 880 | 1999.09.07 | 真理的火种唤醒民众－－《播火者译丛》序 |
| 881 | 1999.09.29 | 《经济学》第十六版中译本出版 |
| 882 | 1999.10.10 | 最早翻译毛选的日本人 |
| 883 | 1999.11.05 | 拉宾夫人回忆录中译本出版 |
| 884 | 2000.01.03 | 《马千云金书汉译古兰经》面世 |
| 885 | 2000.01.07 | 老外游漓江　翻译用不着 |

| | | |
|---|---|---|
| 886 | 2000.02.13 | 萧乾（1910－1999）杰出的作家、记者和翻译家 |
| 887 | 2000.03.05 | 代表为代表当翻译 |
| 888 | 2000.03.08 | 电文译错投递延误　电信邮政难辞其咎 |
| 889 | 2000.03.25 | 译作九成不合格有感 |
| 890 | 2000.04.08 | 《李有才板话》日译本 |
| 891 | 2000.04.22 | 苍白生硬的翻译腔 |
| 892 | 2000.05.11 | 《翻译学》 |
| 893 | 2000.06.10 | 翻译大家的风范－－悼念戈宝权 |
| 894 | 2000.06.15 | 金乡县：农民竞聘外语翻译 |
| 895 | 2000.07.10 | 《高校经典教材译丛·社会学》出版 |
| 896 | 2000.07.18 | 智洋网构建网上翻译中心 |
| 897 | 2000.08.04 | 应重视翻译工作和人才培养 |
| 898 | 2000.08.06 | 希望，在这里孕育－－西安翻译学院发展纪实 |
| 899 | 2000.09.02 | 世界古代文明的新认识－－“外国考古文化名著译丛”评介 |
| 900 | 2000.09.05 | “西译杯”奥运好新闻评选将举办 |
| 901 | 2000.10.07 | “休闲研究译丛”简介 |
| 902 | 2000.10.14 | “西译杯”奥运好新闻评选开始征稿 |
| 903 | 2000.10.26 | 西译杯奥运好新闻评选揭晓 |
| 904 | 2000.11.30 | WTO 法律文本最新中译本出版 |
| 905 | 2000.12.06 | 著名诗人、翻译家卞之琳逝世 |
| 906 | 2000.12.09 | 莎剧中译本的推陈出新－－兼议译林版《莎士比亚全集》 |
| 907 | 2000.12.18 | 中文进入国际语音互译大家庭 |
| 908 | 2000.12.21 | 全国新闻界网球邀请赛将开幕　《祖国的光荣　人民的骄傲》出版　申奥翻译者日活动在北京启动 |
| 909 | 2001.01.13 | 中国资深翻译家表彰大会在京举行钱其琛等为五十位翻译家颁发证书 |
| 910 | 2001.01.16 | 成功实现互联网页中英文互译双语浏览器突破网上语言瓶颈 |
| 911 | 2001.01.24 | 美国开发出可佩戴式翻译机 |
| 912 | 2001.02.09 | 语音翻译技术获突破 |
| 913 | 2001.03.09 | 少数民族语言同声传译 |
| 914 | 2001.03.31 | 轻松跨越网上语言障碍　网络翻译研究获重大突破 |
| 915 | 2001.04.13 | 巴金等人作品出版权著作权被侵案结案　14 位著（译）者和人民文学出版社胜诉 |
| 916 | 2001.04.14 | “译林少儿文库”奉献给读者 |
| 917 | 2001.05.14 | “商务通”翻译出错学校变成“军火库” |
| 918 | 2001.05.24 | 中国欧盟合作培养高级译员 |

| | | |
|---|---|---|
| 919 | 2001.07.02 | 山村传圣火——陈望道和《共产党宣言》第一个中译本的诞生 |
| 920 | 2001.07.05 | 课程设置与市场需求对接　大连翻译学院为社会精心育才 |
| 921 | 2001.07.14 | 捧出新编谢后人——读《骈体语译文心雕龙》 |
| 922 | 2001.07.21 | 中文译著《网》出版 |
| 923 | 2001.08.21 | 德推出同步口语翻译系统 |
| 924 | 2001.09.05 | 金陵翻译社靠“三强化”发展 |
| 925 | 2001.12.08 | 了解西方学术思想的一扇窗口——《当代西方主流学术名著译丛》简评 |
| 926 | 2002.01.17 | “美国法律文库”首批汉译本面世 |
| 927 | 2002.02.06 | 中美合作开发高性能编译系统 |
| 928 | 2002.03.06 | 民族语翻译工作展开 |
| 929 | 2002.04.11 | 《魔戒》频遭盗版　译林出版社悬赏十万堵源头 |
| 930 | 2002.04.21 | 厦门规范外语译文社会用字 |
| 931 | 2002.07.04 | 《公法名著译丛》出版 |
| 932 | 2002.07.16 | 培养实用型人才——西安翻译学院办学的启示 |
| 933 | 2002.07.27 | 萧乾和他的翻译 |
| 934 | 2002.11.11 | 延边干部进村翻译解说十六大报告 |
| 935 | 2002.12.26 | 著名翻译家蒋路逝世 |
| 936 | 2003.01.05 | 《逻辑学》新译本座谈会举行 |
| 937 | 2003.01.10 | 《马克思主义研究译丛》 |
| 938 | 2003.01.14 | 翠华山麓桃李芬芳——记西安翻译学院 |
| 939 | 2003.03.14 | “贴身”翻译 |
| 940 | 2003.04.08 | 文学翻译及两个比喻 |
| 941 | 2003.04.08 | 文学与翻译 |
| 942 | 2003.06.01 | 昆德拉作品新译本问世 |
| 943 | 2003.07.10 | 翻译资格认证走向标准化 |
| 944 | 2003.08.07 | “抢手生”是怎样培养的——访西安翻译学院院长丁祖诒 |
| 945 | 2003.09.05 | 北语启用中日同声传译教室 |
| 946 | 2003.09.29 | 350 多名专家学者历时 10 年编译　《诺贝尔奖讲演全集》出版 |
| 947 | 2003.10.15 | 《红楼梦》已被译成二十七种文字 |
| 948 | 2003.10.25 | 刘云山在中央编译局成立 50 周年座谈会上强调加强马克思主义基础理论研究　更好地为改革开放和现代化建设服务 |
| 949 | 2003.11.13 | 成仿吾与新译《共产党宣言》 |
| 950 | 2003.11.20 | 在马克思主义中国化进程中开创编译工作新局面 |
| 951 | 2003.11.21 | 译制片配音不能丢了个性 |

| | | |
|---|---|---|
| 952 | 2003.12.28 | 磨砺十年成一剑——读《文心雕龙新注新译》述怀 |
| 953 | 2004.01.16 | 《科学发现的揭密》有了中译本 |
| 954 | 2004.03.03 | 上海译文出版社词典被盗用案审结 |
| 955 | 2004.03.10 | 辛苦的翻译 |
| 956 | 2004.03.17 | 重金求译“东方天书” |
| 957 | 2004.03.21 | 瞻其华美——写在康德“三大批判”中文新译本出版暨康德逝世二百周年之际 |
| 958 | 2004.04.04 | “全国翻译专业资格考试”指定用书 |
| 959 | 2004.05.25 | 三十余年青灯黄卷 六百万言译著出版 罗希文：全文英译《本草纲目》第一人 |
| 960 | 2004.06.01 | 讲述安徒生的童话人生——访翻译家林桦 |
| 961 | 2004.08.22 | 我为小平同志当翻译 |
| 962 | 2004.08.26 | 忙碌的中文翻译 |
| 963 | 2004.08.30 | 乌鲁木齐史诗《江格尔》汉文全译本出版 |
| 964 | 2004.09.24 | 马克思主义经典著作编译和研究工作的新进展 |
| 965 | 2004.09.25 | 重译与合译中的利弊 |
| 966 | 2004.09.25 | 文学翻译大家谈 |
| 967 | 2004.10.19 | 外行谈译作 |
| 968 | 2004.11.05 | 我军军事翻译工作成果显著 |
| 969 | 2004.11.06 | 刘云山在参观中国翻译成就展时指出 支持和促进中国翻译事业的发展 为我国经济建设和改革开放服务 |
| 970 | 2004.11.09 | 我国翻译业 大而不强 差错堪忧　总体水平不高，粗制滥造之作仍很多 外文翻译差错“俯拾皆是”首因是高水平人才匮乏 |
| 971 | 2004.11.09 | 翻译出版物要去俗存雅 |
| 972 | 2004.11.12 | 难耐寂寞 后继乏人 出版短视　文学翻译现状亟待改观 |
| 973 | 2004.11.16 | 翻译文学呼唤批评 |
| 974 | 2004.11.17 | 《二十四史全译》赠送国家图书馆 |
| 975 | 2004.12.07 | 翻译的尴尬和委屈 |
| 976 | 2004.12.12 | 诗文并茂 译著成家——读《李霁野文集》 |
| 977 | 2004.12.21 | 马尼拉：中文翻译现机场（图片） |
| 978 | 2004.12.21 | 译者职业像演员 |
| 979 | 2005.01.06 | 二〇〇四年诺贝尔文学奖得主代表作《钢琴教师》中译本出版 |
| 980 | 2005.01.11 | 学会破译假话 |
| 981 | 2005.01.18 | 鲁迅文学奖优秀翻译奖缺席，翻译竞赛一等奖空缺，在全民学外语的今天——我国文学翻译后继乏人 |
| 982 | 2005.01.20 | 翻译是一门手工艺 |

| | | |
|---|---|---|
| 983 | 2005.01.28 | 以高水平的翻译推进中西哲学交流——“中西哲学交流中的翻译问题学术研讨会”述要 |
| 984 | 2005.02.04 | 文学翻译为何青黄不接 |
| 985 | 2005.02.25 | 贵州破译四卷古老水族水书 |
| 986 | 2005.04.11 | 郑州 聋哑人投票 手语翻译忙（图片） |
| 987 | 2005.04.29 | 文学翻译缺失多 |
| 988 | 2005.05.23 | 翻译出版业：浮华背后的忧思 |
| 989 | 2005.06.09 | 9 月 1 日起 翻译好不好 “标准”说了算 |
| 990 | 2005.06.26 | 斯特林堡，伟大而神秘——《斯特林堡文集》中文版译后谈 |
| 991 | 2005.07.05 | 当年曹雪芹在悼红轩中披阅十载，增删 5 次，完成了一部《红楼梦》。200 多年后，有一位学者用 27 年工夫将《红楼梦》译成法文，这位学者就是著名法籍华裔翻译家李治华 孜孜红楼求梦人 |
| 992 | 2005.07.13 | 它是水族的百科全书，是解译夏商文化的活化石——水书：捡拾文明的记忆 |
| 993 | 2005.07.22 | 音像制品出口亟待突破“瓶颈” 翻译、著作权、营销网络、市场竞争出现严重问题 |
| 994 | 2005.08.04 | 中国赢得第十八届世界翻译大会主办权 |
| 995 | 2005.08.15 | 同是小时工，报酬相差数百倍。同声传译、婚庆服务工资高，家政服务报酬低 宁波：小时工有了工资指导价 |
| 996 | 2005.09.01 | 《大中华文库》汉英对照蔚为大观 中国文化典籍有了权威译本 |
| 997 | 2005.09.20 | 为翻译和研究安徒生的作品燃尽生命烛火——林桦：穿行于童话森林 |
| 998 | 2005.09.22 | 一把打开欧洲文化的钥匙——评杨广胜编译的《希腊神话》 |
| 999 | 2005.10.07 | 《企业理论译丛》 |
| 1000 | 2005.10.10 | 架起沟通桥梁——访联合国中文口译专家朱敬文 |
| 1001 | 2005.10.27 | 我国图书进出口比约为九比一 出版物“走出去”将获更多政策扶持 推荐代表中国文化特色书目 出版中国图书可补贴翻译费 图书出口可享退税优惠政策 出国展销图书将获资金支持 |
| 1002 | 2005.11.30 | 手机与翻译机可望合一 |
| 1003 | 2006.01.15 | 宁夏 两千农村青年当上“翻译官” |
| 1004 | 2006.02.28 | 百部精品“走出去” 当代文学“译”出国门 |
| 1005 | 2006.03.04 | 藏语“翻译” |
| 1006 | 2006.03.26 | 完整再现原著风貌——《洛丽塔》新译本简介 |
| 1007 | 2006.04.09 | 中意学术文化交流的新成果 “克罗齐史学名著译丛”出版 |

| | | |
|---|---|---|
| 1008 | 2006.04.09 | 穆旦译文诗文　首次系统出版 |
| 1009 | 2006.04.13 | 乘坐沪上地铁诵吟英伦名诗　上海打造“文化地铁”英译唐诗在伦敦地铁亦被争相阅读 |
| 1010 | 2006.06.16 | 普及经典著作也应与时俱进－－《资本论》（缩译彩图本）简评 |
| 1011 | 2006.08.20 | 一个老翻译的感悟 |
| 1012 | 2006.11.05 | 出版界人士研讨“译林现象” |
| 1013 | 2006.11.05 | 在中非合作论坛北京峰会开幕式上的讲话（译文）（2006 年 11 月 4 日） |
| 1014 | 2006.11.05 | 在中非合作论坛北京峰会开幕式上的讲话（译文）（2006 年 11 月 4 日）（续） |
| 1015 | 2006.11.05 | 《贞观政要》新译本面世 |
| 1016 | 2006.11.13 | 我国将向世界译介百部当代文学精品　首批文学力作将“远游”三国 |
| 1017 | 2006.12.12 | 此龙非彼龙，专家建议中国龙更改英译名－－该如何称呼你，中国龙 |
| 1018 | 2006.12.22 | 《协商民主译丛》 |
| 1019 | 2007.01.05 | 北京奥运会翻译服务供应商确定 |
| 1020 | 2007.01.07 | 走出编译状态－－“全国外国美术研究与教学发展战略研讨会”综述 |
| 1021 | 2007.01.14 | 翻译文学的版本 |
| 1022 | 2007.01.28 | 《小世界》出新译本 |
| 1023 | 2007.02.01 | 改变“文化交流单行道”格局 百部当代文学精品将“走出去”　首批力作将译介到俄罗斯 |
| 1024 | 2007.03.18 | 从中译本说“《魔山》热” |
| 1025 | 2007.03.28 | 俄罗斯的两次儒学译介 |
| 1026 | 2007.04.07 | 传播真理　业绩永存－－沉痛悼念马列经典著作翻译家毛岸青同志 |
| 1027 | 2007.04.09 | 中医药术语将有统一“洋名”　已完成５７００多个英译词条 |
| 1028 | 2007.06.11 | 中国文学首次在巴译成乌尔都语 |
| 1029 | 2007.07.02 | 《莫斯科郊外的晚上》中文译配者首访俄罗斯 |
| 1030 | 2007.07.06 | 精心选译人民日报的文章　帮助读者了解真实的中国　《必读：有趣的中国》面世 |
| 1031 | 2007.08.22 | 21 日落幕的书展上，两套特殊的丛书受到青睐－－上海图书大步“走出去”　输出版权种数约占全国的 1/5　翻译人才不足是目前最大问题 |
| 1032 | 2007.08.23 | 全球 3 万多名志愿者翻译　中华老字号已有 7 国“洋名” |
| 1033 | 2007.12.23 | 《没有我们的世界》出中译本 |
| 1034 | 2008.01.01 | 统一 1500 多个名词翻译　中医术语有了国际标准 |

| | | |
|---|---|---|
| 1035 | 2008.01.16 | “gobelieve”成了“狗不理”的英译名　老字号，怎么给你取“洋名” |
| 1036 | 2008.03.02 | 世界翻译大会将在沪举行 |
| 1037 | 2008.04.10 | 中国对外翻译出版公司成立三十五周年 |
| 1038 | 2008.04.19 | 著名翻译家沙博理在《中国日报》撰文——不能遗忘的历史 |
| 1039 | 2008.06.19 | 《中国文化读本》北京首发　奥运前将出多种文字译本 |
| 1040 | 2008.07.10 | 西班牙非常愿意同中国开展体育交流，专门从中国大学生中培训了 30 名翻译，他们不仅是北京奥运会上的翻译，同时也是西中两国人民友谊的桥梁。在北京奥运会上，西班牙运动员会——感觉就像在家里一样 |
| 1041 | 2008.07.28 | 我国翻译机器人达国际领先水平 |
| 1042 | 2008.08.05 | 55 年来首次在亚洲国家举办　世界翻译大会在上海开幕 |
| 1043 | 2008.09.11 | 日本老人抱病译《人虫儿》 |
| 1044 | 2008.09.24 | 台湾有关方面决定将中文译音政策由采用“通用拼音”改为“汉语拼音”　切中肯綮　务实之举 |
| 1045 | 2008.11.17 | 手语翻译助聋哑人打电话 |
| 1046 | 2008.11.17 | 商务将出版全套“汉译名著”丛书 |
| 1047 | 2008.12.23 | 有感于吴仁宝六换翻译 |
| 1048 | 2009.01.04 | 傅雷译著手稿全部捐赠国家图书馆 |
| 1049 | 2009.01.10 | 鲁迅文学院举办少数民族文学翻译家研讨班 |
| 1050 | 2009.03.30 | 中国文化著作翻译出版工程实施 |
| 1051 | 2009.04.02 | 《剑桥世界古代史》翻译工程启动 |
| 1052 | 2009.04.06 | “简化”与“今译”之辨 |
| 1053 | 2009.07.18 | 《鲁迅著译编年全集》出版 |
| 1054 | 2009.07.28 | “五经”将被译成八种外文 |
| 1055 | 2009.08.04 | 新加坡研发出网络语音翻译系统 |
| 1056 | 2009.09.08 | 《译林》迎来“30 岁” |
| 1057 | 2009.09.25 | 汉译世界学术名著丛书珍藏版出版座谈会举行 |
| 1058 | 2009.09.25 | 文化“走出去”如何迈过翻译这道坎 |
| 1059 | 2009.10.13 | 蒙古国出版“中文典籍译丛” |
| 1060 | 2009.11.14 | 306 名资深翻译家受表彰 |
| 1061 | 2009.11.17 | 翻译是桥梁也可能是屏障（新语·如何让世界了解中国文化③） |
| 1062 | 2009.11.25 | 中国翻译界期待“后大师时代” |
| 1063 | 2009.11.25 | 他几乎“翻译了整个中国”（人物） |

| | | |
|---|---|---|
| 1064 | 2009.11.30 | 著名翻译家杨宪益遗体告别仪式在京举行 |
| 1065 | 2009.11.30 | 翻译这座桥还牢吗（文化观察·谁来向世界介绍中国①） |
| 1066 | 2009.12.01 | 文学翻译缘何沦为“零首选”（文化观察·谁来向世界介绍中国②） |
| 1067 | 2009.12.07 | 首届傅雷翻译出版奖揭晓 |
| 1068 | 2009.12.18 | 《公共哲学》译丛出版（课题追踪） |
| 1069 | 2009.12.28 | 编译两部文集的几点体会 |
| 1070 | 2009.12.28 | 关于两部文集编译工作的汇报 |
| 1071 | 2010.04.16 | 西宁各医院急需藏语翻译（全力抗震救灾救援受灾群众） |
| 1072 | 2010.04.19 | 藏汉语翻译志愿者值守“亲情电话”（第一线） |
| 1073 | 2010.08.04 | 游客协助、秩序引导、语言翻译……———一名“小白菜”日均服务数百人 |
| 1074 | 2010.08.14 | “错译”营直人谈话引争议 |
| 1075 | 2010.08.18 | 新疆民族语文翻译业务骨干研修班举办 |
| 1076 | 2010.08.31 | 南京铁路公安处刘勇三月两赴上海支援世博——站台上走来“警察翻译”（世博画廊） |
| 1077 | 2010.09.03 | 月球地名首次有了标准中文译名 |
| 1078 | 2010.12.03 | “带着理想去翻译” |
| 1079 | 2010.12.10 | 中央编译局正根据历史考证版编译《马克思恩格斯全集》中文第 2 版 |
| 1080 | 2011.05.23 | 中外学者齐聚京城共同探讨翻译教育 |
| 1081 | 2011.06.24 | 中央编译局最近收藏的马克思的两封信 |
| 1082 | 2011.11.21 | 百年汉译名著丛书首获国家基金资助 |
| 1083 | 2012.06.20 | 中国翻译职业大会召开 |
| 1084 | 2012.07.08 | 黄裳的翻译接力（跋与藏） |
| 1085 | 2012.10.05 | 可用不可滥，翻译要到位（文论天地） |
| 1086 | 2012.11.11 | “同声翻译”传精神 |
| 1087 | 2012.11.15 | 计算机翻译技不如人（科技大观） |
| 1088 | 2012.12.24 | 指尖之舞沟通无声世界（探访熟悉的陌生人·走进新职业群体·手语翻译师） |
| 1089 | 2013.03.16 | 192 位民族语文翻译员服务两会 |
| 1090 | 2013.05.23 | 做起义务“翻译官”（身边的感动·菊美多吉故事汇④） |

| | | |
|---|---|---|
| 1091 | 2013.07.25 | 新版《马克思恩格斯选集》和《列宁选集》的编译思路与版本特色 |
| 1092 | 2013.11.16 | 文法结合探路中国小说外译 |
| 1093 | 2014.01.07 | 从被动接受译制任务到主动做大声音产业 |
| 1094 | 2014.01.10 | ７月１５日起公共服务领域外文要按规范译写 |
| 1095 | 2014.01.27 | 国家多语种影视译制基地挂牌 |
| 1096 | 2014.04.08 | 诗体《莎士比亚全集》译本面世 |
| 1097 | 2014.04.25 | “零翻译”何以大行其道（解码） |
| 1098 | 2014.06.06 | 《习近平关于实现中华民族伟大复兴的中国梦论述摘编》一书多语种版翻译出版 |
| 1099 | 2014.07.29 | 新媒体时代，什么值得翻译（文艺观察·关注翻译文化?） |
| 1100 | 2014.08.04 | 中国翻译家首次问鼎国际译联大奖 |
| 1101 | 2014.08.05 | 填补非通用语种外译空白（文艺观察·关注翻译文化?） |
| 1102 | 2014.08.05 | 跟翻译“倔”了一辈子（人物） |
| 1103 | 2014.08.12 | 辩证认识翻译中的“误读”（文艺观察·关注翻译文化?） |
| 1104 | 2014.08.20 | 汉学家文学翻译国际研讨会在京举行 |
| 1105 | 2014.08.24 | 翻译家要做“信徒” |
| 1106 | 2014.09.17 | 中央编译局举办“邓小平著作翻译成果展” |
| 1107 | 2014.11.02 | 译莫言作品看中国文学 |
| 1108 | 2014.11.03 | 国际汉学翻译家大会举行 |
| 1109 | 2015.02.24 | 译海寻梦青春常驻 |
| 1110 | 2015.03.04 | 从联合国翻译到武警战士（行进中国·精彩故事·新春走基层） |
| 1111 | 2015.03.19 | 翻译，怎样向世界讲好中国故事（深聚焦） |
| 1112 | 2015.04.22 | 中译外工作量高于外译中 |
| 1113 | 2015.04.23 | 党的十八届四中全会重要文件以多种外语翻译出版 |
| 1114 | 2015.05.07 | 64 载编译经典信仰铸就崇高（学者风采） |
| 1115 | 2015.05.11 | 中译外升温不意外 |
| 1116 | 2015.05.25 | 进口大片，为何屡陷“翻译门” |
| 1117 | 2015.06.27 | 文学互译增进了解（共创中俄媒体发展新未来） |
| 1118 | 2015.08.26 | 20 位国外作家翻译家出版家获殊荣 |
| 1119 | 2015.09.01 | 中外出版翻译恳谈会举行 |
| 1120 | 2015.10.28 | 勿让事实在“翻译”中跌落（快评） |

| | | |
|---|---|---|
| 1121 | 2015.12.12 | 首届“品读中国”文学翻译奖在莫斯科颁奖 |
| 1122 | 2016.01.11 | 当代精品直通世界文学翻译沟通心灵 |
| 1123 | 2016.06.11 | 注译《古文观止》的晶如先生 |
| 1124 | 2016.06.12 | 中外影视译制合作专家座谈会举行 |
| 1125 | 2016.06.20 | 《共产党宣言》首译本保存记（光辉历程） |
| 1126 | 2016.09.19 | 省级行政机关红头文件要将官话翻译成大白话 |
| 1127 | 2016.10.09 | 俄文全译本《牡丹亭》面世 |
| 1128 | 2016.11.05 | 《中国经典阿拉伯语译丛》首发式在京举行 |
| 1129 | 2016.11.15 | 誓死守护《共产党宣言》中文首译本 |
| 1130 | 2016.11.23 | “阿拉伯翻译家与汉学家联谊会”在开罗成立 |
| 1131 | 2016.11.27 | 隐形的翻译家（五洲茶亭） |
| 1132 | 2017.01.08 | 译事难，译诗更难（五洲茶亭） |
| 1133 | 2017.01.23 | 服务台装软件帮老外翻译加油站送姜汤暖回家之路（2017 春运故事·第六回） |
| 1134 | 2017.04.14 | 标识可做本地化“翻译”（大家谈·务实推进垃圾分类②） |
| 1135 | 2017.05.07 | 译者要成为作者的捍卫者 |
| 1136 | 2017.05.23 | 为法官断案当“翻译”（深阅读） |
| 1137 | 2017.06.26 | 公共服务领域英文译写规范国标发布（资讯速递） |
| 1138 | 2017.07.04 | 翻译出版如何与时俱进（青年文化论坛） |
| 1139 | 2017.07.14 | 与译作一同成长 |
| 1140 | 2017.08.01 | 中国四大古典文学名著全部译成马来文出版发行 |
| 1141 | 2017.08.09 | 文化译介助推中华文化走出去（大家手笔） |
| 1142 | 2017.08.09 | 讲好中国故事需创新对外翻译（新知新觉） |
| 1143 | 2017.09.19 | 中乌签署中国影视剧译制播出合作协议 |
| 1144 | 2017.11.14 | “我怎么成了布罗代尔的中文译者” |
| 1145 | 2017.11.23 | 文化“走出去”呼唤翻译精品意识（深观察） |
| 1146 | 2017.11.28 | 共同翻译出版《习近平谈治国理政》第二卷 |
| 1147 | 2018.02.28 | 智能翻译真神奇（2018 春运一线） |
| 1148 | 2018.03.08 | 直播用上“巧翻译”音频有了“录入员” |
| 1149 | 2018.04.19 | 24 国电影人研修影视译制 |
| 1150 | 2018.05.01 | 耿 N：敦厚长者译坛骁将（书人书事） |
| 1151 | 2018.05.31 | 让翻译中国文学的传统代代相传 |
| 1152 | 2018.08.23 | 中外文学出版翻译合作研修班开课 |
| 1153 | 2018.08.30 | 《新华字典》“汉格版”翻译出版工作启动 |
| 1154 | 2018.11.29 | 《格萨尔》初译任务完成 |
| 1155 | 2018.11.30 | 壮文智能翻译软件发布 |
| 1156 | 2018.12.06 | 2018 中国翻译协会年会在京举行（微阅读） |

| | | |
|---|---|---|
| 1157 | 2019.01.08 | 二十四史全译 |
| 1158 | 2019.01.27 | 捷克汉学家克拉尔的研究和翻译深深影响了人们对中国文化的认知　实至名归的殊荣 |
| 1159 | 2019.02.15 | 蒙古国立大学孔子学院蒙方院长其米德策耶翻译中国经典、促进文化交流获得多项奖励——<br>“这是中国实施改革开放给我带来的机遇” |
| 1160 | 2019.02.22 | 翻译就是追求两种语言“双赢”（名师谈艺） |
| 1161 | 2019.02.22 | 北京大学教授、翻译大家仲跻昆——<br>六十余载，只为架设中阿文化之桥（治学） |
| 1162 | 2019.02.26 | 二十四史全译 |
| 1163 | 2019.03.26 | 九旬德语翻译家、翻译文化终身成就奖获得者宋书声——<br>毕生坚守 尤显厚重（讲述·一辈子一件事） |
| 1164 | 2019.05.14 | “汉译名著”：新时代，新使命 |
| 1165 | 2019.05.21 | 欧洲汉学：探寻中国发展的历史和文化因素（国际视野） |
| 1166 | 2019.05.22 | 图书互译 我们更懂彼此（讲好亚洲故事） |
| 1167 | 2019.06.21 | 翻译和研究相得益彰（名师谈艺） |
| 1168 | 2019.07.02 | 译笔搭桥 沟通世界（名师谈艺） |
| 1169 | 2019.07.14 | 《论语》译本谈 |
| 1170 | 2019.08.11 | 好的翻译还需要“创造”（五洲茶亭） |
| 1171 | 2019.08.13 | 近年来，黎巴嫩“数字未来”出版公司已翻译出版二百多种中国图书<br>“为中国优秀图书的传播插上翅膀” |
| 1172 | 2019.08.20 | 让文学译介更精彩（暖闻热评·择一事 终一生（14）） |
| 1173 | 2019.08.21 | 第十三届中华图书特殊贡献奖颁发<br>15 位来自国外的作家、翻译家、出版家获奖 |
| 1174 | 2019.09.03 | “中俄经典与现代文学作品互译出版项目”已翻译出版作品 94 部 |
| 1175 | 2020.04.17 | 翻译没有捷径（名师谈艺） |
| 1176 | 2020.04.29 | 志愿有我，以“译”战疫（暖闻热评） |
| 1177 | 2020.06.14 | 翻译的力量（五洲茶亭） |
| 1178 | 2020.06.21 | 钟情中国当代文学译介的雅拉·密斯里——<br>近距离感受当下中国脉动(海客谈神州) |
| 1179 | 2020.08.03 | “真理的味道非常甜”（人民论坛）<br>——纪念《共产党宣言》中文首译本出版一百周年① |

| | | |
|---|---|---|
| 1180 | 2020.08.04 | “用信仰之力开创美好未来”（人民论坛）<br>——纪念《共产党宣言》中文首译本出版一百周年② |
| 1181 | 2020.08.05 | “不忘初心，方得始终”（人民论坛）<br>——纪念《共产党宣言》中文首译本出版一百周年③ |
| 1182 | 2020.08.06 | “我们的道路必将越走越宽广”（人民论坛）<br>——纪念《共产党宣言》中文首译本出版一百周年④ |
| 1183 | 2020.08.07 | “为人类作出新的更大的贡献”（人民论坛）<br>——纪念《共产党宣言》中文首译本出版一百周年⑤ |
| 1184 | 2020.08.09 | “翻译的使命是沟通和交流，为读者服务”（海客谈神州） |
| 1185 | 2020.08.10 | “只有中国共产党才能领导中国”（人民论坛）<br>——纪念《共产党宣言》中文首译本出版一百周年⑥ |
| 1186 | 2020.08.16 | 希腊翻译家索提里斯·查理克亚斯——<br>“帮助希腊读者了解真正的中国”（海客谈神州） |
| 1187 | 2020.10.28 | 老中青三代编译人，坚守马列经典编译阵地——<br>他们让真理穿越时空（讲述·一辈子一件事） |
| 1188 | 2020.11.04 | 多部脱贫攻坚报告文学将翻译出版 |
| 1189 | 2020.12.20 | 首届“俄中文学外交翻译奖”颁奖典礼在京举行 |
| 1190 | 2021.01.06 | 中国巴基斯坦签署关于经典著作互译出版的备忘录 |
| 1191 | 2021.01.19 | 中韩签署关于经典著作互译出版的备忘录 |
| 1192 | 2021.03.03 | 17 国出版机构与我签署备忘录<br>共同翻译出版《习近平谈治国理政》第三卷 |
| 1193 | 2021.03.17 | 中伊签署关于经典著作互译出版的备忘录<br>以书为媒　推动中伊文明交流互鉴 |
| 1194 | 2021.04.06 | 不断加强中译外能力建设（观点） |
| 1195 | 2021.04.13 | 深耕文学翻译 增进文化交流（创造性转化创新性发展纵横谈） |
| 1196 | 2021.04.27 | 中老签署关于经典著作互译出版的备忘录<br>为构建中老命运共同体注入人文动力 |
| 1197 | 2021.05.10 | 共同维护人类文化多样性<br>——访埃及汉学家、翻译家法尔贾尼 |
| 1198 | 2021.08.22 | “中国哲学给我前所未有触动”（翻译家说） |
| 1199 | 2021.10.10 | “文学翻译不仅仅是一份职业”（翻译家说） |
| 1200 | 2021.10.20 | 是乐趣也是荣耀（翻译家说） |

| | | |
|---|---|---|
| 1201 | 2021.11.14 | 在诗意中感知中国文化（翻译家说） |
| 1202 | 2021.11.21 | 好翻译要上“社会大学”（翻译家说） |
| 1203 | 2021.11.26 | 中亚签署关于经典著作互译出版的备忘录<br>开启两国人文交流互鉴新阶段 |
| 1204 | 2021.12.11 | 党的十九届六中全会主要文件外国语种和民族语种翻译文本出版发行 |
| 1205 | 2022.01.11 | 首部荷兰语全译本《红楼梦》问世 |
| 1206 | 2022.02.07 | 中吉签署关于经典著作互译出版的备忘录<br>开启两国人文交流互鉴新阶段 |
| 1207 | 2022.02.10 | 《格萨尔》藏译汉丛书出版发行 |
| 1208 | 2022.02.11 | 搭建沟通心灵的彩虹（译者·书） |
| 1209 | 2022.02.16 | 国产智能雪蜡车、智能机器人、便携式智能翻译设备纷纷亮相 “中国智造”闪耀北京冬奥（新视点） |
| 1210 | 2022.02.17 | 文学翻译：语感与美感之间（译者·书） |
| 1211 | 2022.03.20 | 让读者感受到文化的博大（译者·书） |
| 1212 | 2022.03.30 | 我译莫泊桑（译者·书） |
| 1213 | 2022.03.31 | 中国与阿塞拜疆签署关于经典著作互译出版的备忘 开启两国人文交流互鉴新阶段 |
| 1214 | 2022.04.06 | 文学花园中的“采蜜人”（译者·书） |
| 1215 | 2022.05.13 | 生生不息活水流（译者·书） |
| 1216 | 2022.06.26 | 中蒙签署经典著作互译出版备忘录<br>促进两国人文交流互鉴 |
| 1217 | 2022.07.05 | 我与《安徒生童话》（译者·书） |
| 1218 | 2022.07.08 | 天津理工大学鲸言创益团队研发手语实时翻译系统——让人工智能看懂手语（解码·走近新职业） |
| 1219 | 2022.08.02 | 破译古埃及象形文字 |
| 1220 | 2022.08.16 | 唯有友谊， 才是世界的珍宝（译者·书） |
| 1221 | 2022.09.27 | 著名翻译家、儿童文学作家任溶溶——“我一辈子就是为孩子们写书” |
| 1222 | 2022.11.01 | 中也签署经典著作互译出版备忘录 促进两国人文交流互鉴 |
| 1223 | 2022.11.16 | 飞架中泰的文学翻译之桥 |

| | | |
|---|---|---|
| 1224 | 2023.01.06 | “世界有如海洋，时代有如劲风”（译者・书）——我译《阿拜》 |
| 1225 | 2023.01.13 | 跨越时空的交谈（译者・书） |
| 1226 | 2023.02.10 | 文学互译架设中阿文明交流之桥 |
| 1227 | 2023.02.21 | 借鉴京剧唱词 翻译史诗名著（译者・书） |
| 1228 | 2023.03.15 | 中泰签署经典著作互译出版备忘录 促进两国人文交流互鉴 |
| 1229 | 2023.03.28 | 大江健三郎文学译事略记（译者・书） |
| 1230 | 2023.04.04 | 中国翻译协会年会在京召开 |
| 1231 | 2023.04.24 | 传递爱与光明（译者・书）——写在童话《小王子》出版八十周年之际 |
| 1232 | 2023.08.03 | 中约签署经典著作互译出版备忘录　促进两国人文交流互鉴 |
| 1233 | 2023.08.09 | 一片诗意的花园 一座友谊的桥梁（译者・书）——译马丁・瓦尔泽有感 |
| 1234 | 2023.08.29 | “不到园林，怎知春色如许”（译者・书）——从德译本《说园（典藏版）》说开去 |
| 1235 | 2023.09.06 | 中国文学国际传播论坛暨第六次汉学家文学翻译国际研讨会在南京开幕 |
| 1236 | 2023.09.11 | 京津冀翻译协会协同发展学术论坛举行 |
| 1237 | 2023.09.27 | 中尼签署经典著作互译出版备忘录 开启两国人文交流互鉴新阶段 |
| 1238 | 2023.10.03 | 中国和沙特文学、出版、翻译领域项目执行计划在利雅得签署 |
| 1239 | 2023.11.27 | 新时代对外话语创新高峰论坛暨第三十五届韩素音国际翻译大赛颁奖典礼在京举行 |
| 1240 | 2023.12.19 | 中越签署关于经典著作互译出版的备忘录 |
