# Supplementary material for: Contemporary Chinese newspaper discourse on translation: A mixed-methods approach to the People’s Daily (1949–2023)
Source: PLoS One. 2026 Mar 18;21(3):e0343447. doi: 10.1371/journal.pone.0343447 (PMC12998856; doi:10.1371/journal.pone.0343447)
Supplement: S1 File — S1 Appendix. Complete list of article entries. S2 Appendix. Complete list of article entries (English version). (ZIP) [file pone.0343447.s001.zip › Supporting Information/S2 Appendix. Complete list of article entries (English version).pdf]

No.	Date	Title (literal translation)
1	1949.06.11	Eliminating colonial habits and compiling Chinese industrial textbooks — Tsinghua Engineering College organizes committee to discuss draft submitted by Professor Liu Xianzhou
2	1949.07.24	Polish President Bierut's speech on Poland's Liberation Day — From a July 21 Polish News Agency dispatch
3	1949.07.26	U.S. imperialism in Taiwan — Condensed translation from the June 1 issue of *New Times*
4	1949.08.04	Polish translations of Lenin and Stalin's works to be published
5	1949.08.10	New York and the small town of Hoboken — Abridged translation from *The True Face of America*
6	1949.08.15	Our beloved Moscow — Translated from the February 5, 1949 issue of *Soviet Literary Gazette*
7	1949.10.24	Czechoslovak Communists compile CPPCC documents, with a special preface by Chairman Mao — New collection to help European peoples understand New China
8	1949.11.14	The Soviet Union — A powerful industrial and collective-farming country — Abridged translation from *Komsomolskaya Pravda*
9	1949.11.15	The Soviet Union — A powerful industrial and collective-farming country — Abridged translation from *Komsomolskaya Pravda*
10	1949.11.27	New Russian translation of *Selected Works of Lu Xun*
11	1949.12.03	Over 400 cadres at the Revolutionary University study Russian in their spare time, aiming for basic reading and translation ability within nine months
12	1950.01.08	Chairman Mao's classic warmly received by the Burmese people — Burmese translation of "On the People's Democratic Dictatorship" widely circulated
13	1950.01.10	Executive meeting of the All-China Sports Federation Preparatory Committee sets up Secretariat and four committees for drafting, research, publicity and translation, and National Games planning
14	1950.02.08	Some thoughts on translating *The Character of Moscow*
15	1950.03.15	On translating Western personal names and place names
16	1950.03.26	Treat translation work with a serious attitude
17	1950.03.26	The so-called "completely new translation" of *How the Steel Was Tempered* published by Chaofeng Publishing House
18	1950.04.09	Conscientiously improving translation work
19	1950.04.26	German people warmly commemorate Lenin's birthday — German translation of Lenin's collected works to be issued soon
20	1950.04.28	On the Chinese rendering of "Soviet Union": "Soviet Union," "Soviet Russia" and "Russia" must not be used interchangeably
21	1950.05.03	Catalogue of Chinese translations of works by Marx, Engels, Lenin and

		Stalin
22	1950.05.03	On standardizing translated terms
23	1950.05.21	Two translated poems
24	1950.05.23	Russian translation of our CPPCC Common Program published in the Soviet Union — Chinese poetry evening held in Moscow
25	1950.05.31	Criticism and self-criticism of Zhang's translation of *Ludwig Feuerbach and the End of Classical German Philosophy* — Two letters from Wang Ruoshui to this journal
26	1950.05.31	Some comments on the translator's preface and appendices in the Sanlian edition of *The Life of Beethoven*
27	1950.06.16	Strengthening links among translators — *Translation Bulletin* to be launched soon
28	1950.07.08	First issue of *Translation Bulletin* published
29	1950.07.12	We need to publish more good translations!
30	1950.08.14	Second issue of *Translation Bulletin* published
31	1950.09.06	The translation community should persist in criticism
32	1950.11.22	Fifth issue of *Translation Bulletin* available — scientific workers nationwide may request copies by mail with a form
33	1950.12.23	Volume one, issue six of *Translation Bulletin* available — translators may request copies by submitting a form
34	1950.12.23	New book on electrical technology compiled and translated by the Ministry of Fuel Industry published — Distributed by Xinhua and Sanlian bookstores
35	1951.01.14	Soviet edition of the translation of "On Practice" published
36	1951.03.04	Work report from film translation staff at the three studios of the Film Bureau
37	1951.03.28	World Peace Council issues revised translation of its declaration on the conclusion of a peace pact
38	1951.03.31	In the war of aggression against Korea, MacArthur openly uses Chiang clique personnel, requisitioning translators and air force officers in Taiwan to go to Korea
39	1951.04.11	On the building of translation theory
40	1951.05.27	Review of Zhang Zongbing's revised translation of *Principles and Methods*
41	1951.06.07	*Pravda* publishes a special article by Petrov introducing the Russian translation of Lu Xun's collected works, stressing that Lu Xun's writings are permeated with love for the people and hatred of oppressors
42	1951.06.14	Experience of trying out a newly compiled natural science textbook
43	1951.06.15	Thai journal *Literary News* publishes Thai translation of Chairman Mao's "On Practice"
44	1951.06.25	Two translations of *China in Fighting*
45	1951.07.22	Burmese people closely follow New China —"On Practice" and other classics translated into Burmese as Rangoon prepares to establish a China–

		Burma Friendship Association
46	1951.07.27	Quotations from translated books should indicate the Chinese edition cited
47	1951.08.06	Examples of impure and unhealthy translations
48	1951.08.12	Post-translation notes on *Gulliver's Travels*
49	1951.08.18	Both original works and translations should state the year and month of completion
50	1951.10.10	Dedicating our best achievements to the great motherland — Presenting the completed translation of *Selected Works of Michurin* to Chairman Mao
51	1951.10.25	Let everyone actively take part in reviewing Chinese translations of the works of Marx, Engels, Lenin and Stalin
52	1951.11.10	First batch of *Selected Works of Mao Tse-tung* completely sold out — Translations into minority languages now under way
53	1951.11.11	Explanatory leaflets should be distributed when screening translated films with complex content
54	1951.11.26	Translated text of the letter and recorded broadcast by captured U.S. soldier Bell
55	1951.11.29	General Administration of Press and Publication convenes the first National Translation Work Conference
56	1951.12.14	Chairman Mao's "On Practice" published in Korean translation
57	1952.01.19	Crude bureaucratic work style causes grave harm: two major cases of waste in the Ministry of Central Trade — "three tons" of anti-epidemic drugs miswritten as "three hundred tons," tying up over four billion yuan; "rice-tea bricks" mistranslated as "black-tea bricks," costing the state 2.2 billion yuan
58	1952.03.03	Reprint of Lu Xun's translation of Gogol's great work *Dead Souls*
59	1952.03.12	A reply concerning the vulgarization of translated film titles
60	1952.04.11	Xinjiang People's Publishing House issues large quantities of books in minority scripts — Translation and publication of *Selected Works of Mao Tse-tung* actively under way
61	1952.04.28	Compilers and translators of language readers should pay close attention to content
62	1952.05.14	Personal and place names in foreign history and geography textbooks should be translated in a unified way
63	1952.05.17	Russian translation of volume one of *Selected Works of Mao Tse-tung* published in the Soviet Union
64	1952.05.20	Soviet people enthusiastically buy the Russian edition of *Selected Works of Mao Tse-tung*
65	1952.05.28	Preface to the Russian translation of the historical play *Qu Yuan*
66	1952.05.30	Editorial Department of the People's Education Press accepts proposal to standardize translated terms in textbooks
67	1952.06.12	Soviet Union to publish a Russian translation of *Tongjian Gangmu*

68	1952.06.22	*Selected Works of Mao Tse-tung* warmly received in Japan — Czechoslovakia publishes translation of *Thirty Years of the Communist Party of China*
69	1952.07.03	China Film Distribution Corporation headquarters calls for stronger publicity and explanation work for foreign-language films
70	1952.08.18	Contents of the 1952 No. 7 issue of *Study Translation Series*
71	1952.09.09	Introducing *Study Translation Series*
72	1952.10.31	Comrade Stalin's great new work widely published in many countries — Chinese translation of *Economic Problems of Socialism in the USSR* to be issued soon
73	1952.11.07	Chinese translation of Stalin's speech and Malenkov's report scheduled for publication today
74	1952.11.19	Soviet books and periodicals warmly received by our people — Over 3,100 Chinese translations of Soviet works published nationwide in the past three years
75	1952.12.23	Xinhua News Agency issues correction on the abbreviated Chinese name of the Communist Party of the Soviet Union
76	1952.12.26	Ministry of Higher Education leads universities and related bodies in translating Soviet higher-education textbooks
77	1953.01.05	We are now able to translate Russian-language textbooks
78	1953.04.01	Correcting waste in the translation and publication of advanced Soviet agricultural science books
79	1953.04.19	Xinjiang People's Publishing House translates volume two of *Selected Works of Mao Tse-tung* into three minority languages
80	1953.04.22	New reprint of the Chinese translation of *Capital* to be released
81	1953.05.10	Korea publishes translation of volume one, first part of *Selected Works of Mao Tse-tung*
82	1953.05.17	Introducing recently published separate Chinese translations of Stalin's works
83	1953.05.27	Self-criticism over waste in the translation and publication of Soviet agricultural science books
84	1953.06.08	Administrative sessions of the Korean Armistice talks continue — Staff officers and interpreters from both sides hold administrative meetings
85	1953.08.04	Foreign-language schools improve teaching to train translation personnel
86	1953.08.26	On promoting the work of film translation into minority languages
87	1953.09.05	Soviet Union publishes volume three of the Russian edition of *Selected Works of Mao Tse-tung*
88	1953.09.12	Polish member of the Neutral Nations Supervisory Commission writes to Clark protesting U.S. seizure of a Polish interpreter from a neutral inspection team
89	1953.10.16	Paris *L'Humanité* reporter Bequet reports that U.S. observers and Chiang

		clique interpreters openly obstruct explanation work
90	1953.11.05	Disgraceful conduct of U.S. “representatives” and “interpreters” in the explanation tent
91	1954.03.19	Central Committee Bureau for the Compilation and Translation of the Works of Marx, Engels, Lenin and Stalin holds exhibition of Marxist-Leninist classics
92	1954.04.05	China to dub and release more than forty films from the Soviet Union and other countries this year
93	1954.04.16	Science and Education Film Studio to shoot and dub many science-education films this year
94	1954.04.22	Five films dubbed into Tibetan begin distribution in Tibetan areas
95	1954.05.06	Number of Soviet books translated and published in China growing rapidly
96	1954.05.17	Chinese translations of *The Divine Comedy* and *Collected Plays of Shakespeare* reprinted
97	1954.05.19	Chinese translation of volume nine of *Collected Works of Stalin* published
98	1954.06.13	First fascicle of the Chinese translation of Darwin’s *On the Origin of Species* published
99	1954.06.17	Draft Constitution translated into Mongolian, Tibetan, Uygur, Kazakh, Korean and other minority languages
100	1954.06.27	Oriental Department of the Soviet State Publishing House for Fiction and Art translates and publishes Chinese literary works
101	1954.08.16	Strengthening leadership in literary translation work
102	1954.08.16	The “not seeking thorough understanding” attitude in translation
103	1954.08.29	China Writers Association convenes national conference on literary translation
104	1954.08.29	Strive to develop literary translation and raise translation quality — abstract of a report delivered at the National Conference on Literary Translation Work on 19 August 1954
105	1954.08.29	On literary translation work
106	1954.09.08	A review and self-criticism of the translation of “For the Further Advancement of Soviet Literature”
107	1954.11.09	Northeast Film Studio has translated large numbers of Soviet films
108	1954.12.12	Chinese translation of the political economy textbook to be published around next April
109	1955.01.29	Chinese cultural delegation performs for overseas Chinese in Rangoon; Burmese Translators Association hosts a banquet
110	1955.02.28	Thirty-two Chinese translations of works by Marx, Engels, Lenin and Stalin to be issued this year
111	1955.03.24	Many important documents have been translated into Tibetan
112	1955.04.12	Chinese translations such as *Selected Works of Pollitt* published
113	1955.04.23	More Chinese translations of literary works from Asian countries

114	1955.07.06	Translation quality of science and technology books must be improved
115	1955.07.06	Two suggestions on translated terms
116	1955.08.30	Translate and publish more outstanding foreign works
117	1955.10.19	Introducing *Art and Construction in India* — Documentary produced by the Indian Documentary Film Studio and dubbed by the Central Newsreel and Documentary Film Studio
118	1955.11.07	Large numbers of Chinese translations of Soviet literary works published
119	1955.12.02	Reading the Chinese translation of *Selected Works of Pavlov*
120	1955.12.06	On standardizing Chinese language in translation work
121	1955.12.09	Watching the documentary *Songs of Peace in Japan* — Produced by the Japan Documentary Film Production Association and the Japan Central Music Association, dubbed by Changchun Film Studio
122	1955.12.25	Chinese translation of volume 12 of the *Complete Works of Stalin* published
123	1955.12.28	Chinese translation of volume 1 of the *Complete Works of Lenin* published
124	1956.01.12	Complete works of Marx, Engels, Lenin and Stalin to be fully translated into Chinese before 1960
125	1956.01.14	Volume two of the Chinese translation of *Selected Works of Marx and Engels* goes on sale
126	1956.03.09	Chinese translations of documents of the CPSU Congress published
127	1956.05.21	Committee on Compilation, Translation, and Publication of the Chinese Academy of Sciences established
128	1956.07.10	Selected translations from the *Book of Songs*
129	1956.07.13	Academy of Traditional Chinese Medicine compiles and translates classical works of traditional Chinese medicine
130	1956.07.17	Korean translation of *Capital* published
131	1956.07.19	Beijing translators hold forum on "translation standards"
132	1956.08.22	Nasser's *The Philosophy of the Revolution* translated into Chinese
133	1956.09.01	Japan publishes translated and annotated edition of *Manwen Laodang*
134	1956.09.02	Chinese translation *Selected Philosophical Writings of Diderot* published by Joint Publishing
135	1956.09.02	Chinese translation of La Mettrie's *Man a Machine* published by Joint Publishing
136	1956.09.05	Making rational use of translation personnel
137	1956.09.06	Chinese translation of Nehru's *The Discovery of India* published
138	1956.09.07	Committee of Compilation, Translation, and Publication of the Chinese Academy of Sciences discusses how to implement 'contention of all schools of thought'
139	1956.09.09	An abstruse translation
140	1956.09.16	Modern translation of the *Book of Songs*
141	1956.10.05	French scholar translates and annotates the India and Frankish history sections of Rashid's *Compendium of Chronicles*

142	1956.10.07	Polish readers embrace Chinese works — fifty translated titles reach a circulation of one million copies
143	1956.10.12	Ministry of Labor replies on the problem of surplus Russian translators
144	1956.10.21	On the issue of translated terms
145	1956.10.23	Soviet Academy of Sciences discusses production automation — meeting focuses on electronic computers, demonstrates machine translation, and introduces information theory
146	1956.10.26	Translated terms should be as unified as possible
147	1956.10.28	On Yan's translation
148	1956.11.02	Modern translation of Fan Zhen's "Treatise on the Annihilation of Spirit" published
149	1956.11.16	On reading *Classical Poems in Modern Translation*
150	1956.11.23	Introducing *Personality Psychology* — Soviet work by A. G. Fedorova et al., translated by Jia Ming et al., published by Science Press
151	1956.11.28	How to improve and develop translation work
152	1956.11.28	Welcoming modern translations of ancient philosophical essays
153	1956.12.03	Modern translation of the *Book of Songs*
154	1956.12.15	An international conference that needs no translation — impressions of the 41st World Esperanto Congress
155	1956.12.28	Reading "On Yan's Translation"
156	1957.01.03	"Atomic Medicine Translation Series" launched
157	1957.01.11	Hopes and demands of translators and foreign-language students
158	1957.01.25	Selected translations of Afghan folk poetry
159	1957.02.14	Our country to translate over 100 Soviet films
160	1957.02.16	Ancient Chinese translations of Vedic verses
161	1957.02.17	Soviet Union to publish Chinese classics ancient and modern — *Book of Songs*, *Dream of the Red Chamber* and others to appear in Russian for the first time
162	1957.02.26	Soviet Union vigorously develops cultural ties with other countries — five thousand cultural workers to visit abroad this year; now ranks first worldwide in publishing translated books
163	1957.02.27	Science Press expands its publishing scope — output of translated foreign scientific works begins to increase
164	1957.02.28	Classic works must be translated with seriousness — on problems in Cao's translation of *The Development of Capitalism in Russia*
165	1957.03.26	Paralyzed youth Jiang Younong perseveres in self-study for twelve years, writes and translates nearly one million characters of books
166	1957.04.02	Improving translation work
167	1957.04.13	Work assignments for translators rearranged
168	1957.04.23	Comrade-in-arms — diary excerpts of a Russian-language interpreter
169	1957.04.24	On early Chinese translations of Goethe's works

170	1957.05.25	Translator of *Capital* Wang Yanan joins the Party
171	1957.06.01	Translation and publishing
172	1957.06.17	Wei Lai — a successful translator of Chinese literature
173	1957.08.06	People's Health Publishing House compiles and translates *Selected Works of Sechenov*
174	1957.08.16	Many classic works in philosophy and social sciences to be translated into Chinese and published
175	1957.08.20	First issue of the "Literary Theory Translation Series" published
176	1957.09.02	Chinese translations of works by world cultural figures for 1957 released in succession
177	1957.09.21	Chinese translation of *The Origin and Basis of Human Inequality* published
178	1957.09.26	Publishing Chinese translations of famous works of Soviet literature
179	1957.10.15	Commemorating the 40th anniversary of the October Revolution: a collection of plays by Soviet writers was compiled and published
180	1957.10.15	*Literary Gazette* and *Yiwen* publish special issues
181	1957.10.15	Books in Miao script compiled and translated in western Hunan
182	1957.10.19	Large numbers of Soviet films dubbed into Chinese
183	1957.11.21	Three volumes of *Translated Essays on Dramatic Theory*
184	1957.12.21	Ministry of Foreign Trade should unify translation and publication of manuals for imported equipment
185	1958.01.26	Soviet Union publishes Russian translation of the *Book of Songs*
186	1958.02.04	Chinese translation of *Abstracts of Western Classics* published
187	1958.02.27	Introducing the "Literary Theory Translation Series"
188	1958.03.06	Translation style also needs reform
189	1958.03.26	Institute of Philosophy spurs itself on — revision plans speed up compilation and translation
190	1958.04.14	Talking about translation
191	1958.05.29	A word to translators and critics
192	1958.11.27	Outline of Khrushchev's report on national economic control figures — People's Publishing House to translate and publish soon
193	1958.11.30	Outline of Khrushchev's report translated into Chinese and published
194	1958.12.25	Reflections prompted by the proper translation of "distribution according to work, distribution according to need"
195	1959.01.24	From *Yiwen* to *World Literature*
196	1959.02.01	Chinese translation of Khrushchev's report to the CPSU Congress soon to be distributed nationwide
197	1959.02.18	Classic works should be introduced, translations should be reviewed
198	1959.03.07	Brief review of *Guide to May Fourth Period Journals* — compiled by the Research Office of the Central Compilation and Translation Bureau of the Works of Marx, Engels, Lenin and Stalin, published by People's Publishing

		House
199	1959.03.25	Complete Chinese translation of *Don Quixote* published
200	1959.03.28	Comments on the translation of the term "bourgeois right"
201	1959.04.01	A casual talk on translated terms
202	1959.04.22	Lenin's works translated into eighty-eight languages, over 300 million copies issued — Revolutionary truths spread to hundreds of millions
203	1959.06.03	In 1956 the Soviet Union ranks first in publishing scientific and translated books
204	1959.06.08	Pruning and weeding in the field of translation
205	1959.06.17	Abstracts of foreign academic papers — Issue No. 6 of *Academic Translation Series* out today, with summaries of three translated articles
206	1959.07.11	Britain publishes an English translation of *Chu Ci* (*The Songs of Chu*)
207	1959.07.18	Research Institute of Computing Technology and other units actively study machine translation
208	1959.07.18	Soviet research on machine translation makes great progress
209	1959.09.05	Chinese translation of British physicist Bernal's monumental work *Science in History* soon to be published
210	1960.03.27	Soviet audiences love Chinese films — *Wan Shui Qian Shan* and others dubbed into Russian
211	1960.12.22	Electronic computers translate scientific literature; oxygen drilling speeds mine tunneling; new dry-quenching device greatly raises coke output; instruments help swimmers improve technique
212	1961.01.23	A rendering of Yuan Mei's "On Mr. Huang's Borrowing Books"
213	1961.02.10	Chinese translation of *Minutes of the Paris Commune* to be published soon
214	1961.02.11	A rendering of "Zou Ji Remonstrates with the King of Qi"
215	1961.05.04	Lin Zexu as translator of books
216	1961.05.20	What the trend toward "free translation" reveals — At the emergency Tokyo meeting of the Afro–Asian Writers' Conference
217	1961.05.28	Riddle of the Mayan script initially solved — Soviet scientists use electronic computers to decipher part of the writing
218	1961.07.06	Commercial Press and other publishing houses translate and publish foreign works in philosophy and the social sciences
219	1961.07.18	Yan Fu's notes to his translation of *The Wealth of Nations* and his economic thought
220	1961.10.18	Cambodian edition of *Snowflakes Flutter* published
221	1961.11.10	On translating Lu Xun's poetry
222	1961.11.14	Telegrams should be properly translated before delivery
223	1961.11.16	Central Committee of the Japanese Communist Party decides to publish Japanese translation of *Selected Works of Mao Tse-tung*
224	1961.11.21	Translating and publishing Chinese books abroad
225	1961.11.29	Contract signed in Beijing to translate and print Japanese edition of *Selected*

		Works of Mao Tse-tung
226	1961.11.29	Volume four of *Selected Works of Mao Tse-tung* completed in Japanese — Liao Chengzhi hosts celebration and farewell banquet for Anzai Kuji and others
227	1962.01.06	Fujian Teachers College compiles and translates materials on imperialist aggression in Fujian
228	1962.01.27	Ministry of Culture and Nationalities Commission invite minority-language translators to discuss improving the translation quality of *Selected Works of Mao Tse-tung*
229	1962.02.25	On machine translation
230	1962.05.22	Chinese translation of volume one of Hegel's *Phenomenology of Spirit* published
231	1962.05.27	A landmark translation project — On the Japanese translation of volume four of *Selected Works of Mao Tse-tung*
232	1962.06.14	On the use and translation of the word 'nation'
233	1962.06.14	Discussion on standardizing the translated term of the word 'nation'
234	1962.06.14	Draft Chinese translation of a long Zhuang lyric poem
235	1962.06.24	Edition of ancient Mayan manuscripts with translation and commentary
236	1962.06.28	Overview of the translation and publication of classical bourgeois political economy works
237	1962.08.07	Translation and publication of Rousseau's works in China
238	1962.08.19	Comments on a script — Preface to a Chinese–English translation of a one-act play
239	1962.09.05	NPC Standing Committee holds its 62nd meeting — Zhang Zhixiang reports on the translation, publication and distribution of Mao Zedong's works
240	1962.09.10	Translation cadres among Yunnan's minority nationalities are emerging
241	1962.09.10	Shanghai completes translation and dubbing of two Cuban feature films
242	1962.09.17	Albania translates and publishes *Mao Zedong on Literature and Art*
243	1962.10.19	CPPCC cultural and educational group holds forum for compilers and translators
244	1962.10.30	Starting from translations of atheist works
245	1962.12.02	Mongolian heroic epic *Jangar* translated into Chinese
246	1962.12.18	Two recent works by Comrade Aidit translated and published
247	1963.02.01	Albania translates and publishes *Selected Poems of Du Fu*
248	1963.03.05	"Again on the Differences Between Comrade Togliatti and Us" published as a separate volume — Four articles and editorials from *Red Flag* and *People's Daily* translated into minority languages
249	1963.03.21	Inner Mongolia Medical College completes compilation and translation of *Mongolian Materia Medica*
250	1963.03.29	Works of Julius Fučík translated into many languages and published

251	1963.05.01	Chinese translation of volume six of *Selected Works of Kim Il Sung* published
252	1963.05.05	China attaches great importance to translating and publishing works of Marx and Engels
253	1963.05.30	A batch of story films translated into Mongolian
254	1963.06.22	Yunnan translates Naxi cultural classic *Dongba Scripture*
255	1963.09.09	Chinese translation of volume five of *Selected Works of Kim Il Sung* published
256	1963.10.24	Collation and translation completed for three ancient agricultural treatises
257	1964.01.06	Dubbed version of *Special Mission* done well
258	1964.01.11	Chinese translation of the wartime volume of *Selected Works of Nosaka Sanzo* published
259	1964.01.26	Chinese translation of volume four of *Selected Works of Kim Il Sung* published
260	1964.02.26	The big myth of U.S. "aid" to underdeveloped countries — Condensed translation from the Feb. 13 issue of the U.S. weekly *National Guardian*
261	1964.03.18	Italian publisher translates and issues in Italian Chairman Mao's talk supporting the Panamanian people
262	1964.03.25	Indonesia translates and publishes *Selected Works of Lu Xun*
263	1964.04.08	On one passage in the Chinese translation of *The Communist Manifesto*
264	1964.05.02	Ceylon publishes translation of Chairman Mao's work *On the People's Democratic Dictatorship*
265	1964.05.17	Ceylon translates two works by Chairman Mao — *On the People's Democratic Dictatorship* already published in Sinhala; *On the Chinese Revolution and the Chinese Communist Party* now being translated
266	1964.06.06	'The White-Haired Girl' and 'Daughters of the Party' translated into African; the Ministry of Culture presents a certificate of appreciation to the Malian dubbing artist
267	1964.06.08	*Red Crag* translated into Japanese Braille
268	1964.07.08	Does the proletariat oppose bourgeois relations of production in the spontaneous stage of struggle? — On one passage in the Chinese translation of *The Communist Manifesto*
269	1964.07.09	Chairman Mao's "Analysis of the Classes in Chinese Society" translated into Tamil and published in Ceylon to warm reader response
270	1964.07.20	China translates and publishes the poetry collection *South Vietnam in Fighting*
271	1964.07.27	Volume four of *Selected Works of Mao Tse-tung* published in Braille
272	1964.09.07	New Beijing Foreign Languages Institute opens — Training returned overseas Chinese students as secondary-school language teachers and translators
273	1964.09.18	A successful experiment and crystallization of friendship — On the

		Bambara-language dubbed versions of *The White-Haired Girl* and *Daughter of the Party*
274	1964.09.20	On the translation and publication of Plato's works
275	1964.11.14	Sweden translates and publishes *Selected Works of Lu Xun*
276	1965.02.02	Lawyer Pinto's defense exposes U.S.–Chiang clique's direct role in persecuting our personnel — Their forged letters and distorted translations are despicable; our personnel's actions are legitimate and cannot be blackened by slander
277	1965.05.20	Johnson's days grow difficult — Condensed translation from the May 16 issue of the U.S. weekly *Time*
278	1965.06.03	*The Polemic on the General Line of the International Communist Movement* translated into four minority languages, soon to be published
279	1965.06.05	Hangzhou produces eight-channel transistor simultaneous interpretation equipment
280	1965.08.01	Learn from the shining example of martyr Nguyen Van Troi — *Live Like Him*, a book on his deeds, translated and published by People's Literature Publishing House
281	1965.09.04	New Communist Party organ carries full text of Mao's *Where Do Correct Ideas Come From?* — Two works by Chairman Mao translated into Sinhala and published in Ceylon; Swedish edition of *Mao Zedong's Military Writings* issued in Sweden
282	1965.09.11	Another work by Chairman Mao translated into Sinhala and published in Ceylon
283	1965.11.16	On the translation of the phrase "Antagonism disappears but contradictions remain"
284	1965.12.17	"Danger Signal" — Condensed translation from the Dec. 6 *New York Herald Tribune*
285	1965.12.30	Chinese translation of Hill's *Looking Back and Looking Ahead*, chairman of the Australian Communist Party (Marxist–Leninist), to be published soon
286	1966.02.18	U.S. draft troubles — Condensed translation from the Feb. 4 issue of the U.S. weekly *Time*
287	1966.02.21	Italian quarterly *East Wind* carries works by Chairman Mao and translated articles from Chinese newspapers on politics, economy, culture and science
288	1966.03.19	Mao Zedong's "Imperialism and All Reactionaries Are Paper Tigers" translated into Khmer and published in Phnom Penh
289	1966.03.31	On a passage by Marx concerning two kinds of rights and its translation
290	1966.04.16	Iraqi poet translates Mao's lyric "Qinyuanchun (Changsha)" into Arabic for publication
291	1966.04.17	English and French editions of *Chinese Literature* publish English and French translations of ten poems by Chairman Mao

292	1966.05.06	Japanese people praise and emulate Wang Jie — Tokyo translation of *The Diary of Wang Jie* warmly received; readers vow to learn from his revolutionary spirit in the struggle against U.S. imperialism and revisionism
293	1966.06.21	Eighteen poems by Chairman Mao translated into Arabic and published in Syria
294	1966.07.26	Albanian magazine *November* translates and publishes four poems by Chairman Mao
295	1966.09.04	Chairman Mao's work *Be Concerned with the Well-being of the Masses, Pay Attention to Methods of Work* translated into Tamil and published in Ceylon
296	1967.01.24	Chairman Mao's brilliant "Three Constantly Read Articles" translated into Sinhala and published in Ceylon
297	1967.03.04	French paper *New Humanity* publishes the "Three Constantly Read Articles" — Mao's three brilliant pieces translated into Malayalam and published in India
298	1967.06.01	Persuading Queen Dowager Zhao (translation)
299	1967.10.11	Every word of Chairman Mao's is truth and power — *Quotations from Chairman Mao* published in Cairo and Iceland warmly welcomed by revolutionary people; Mao's poems translated into Bengali and published in Pakistan
300	1968.03.30	Chilean revolutionary organization publishes Mao's "Three Constantly Read Articles" and other works — Applying the "Three Constantly Read Articles" in revolutionary struggle; Mao's writings translated into Sinhala and warmly received by revolutionary people in Ceylon
301	1968.03.31	Mao Zedong Thought is a brilliantly shining sun — New Japanese translation of volume one of *Selected Works of Mao Tse-tung* warmly received in Japan
302	1968.05.22	Tokyo and Yamaguchi revolutionaries hold rallies to celebrate the publication in Japan of the new Japanese translation of volume one of *Selected Works of Mao Tse-tung* — Mao has developed Marxism–Leninism to a new stage; resolve to apply Mao Zedong Thought in Japan's revolutionary practice and win victory
303	1968.06.23	Revolutionary people in Kyoto solemnly rally to celebrate the publication in Japan of the new Japanese translation of *Selected Works of Mao Tse-tung* — Mao Zedong Thought is the sharpest ideological weapon for defeating imperialism, revisionism and reaction; revolutionaries resolve to study and apply it in close connection with struggle
304	1968.07.30	Spanish edition of volume one of *Selected Works of Mao Tse-tung* distributed in Latin America to warm revolutionary response — Chairman Mao's works translated into Bengali and published in East Pakistan
305	1968.10.17	Three important speeches by Comrade Hoxha translated into Chinese to be

		issued nationwide — Important 24 March article from Albanian paper *People's Voice* also published
306	1968.11.02	Two brilliant works by Chairman Mao translated and published in Ceylon
307	1968.11.04	Communiqué of the 12th enlarged plenary session of the Eighth Central Committee of the Communist Party of China translated and published in five minority languages
308	1968.12.01	Chairman Mao's works published in Pakistan — *On the People's Democratic Dictatorship* and *Mao on People's War* translated into Bengali and published in Dacca; Vice Chairman Lin, Chairman Mao's close comrade-in-arms, has his important work *Long Live the Victory of People's War* translated into Bengali and published
309	1969.01.23	Script of *The Red Lantern* translated into Arabic — Film *Piano Accompaniment to "The Red Lantern"* screened in Tirana to warm popular response
310	1969.01.29	Chairman Mao's poetry collection *Poems of Chairman Mao* translated into Mongolian, Tibetan and Korean minority languages and published
311	1969.03.05	*Selected Readings from the Works of Mao Zedong* (A edition) translated into Sinhala and published in Ceylon — Translator praises Chairman Mao as the greatest Marxist–Leninist of our time and calls his works a treasure book for all revolutionaries
312	1969.03.30	Script of the revolutionary model Peking opera *The Red Lantern* translated into Nepali and published in Kathmandu
313	1969.06.09	Spanish edition of *Quotations from Chairman Mao* published in Mexico — Vice Chairman Lin's political report and the CPC Party Constitution translated into local languages and published in Ceylon
314	1969.06.21	*Warm congratulations* — Albanian documentary dubbed by the Shanghai Revolutionary Film Dubbing Studio
315	1969.07.22	Four important works by Chairman Mao published in India — Bengali translations of Vice Chairman Lin's political report and the CPC Party Constitution also issued in India
316	1969.10.01	As revolutionary movements of the world proletariat and peoples surge forward, revolutionaries in many countries actively translate and publish Chairman Mao's works — Mao's writings now translated into seventy languages in more than sixty countries and regions, with over one thousand editions
317	1969.12.25	Latin American revolutionaries actively publish Chairman Mao's works — In recent years they have printed and translated over one hundred editions despite heavy persecution by imperialism, revisionism and reaction; they declare: "To spread Mao Zedong Thought is to push the revolution forward"
318	1970.04.02	Chairman Mao's work *Oppose Book Worship* translated into Nepali and published in Kathmandu to warm popular welcome

319	1970.05.26	Chairman Mao's solemn statement "People of the world, unite and defeat the U.S. aggressors and all their running dogs!" translated into five minority languages including Mongolian and Tibetan and published
320	1970.09.15	"Communiqué of the Second Plenary Session of the Ninth Central Committee of the CPC" translated and published in five minority languages and eleven foreign languages
321	1970.09.20	Chairman Mao's work *On Policy* translated into Sinhala and published in Ceylon
322	1970.10.06	Chairman Mao's works and solemn statement translated and published in Ceylon to warm welcome — Communiqué of our Party's Ninth Central Committee Second Plenary Session also translated and published in Ceylon
323	1971.04.01	*Long Live the Victory of the Dictatorship of the Proletariat* translated and published in Mongolian, Tibetan, Uygur, Kazakh, Korean and other minority languages
324	1971.12.26	Our country translates and publishes *The Communist Manifesto* in five minority languages
325	1972.04.22	Italian translation of Chairman Mao's poems published and distributed in Italy
326	1972.08.11	Our chargé d'affaires ad interim in Zambia Hou Qiwen and interpreter Zhang Xinkui die tragically — Zambian President Kaunda, Vice President Chona and others visit our embassy to offer condolences
327	1972.11.07	To meet the study needs of Party members, cadres, workers, peasants, soldiers and revolutionary intellectuals, large numbers of works by Marx, Engels, Lenin and Stalin have been compiled, translated and published in our country over the past two years
328	1973.04.20	Former Chiang clique army major and China Airlines translator Zhao Mingzhe defects and returns to our side
329	1973.07.26	Guangxi film agencies at all levels actively develop film screenings in minority areas
330	1973.09.09	Chinese translations of President Kim Il Sung's speech "Further Strengthen Our Country's Socialist System" and the *Socialist Constitution of the Democratic People's Republic of Korea* published
331	1973.12.09	Syria translates and publishes works by Lu Xun
332	1974.08.11	Xinjiang People's Publishing House translates and publishes "criticize Lin, criticize Confucius" books in minority languages
333	1974.10.06	Editorial "Advance Along the Socialist Road" translated and published in Mongolian, Tibetan, Uygur, Korean and Kazakh
334	1974.12.03	Central Committee of the Japanese Communist Party (Left) re-translates and publishes *The Communist Manifesto*
335	1975.07.24	Albania and Romania translate and publish our novel *Sparkling Red Star*
336	1975.10.10	Chinese translations of two speeches by Comrade Kim Il Sung published

337	1975.11.09	Chinese translation of Marx's *Mathematical Manuscripts* published
338	1975.11.21	Tibet, Qinghai and three other provinces and regions hold coordination meeting on Tibetan-language book translation and publishing — decision taken to translate and publish more and better Tibetan reading materials
339	1976.04.30	English translation of *Poems of Chairman Mao* published
340	1976.08.12	Mongolian book compilation, translation, and publishing industry is developing rapidly in our country
341	1976.11.06	Pakistan and Bangladesh continue to hold events in memory of Chairman Mao — Mao made outstanding contributions to the oppressed peoples; Amharic translation of Mao's works published in Ethiopia
342	1977.05.09	Chinese translation of *Independent Romania, 1877* published
343	1977.06.23	Chairman Hua and Vice Chairman Ye receive more than eight thousand people involved in the translation and publication of volume five of *Selected Works of Mao Tse-tung*, minority students, PLA students and others
344	1977.07.04	Chairman Hua and Vice Chairman Ye meet and host a banquet for foreign experts who helped translate volume five of *Selected Works of Mao Tse-tung* — Comrades Li Xiannian and Wang Dongxing also attend; meeting and banquet filled with warm friendship
345	1977.08.10	Marx's "Machinery, Natural Forces and the Application of Science" (excerpted manuscript) published in the journal *Debates in Natural Science*
346	1977.09.21	Chairman Hua's speeches and articles translated and published in Norway
347	1977.10.04	Chinese translation of Comrade Pol Pot's speech at the Phnom Penh rally marking the 17th anniversary of the founding of the Communist Party of Kampuchea to be published
348	1977.10.19	Calling for reprints of Lu Xun's translated works and others
349	1977.11.14	Greek weekly *People's Struggle* carries article pointing out that Chairman Mao's three worlds theory is based on Marxism–Leninism — Greek edition of volume five of *Selected Works of Mao Tse-tung* translated and published in Greece
350	1977.11.22	Inner Mongolia compiles, translates and publishes a batch of scientific and technical books in Mongolian and Chinese
351	1977.12.12	"Bourgeois right" should be retranslated as "bourgeois rights"
352	1978.04.09	Translator's postscript to *Virgin Soil*
353	1978.04.16	Chinese translation of Comrade Pol Pot's report at the rally marking the 17th anniversary of the founding of the Communist Party of Kampuchea published
354	1978.05.11	*Dream of the Red Chamber* translated into Uygur and published
355	1978.05.17	Romania translates and publishes works by famous Tang poets of our country
356	1978.07.25	Is translating manuscripts in one's spare time "tending a private plot"?
357	1978.08.09	Chinese translations of Comrade Tito's report and closing speech to the

		Eleventh Congress of the League of Communists of Yugoslavia published
358	1978.08.09	Chinese translation of Comrade Ceaușescu's speech at the plenum of the Romanian Communist Party Central Committee published
359	1978.10.14	Translator of Marxist classics Comrade Cao Baohua passes away
360	1978.10.23	Ministry of Education, Ministry of Finance and State Labour Administration jointly issue notice stipulating that university teachers should be paid for extra teaching and for compiling and translating textbooks
361	1978.12.01	Chinese translation of *The Formation of the Unified Romanian National State* published
362	1979.01.04	Jiangsu establishes technical documentation translation and reproduction company
363	1979.02.14	Hohhot sets up amateur translation network
364	1979.02.21	*Collected Translations in Commemoration of Einstein* published and distributed
365	1979.03.05	English translation *Selected Poems of Zhou Enlai* published in Hong Kong
366	1979.03.31	Vigorously promoting the development of literary translators
367	1979.04.10	Japan translates and publishes poetry collection *Fourteen Poems from Premier Zhou's Youth*
368	1979.04.28	Memorial service for famous translator Comrade Fu Lei held in Shanghai
369	1979.05.08	Complete English translation of *Dream of the Red Chamber* published
370	1979.09.23	Comrade Mao Zedong's *Talks with Music Workers* translated and published in five minority languages
371	1979.10.03	Comrade Ye Jianying's speech "At the Rally Celebrating the 30th Anniversary of the Founding of the People's Republic of China" published and to be translated into five minority languages
372	1979.11.08	China Translation and Publishing Service Corporation established in Beijing
373	1979.11.18	"Statues and puppets" — thoughts after translating Fučík's *Notes from the Gallows*
374	1979.11.24	China Foreign Translation and Publishing Corporation founded
375	1979.12.14	Tenth issue of *World Economy Translation Series* published
376	1979.12.15	New translated and annotated edition of *The Secret History of the Mongols* published
377	1979.12.19	Foreign literature journal *Yi Lin* launches its inaugural issue
378	1979.12.22	New Chinese translation of *Red Star over China* by the late famous American writer Edgar Snow published
379	1980.01.04	Though both legs are disabled his will is stronger — Zhu Yang, who painstakingly taught himself English, formally appointed translation editor at Commercial Press
380	1980.01.28	Anthology of translated works on futurology published

381	1980.02.26	Editorial board of the foreign philosophical classics series discusses translation work
382	1980.03.03	*Translation Newsletter* formally launched
383	1980.03.13	Chinese translation of *History of the Chartist Movement* published
384	1980.03.22	A pocket-sized translation machine
385	1980.04.17	Collation and translation of the *Manwen Laodang*
386	1980.04.24	Chinese translation of the U.S. *Encyclopedia of Science and Technology* to be published soon
387	1980.05.12	The work of a young translator
388	1980.06.10	Sesame-flatbread worker becomes university English translator (photo)
389	1980.06.14	Liaoning sets up scientific and technical translation company to tap translation talent widely — organizing full-time, part-time and freelance translators to serve scientific research, production and foreign trade
390	1980.07.14	Chinese translation of *Economic Planning in East and West* to be published soon
391	1980.07.22	Chinese translation of Aristotle’s *History of Animals* published
392	1980.07.25	Chinese translation of *Two Thousand Years of Japan–China Relations* published
393	1980.07.26	Scientific and technical materials on earthworm cultivation now being compiled and translated
394	1980.07.28	Chinese translation of Hegel’s *Philosophy of Nature* published
395	1980.08.11	English translator Tao Zuji uses his spare time to build bridges and open channels, promoting technical exchanges between China and other countries and expanding foreign trade
396	1980.08.21	Chinese translation of *Economics and Public Purpose* published
397	1980.08.21	Memorial service in Beijing for Chen Changhao, loyal proletarian revolutionary fighter of our Party and former deputy director of the Central Compilation and Translation Bureau
398	1980.08.25	Chinese translation of *Talks on Japan’s Economic Growth* published
399	1980.08.25	Abridged Chinese translation of the *Encyclopaedia Britannica* to be published in stages in our country
400	1980.09.04	Reprint planned of *Annotated Translations of Biographies of Political Figures through the Ages*
401	1980.09.11	Zhu Guangqian calls for new, carefully revised translations of Marxist-Leninist classics
402	1980.09.18	Let “a hundred flowers bloom” in the translation field too
403	1980.10.06	*Selected philosophical writings of Lucian* published in Chinese translation
404	1980.11.06	Commercial Press to reprint eight classic works in Yan’s translation
405	1980.11.11	Ma Jian’s translation of the *Qur’an* to be published soon
406	1980.11.15	Translation of *China Shakes the World* published
407	1980.11.20	Demobilized soldier Guan Hao becomes a translator through self-study

408	1980.11.28	*Pangbo*, a new English translation of Mao Zedong's poems, published in Hong Kong
409	1980.12.18	Attach importance to the translation and dubbing of domestic feature films
410	1981.01.12	Commercial Press launches the series *Chinese Translations of World Academic Classics*
411	1981.01.25	Sales clerk Zong Yongqiang uses his spare time to translate geological monographs; Guangzhou Institute of Geography recruits him on an exceptional basis
412	1981.02.03	Chinese translation of *Historical Introduction to Modern Psychology* published
413	1981.02.14	Ambassador Ostojic hosts reception to celebrate publication of the Chinese translation of *Selected Works of Tito*
414	1981.03.23	*On Germany* translated and published in Chinese
415	1981.03.30	Value research on Mao Dun's achievements — postscript to the translation of *Literature of the Dawn: The Chinese Realist Writer Mao Dun*
416	1981.04.10	Ba Jin's classic novel *Family* translated into German and published
417	1981.04.10	A batch of Chinese books recently translated into foreign languages and published
418	1981.04.20	First volume of *Aesthetics Translations* released
419	1981.05.02	*Science Fiction Translation Series* published
420	1981.05.09	Chinese translation of the *Programme of the Romanian Communist Party* published
421	1981.05.09	Introducing the *Film Art Translation Series*
422	1981.07.11	*Collected Translations of Fu Lei* to be published
423	1981.07.16	The *Huacheng* series of translated works
424	1981.07.17	Three documents including the CPC Central Committee's *Resolution on Certain Questions in the History of Our Party Since the Founding of the People's Republic of China* translated into foreign languages and published
425	1981.08.17	Editorial and translation committee for the Chinese edition of the *Complete Works of Hegel* established in Beijing
426	1981.08.25	Japanese Criminal Code, Code of Criminal Procedure and Lawyers Act translated into Chinese and published
427	1981.09.04	Soviet Union translates and publishes *Selected Works of Lu Xun*
428	1981.09.18	Japanese octogenarian translates classic Chinese medical text *Huangdi Neijing*
429	1981.10.05	Chinese translation of *Aristotle's Syllogism* published
430	1981.10.06	Translations by a lame youth
431	1981.10.09	*Modern World Ethics* compiled, translated and published
432	1981.10.12	Translation of the *Zuo Tradition* (*Zuo Zhuan*) published
433	1981.10.24	Qinghai Film Dubbing Studio actively serves minority nationalities
434	1981.11.01	Chinese ambassador presents Chinese translation of the Maltese president's

		poetry collection to the president
435	1981.11.19	Brief introduction to the *Collected Translations of Fu Lei*
436	1981.11.22	French translation of *Dream of the Red Chamber* published in Paris
437	1981.12.08	New Japanese translation of the *Complete Works of Lu Xun* to be published in Japan — People's Literature Publishing House signs contract in Beijing with Japan's Study Research Society and Shuguang Publishing House
438	1981.12.08	*Chronological catalogue of Lu Xun's writings and translations*
439	1981.12.20	Chinese translation of Comrade Kim Il-sung's report to the Sixth Congress of the Workers' Party of Korea published
440	1982.02.01	Preface to *Selected Early Translations of Plays by Zhang Wentian*
441	1982.02.05	Written on the occasion of the launch of the series *Chinese Translations of World Academic Classics*
442	1982.03.16	Nationalities Publishing House translates and publishes a batch of literary classics
443	1982.03.31	Inner Mongolia gives priority to translation and distribution of films in Mongolian
444	1982.04.05	First volume of the *Taiping Heavenly Kingdom History Translation Series* published
445	1982.06.24	Translators Association of China founded in Beijing; Wang Zhen, Ulanhu and others attend and speak
446	1982.08.11	Two-character mistranslation affects availability of a book
447	1982.11.24	Telegraph translator Ji Kedong serves Tibetan people
448	1983.01.05	Institute of Foreign Languages opens enrollment for UN translator training course
449	1983.02.07	From *New Water Margin* to *Translation Bulletin*
450	1983.02.22	Some brief views on translating foreign literary works
451	1983.03.09	Tenth anniversary of the China Foreign Translation and Publishing Corporation
452	1983.03.15	Chinese PEN Center organizes translation of five Cypriot short stories
453	1983.03.15	Translation and publication in China of Marx and Engels' writings on literature and art
454	1983.04.19	Improving the quality of literary translation and nurturing literary translators — journals *Yi Lin* and *Foreign Languages* launch translation competition
455	1983.05.16	New translation of *Outline of World History* published
456	1983.05.21	Yang Shangkun and Wu Xiuquan stress that translators should make greater contributions to the country
457	1983.05.24	Four-thousand-year-old Harappa "seal script" initially deciphered
458	1983.07.31	China trains batches of high-level translators who meet UN requirements
459	1983.08.02	The *Bible* has been translated into 1,763 languages and dialects
460	1983.08.17	An international conference without interpreters — a report from the 68th

		World Esperanto Congress
461	1983.08.30	Precious ancient Japanese historical document translated
462	1983.10.12	Renowned literary translator and Lu Xun scholar Sun Yong passes away
463	1983.10.17	Preparations under way to compile and translate the series *Classics of Western Philosophical Studies*
464	1983.11.09	Award-winning reportage *Chinese Girl* translated and published in Japan
465	1983.11.16	China Translators Association and Beijing Translators Association hold forum for translation professionals
466	1983.12.13	Colombia's *El Tiempo* publishes President Betancur's translation of a lyric by Chairman Mao
467	1983.12.14	Sichuan translates and dubs Chinese and foreign films into Tibetan and Yi
468	1983.12.15	Sixth UN-sponsored translator training course to begin enrollment early next year
469	1983.12.25	Volume I of *Selected Works of Liu Shaoqi* translated into five foreign languages
470	1983.12.28	Microcomputer-controlled automatic Chinese-character translation and teleprinter developed
471	1983.12.31	Beijing edition of the Japanese translation of *Selected Works of Deng Xiaoping* goes on sale
472	1983.12.31	Transliteration of foreign place names should be unified
473	1984.01.06	North Korea translates and publishes Chinese novel *Red Crag*
474	1984.01.16	A sower of friendship — profile of Yu Xinping, translator-guide at Xi'an branch of China International Travel Service
475	1984.01.17	Reminiscences of the original translator
476	1984.01.19	National conference on translator-guides concludes; Wan Li, Chen Muhua and Gu Mu cordially meet all delegates
477	1984.01.23	Pocket electronic translator
478	1984.01.27	Chinese translation of the epic *Jangar*
479	1984.02.17	Preface to the Chinese translation of *My Roots Are in China*
480	1984.02.23	Talking about my translation work
481	1984.03.14	Celebrating Zhang Zhongshi's fifty years of translating and researching Marxist-Leninist works
482	1984.03.26	National Tourism Administration posthumously names Yu Xinping a "National Model Translator-Guide" and launches a campaign in the tourism sector to learn from his example
483	1984.04.06	New 60-volume Chinese edition of the *Complete Works of Lenin* to start publication this year and be completed by 1990
484	1985.04.10	Correspondence program in scientific and technical translation at Beijing Xuanwu Medical and Health Technical Service Center opens enrollment
485	1984.04.13	Middle-aged engineer Wang Tongyi compiles and translates twelve scientific and technical dictionaries in thirteen foreign languages

486	1984.05.07	Chinese translation of *Megatrends* published
487	1984.05.18	Introducing the Chinese translation of *The Biography of Wen Youzhang*
488	1984.05.25	Brief introduction to the *Taiping Heavenly Kingdom History Translation Series*
489	1984.06.15	China has already trained one hundred senior translators for the United Nations
490	1984.06.18	*New Translations of World Literary Classics* series
491	1984.07.04	New automatic translation machine developed in the United States and Japan and put on the market
492	1984.07.10	Computerized Tibetan-script processing system successfully developed; Tibet translates and dubs seven films into Tibetan
493	1984.07.31	Prospects for machine translation look bright
494	1984.08.19	Visiting the European "House of Translators"
495	1984.08.27	Lu Xun and the translation of *Three Men*
496	1984.09.17	Chinese People's Association for Friendship with Foreign Countries and others celebrate publication of the complete Chinese translation of the *Ramayana*
497	1984.09.23	English translation of *Selected Works of Deng Xiaoping* published
498	1984.09.24	Volumes 1–4 of China's first domestically compiled edition of the *Complete Works of Lenin* published
499	1984.10.03	Japan develops automatic translation system
500	1984.11.24	National commendation conference on films translated into minority languages held in Beijing; Ulanhu and others attend to offer congratulations
501	1984.11.30	Seventh UN translation training course to open for enrollment
502	1984.12.05	Translator's eye, publisher's boldness and other matters
503	1984.12.12	Translation and annotation of *Records of the Grand Historian* speeding ahead
504	1984.12.17	Li Jianwu's translation of *Molière's Comedies*
505	1984.12.20	A devoted promoter of ethnic cultural exchange — Interview with Yasen Awazi, translator of the Uygur edition of *One Hundred Tang Poems*
506	1984.12.21	Sections on Xinjiang in the *Twenty-Four Histories* now being translated into Uygur
507	1985.03.09	She left her heart in the motherland — Profile of Lin Fang (Yu Fuying), an overseas Chinese translator in Japan
508	1985.03.29	Chairman of the League of Communists of Yugoslavia receives Chinese translators of *Tito's Memoirs*
509	1985.03.30	Fifth Chinese translation of *Dialectics of Nature* published
510	1985.04.10	Beijing Xuanwu Medical and Health Technical Service Center opens correspondence program in scientific and technical translation
511	1985.04.16	Thoughts prompted by the Japanese translation of *The Complete Works of Lu Xun*

512	1985.04.23	On the publication of the Japanese translation of *The Complete Works of Lu Xun*
513	1985.05.07	Preface to *Modern Translations of Du Fu's Poems*
514	1985.05.11	Preface to *Annotated Translation of Records of the Grand Historian*
515	1985.06.05	Fu Lei's family present *Collected Translations of Fu Lei* to three French libraries
516	1985.07.02	Ancient Chinese mathematical classic *The Nine Chapters on the Mathematical Art* translated into French
517	1985.07.11	Seems easy yet so arduous — Profile of Yang Benwen, compiler–translator at the Ministry of Health Academy of Traditional Chinese Medicine
518	1985.07.16	Japan develops computer system for translating Japanese into English
519	1985.07.29	Middle-aged and young people become main force in literary translation — China's literary translation sector enjoys unprecedented prosperity
520	1985.08.14	National academic symposium on minority-language translation concludes
521	1985.08.30	Memorial service in Beijing for Ke Bonian, outstanding Party member and veteran translator of Marxist-Leninist works
522	1985.09.16	A soldier "interpreter-officer"
523	1985.10.25	Correcting a translated passage of Lenin on "patriotism"
524	1985.11.17	Three classic works of traditional Chinese medicine translated into English for the first time
525	1985.12.07	Three ancient Chinese philosophical works translated into Persian
526	1985.12.18	Compiler–translator Zheng Yili devises new computer coding method for Chinese characters
527	1985.12.25	I love Qu's translation of *The Stormy Petrel*
528	1986.01.07	Eighth intake of the UN interpreter training department at Beijing Foreign Studies University to enroll students
529	1986.01.20	Hunan edition of *Prose Translation Series* to appear successively
530	1986.02.24	Introducing the *Aesthetics Translation Series*
531	1986.03.10	Monument to a distinguished translator — *Collected Translations of Fu Lei* published
532	1986.03.19	Translation department of Beijing Industrial Talent Development Center provides services for technology import
533	1986.04.22	Automatic translation telephone
534	1986.04.27	Reform and opening bring springtime to translation work — First national congress of the Translators Association of China closes in Beijing
535	1986.05.05	Thirty years of effort in translation and publishing — 50-volume Chinese edition of the *Collected Works of Marx and Engels* completed, including over 2,000 works, more than 4,000 letters and over 400 documents
536	1986.05.14	Memorial service in Beijing for Professor Wang Li, distinguished linguist, educator, poet and translator
537	1986.05.18	Development of translation work and the new issues it faces

538	1986.06.08	A vast translation project and a rich theoretical treasure-house
539	1986.06.24	“Poetry Garden” — A selection of translated poems
540	1986.07.13	Author’s preface to *Selected Works and Translations of Bing Xin*
541	1986.07.20	Chinese translation of the *Soviet Military Encyclopedia* published
542	1986.07.21	Confession of a great democrat — Selected translations of Edgar Snow’s letters home
543	1986.07.22	Confession of a great democrat — Selected translations of Edgar Snow’s letters home
544	1986.07.23	Confession of a great democrat — Selected translations of Edgar Snow’s letters home
545	1986.08.02	Chinese translation of Snow’s *Travel Notes on Wartime Soviet Union* discovered in Beijing
546	1986.08.07	*Translations of Famous Foreign Thinkers* series begins publication
547	1986.09.01	European Community to develop computerized rapid translation system
548	1986.09.02	Mayan script deciphered
549	1986.09.06	Chinese edition of the *Concise Encyclopaedia Britannica*, a large reference work jointly compiled by China and the U.S., now completed
550	1986.09.11	An amateur translator of *Faust*
551	1986.09.15	Romania publishes selected translation of the *Book of Songs*
552	1986.09.26	Belgium translates and publishes an issue of the *People’s Daily* to promote Sino–Belgian cultural exchange
553	1986.10.07	King of Spain confers decorations on translator Yang Jiang and teacher Lesea
554	1986.10.10	Chinese translation of *A Brief History of the Workers’ Party of Korea* published
555	1986.10.13	Classic novel *Yama Street* translated and published
556	1986.11.22	One issue of the *People’s Daily* translated into Dutch and published
557	1986.11.24	Mistranslation and misunderstanding of “bourgeois rights”
558	1986.11.28	Fei Xiaotong’s *Jiangcun Economy (Peasant Life in China)* translated into Chinese and published
559	1986.11.28	Over 300 compilers and translators to bring out more than 20 titles next spring — *Twentieth-Century Library* series to introduce contemporary social science classics
560	1986.12.06	UN-commissioned interpreter training department in China to enroll a new class
561	1986.12.20	Elorduy’s translation of the *Book of Songs* wins Spain’s national translation prize
562	1986.12.26	Chinese translation of the *Soviet Encyclopedic Dictionary* to be published soon
563	1987.01.07	Translation of the new *Cambridge World Modern History* completed — Volumes to be issued in the first half of this year

564	1987.01.16	Professor Feng Zhi, long devoted to translating Goethe and Heine, wins award in West Germany
565	1987.01.18	Sun Yat-sen, pioneer of the Chinese term for "economics"
566	1987.01.25	Rongshui Miao Autonomous County film company, rooted in Miao areas, runs a five-person Miao-language film dubbing "studio"
567	1987.01.27	Wang Xiancai single-handedly translates monumental *Harrison's Principles of Internal Medicine* — Ministry of Health gives him a special award for medical translation
568	1987.02.09	Czechoslovakia translates and publishes *Dream of the Red Chamber*
569	1987.02.15	The originator of the Chinese term for "economics" was not Sun Yat-sen
570	1987.02.26	New edition of the *Soviet Encyclopedic Dictionary* translated and published
571	1987.02.28	Reflections on establishing translation awards
572	1987.02.28	Some of Qian Zhongshu's works translated into French
573	1987.03.06	A new book that fills a gap — Delighted to read *Modern Translation of the Great Tang Records on the Western Regions*
574	1987.03.18	On poetry criticism and translated poetry — A conversation with Professor Wang Zuoliang
575	1987.03.24	Swedish translation and distribution of the *People's Daily*
576	1987.03.26	Yao epic *Miluotuo* translated into Chinese
577	1987.03.29	Qinghai dubs many films into Tibetan
578	1987.04.02	China's first machine-translation system for English imitates human thinking
579	1987.04.02	Batch of Marxist-Leninist works and writings by Mao Zedong translated and published in five minority languages
580	1987.04.03	Shanghai Film Dubbing Studio has dubbed nearly one thousand films in thirty years
581	1987.04.21	Chinese translation of the autobiography of "Honorary Citizen of Gansu" Rewi Alley published
582	1987.04.24	Casual reflections on reform in literary translation
583	1987.05.05	Xinjiang dubs two minority-language films each week — New Uygur and Kazakh films can be released simultaneously with Chinese versions
584	1987.05.07	Translating Soviet revolutionary literature inspired generations of aspiring youth — Academic symposium in the capital honors veteran writer and translator Cao Jinghua
585	1987.06.04	Chinese translation of *Art and Illusion* to be published
586	1987.06.10	Echoes still linger in my heart — Notes on the Chinese translation of *Voices After the Catastrophe*
587	1987.06.13	Two books including *Building Socialism with Chinese Characteristics* translated and published in five minority scripts
588	1987.06.19	China has trained more than 160 high-level translators for the United Nations

589	1987.06.28	American friend Mrs. Song Deshi translates *A Tour of Chinese History* and donates 5,000 copies to our History Museum
590	1987.06.28	Review of *Annotated Translation of the Seven Military Classics*
591	1987.07.11	The *Daily Translation Bulletin* during the War of Resistance Against Japan
592	1987.07.28	Posthumous work *Challenge* by woman journalist Yang Gang published — English autobiographical novel kept in the U.S. for many years now translated into Chinese
593	1987.08.02	On horseback he met the enemy's fire, off horseback he studied military texts — Interview with General Guo Huaruo, translator and annotator of *Sunzi* (*The Art of War*)
594	1987.08.06	Renowned Chinese translator Cao Jinghua awarded the Soviet Order of Friendship of Peoples
595	1987.08.12	China has dubbed more than 250 films into Tibetan in recent years
596	1987.08.14	British Telecom develops successful "translation telephone"
597	1987.08.15	English edition of the *Compilation of the Laws of the People's Republic of China* published under the auspices of the NPC Standing Committee's Legislative Affairs Commission
598	1987.08.21	Minority-language translators gather in Zhelimu for academic exchanges
599	1987.08.23	Lu Xun and Gu Hong'er — On a newly discovered page of Lu Xun's translation manuscript
600	1987.09.08	Renowned translator and writer Cao Jinghua passes away
601	1987.09.26	A century-old *Leaves of Grass* still fresh and green — On reading the complete Chinese translation of *Leaves of Grass*
602	1987.10.18	Giving translated literature its proper place
603	1987.10.19	Chinese translation of Snow's *Travel Notes on Wartime Soviet Union*, translated by Sun Chengpei over forty years ago, reissued
604	1987.10.22	*Shanghai Translation News* to switch to a four-fold, eight-page format next year
605	1987.10.25	Shanghai should have an efficient editing and translation agency
606	1987.10.28	Chinese comic book *The True and False Monkey King* translated and published in Poland
607	1987.11.06	Translators Association of China formally joins the International Federation of Translators
608	1987.11.10	Reading Ge Baoquan's new translation *Collected Poems of Pushkin*
609	1987.11.10	Symposium on Soviet poetry translation held in Beijing
610	1987.12.02	Series of translations of major works by Nobel economics laureates to be published soon
611	1987.12.07	Translating the book title *Oh, Man, Man!*
612	1987.12.11	China and Japan jointly develop multilingual automatic translation system
613	1987.12.13	Who translated "philosophy" as "zhexue"?
614	1987.12.24	Series *Classic Works in Cultural Anthropology in Translation* to be

		published
615	1988.01.07	Comrade Jiang Chunfang, noted translator and editor–publisher, passes away
616	1988.01.13	Renowned translator Ge Baoquan wins the Pushkin Literature Prize
617	1988.01.21	Interpreter's mistranslation provokes laughter (photo)
618	1988.01.28	Four generations, five people, seven million characters translated — A Yi peasant family is a true "translator clan"
619	1988.02.13	Seven Chinese editions of Bukharin's works to be published
620	1988.02.16	English translation of *The Tianjing Incident* published in the United States
621	1988.02.22	First German edition of volume one of *Capital*, in its original form as written by Marx, translated and published in China for the first time
622	1988.02.22	Love for the green hills — Reading the Chinese translation of *Green Hills Green*
623	1988.03.16	China translates and publishes Gorbachev's *Perestroika: New Thinking for Our Country and the World*
624	1988.03.31	"Senior translator"
625	1988.04.08	Looking for an "interpreter" for "Boss Rong"
626	1988.04.23	There is a Mr. Dong in China — Profile of Dong Yinglie, interpreter at China International Travel Service
627	1988.05.08	Britain exhibits automatic telephone translation system
628	1988.05.09	Chinese translation of an Indian classic and another book published; Indian ambassador in China hosts reception in Beijing
629	1988.05.13	Chinese translation of *An American Looks at Old China* published
630	1988.06.02	China publishes 111 law textbooks in eight years; *International Law* now being translated and published abroad
631	1988.06.09	Major results in folk art and folklore research — Jin Zhilin deciphers a group of primitive cultural symbols
632	1988.06.20	Primitive pictographic script at Shipengshan deciphered; new evidence that the name "Yanshan" dates back to the Yin–Shang period
633	1988.06.29	Machine translation technology revives in France — Software for translation among six Western languages enters computers
634	1988.07.01	Chinese translation and adaptation of the film *The Last Emperor* completed
635	1988.07.30	Paralyzed for over thirty years, Chen Gongyi has translated more than one million characters and receives "Self-taught Success for the Disabled" honor
636	1988.07.31	Breakthrough in deciphering Dunhuang dance notation unread for eighty-eight years
637	1988.08.11	*Great Dictionary for the Appreciation of World Classics* fully translated into Chinese
638	1988.08.13	West German researcher claims he has deciphered the Mayan script
639	1988.09.08	Looking for English–Chinese translation? "TransStar" intelligent machine

		translation system now widely used
640	1988.09.13	More than four hundred works translated and published in minority languages
641	1988.10.23	Translation in ancient China
642	1988.10.23	The translated titles of *Water Margin*
643	1988.10.25	Exchange meeting for middle-aged and young literary translators
644	1988.10.26	Joy and concern in literary translation — Experts call for improving the quality of translated works
645	1988.11.05	Machine translation technology develops rapidly in France — Gachot company launches software for translation among six Western languages
646	1988.11.17	Chinese translation of the *Oxford Dictionary of Law* published
647	1988.11.30	English translation of *Zhouyi Cantongqi* published
648	1989.01.09	Series *Chinese Translations of Classics in International Politics* to be published
649	1989.01.25	The *Oxford Dictionary of Law* and its translator
650	1989.01.31	Chinese Academy of Arts selects research achievements for the first time — Outstanding monographs, papers, reviews, materials and translations win awards
651	1989.02.28	Decoding the communication code of bees — Artificial "bees" attract real bees
652	1989.03.10	Japan trial-produces multi-language simultaneous translation system
653	1989.04.05	In one translation of a world classic lie bitterness, bleakness and hardship — Recollections of editing and translating *And Quiet Flows the Don*
654	1989.04.14	Import–export imbalance in literary works — Foreign promotion and translation urgently need strengthening
655	1989.05.03	Chinese translation of Pearl Buck's *The Good Earth* trilogy published
656	1989.05.14	Translator Zhang Xichou passes away
657	1989.05.24	Western Xia feudal regime ruled the country by law — Translation of the *Western Xia Code* published
658	1989.07.19	Deng Xiaoping's writings and the Fourth Plenum communiqué translated into minority languages and published
659	1989.08.06	Shanghai Translation Publishing House puts social benefit first and wins readers through book quality
660	1989.08.08	Luo selects and translates ancient Chinese novels for publication
661	1989.09.09	China's first translated work introducing Marx's thought — *Essays on Western Civil Law*
662	1989.10.03	Training course on translating minority literary works into Chinese opens
663	1989.10.20	*Twentieth-Century Foreign Educational Classics* translation series
664	1989.10.24	Annotated and translated edition of *Records of the Grand Historian* published in Xi'an
665	1989.10.31	"Yi oracle bone script" in Guizhou deciphered

666	1989.11.24	Qian Yuzhi deciphers the “Ba–Shu symbols,” opening a new path for exploring ancient Shu civilization
667	1989.12.06	The rise of translation companies
668	1989.12.10	Scholars say Chinese translations of world academic classics, produced under Marxist guidance, have fundamental significance for China’s academic culture
669	1989.12.14	Burmese writer’s translation of *Dream of the Red Chamber* wins Myanmar National Literature Prize
670	1990.01.20	A special history of a century of translation — Reading *A Draft History of Chinese Translation Literature*
671	1990.01.21	Saving energy through real skills and hard work — Jiang Zemin’s translated work *Rational Use of Electric Power in Machine-Manufacturing Plants* published
672	1990.02.05	Poetry translator Feibai compiles *Sea of Poems*
673	1990.02.18	AT&T says it can provide telephone simultaneous interpretation services worldwide
674	1990.02.20	Selected readings of Marxist-Leninist works translated and published in seven minority languages
675	1990.03.02	Important works by Comrade Deng Xiaoping translated into multiple minority languages and published
676	1990.03.25	Outstanding interpreters are hard to find
677	1990.03.25	Mother and daughter hone their skills in simultaneous interpreting
678	1990.04.20	A translation rich in academic value — On Tian Dewang’s translation of *The Divine Comedy: Inferno*
679	1990.04.25	Chinese translation of the novel *Confucius* to be published soon
680	1990.05.29	A distinctive scene — On Jin Jianguo and the Jins’ translation of Russell’s collected works
681	1990.07.28	Scriptures brought back from the Western Regions, painstakingly translated over the years — Rare complete edition of Xuanzang’s translations published
682	1990.08.08	Seven-hundred-year-old Mongolian lyric poems — *Birch-bark Book of the Golden Horde* translated into Chinese
683	1990.08.22	Largest modern-language series of ancient classics since the May Fourth movement published — *Selected Translations of Ancient Classics in Literature and History* launches
684	1990.08.23	Twelfth World Congress of the International Federation of Translators opens; Chinese representative elected to the council
685	1990.09.09	Chinese translation of Joseph Needham’s monumental *Science and Civilisation in China* issued
686	1990.09.21	Giving Chinese songs wings — Profile of Zhang Qingnian, English translator of Asian Games songs

687	1990.10.04	Reporter doubles as interpreter
688	1990.11.13	An original "decoding" of Li He's poetry
689	1990.12.05	Scholars call for improving the quality of English translations in external publicity
690	1990.12.05	*Fortress Besieged*: The eighth "translation" is television
691	1990.12.08	Mainland Chinese translation of *Capital* published in Taiwan
692	1991.01.02	Computerization of conversion between Chinese characters and Braille — Blind readers expected to be able to read the same day's newspaper
693	1991.01.10	Chinese translation of the *Great Dictionary for the Appreciation of World Classics* published
694	1991.01.11	Chinese translation of *Hippocratic Writings* published
695	1991.03.27	Simultaneous interpreting
696	1991.03.31	Fan Weixin wins Brazil's "Best Foreign Translator" award
697	1991.04.07	Kyrgyz text and Chinese translation of the epic *Manas* published
698	1991.04.20	Tibet's film translation and dubbing industry
699	1991.04.26	Forum in the capital celebrates publication of the new edition of the *Complete Works of Lenin* — Li Ruihuan calls it a major achievement in compiling and translating Marxist-Leninist classics
700	1991.05.02	Passing on the flame of truth — Interview notes from the CPC Central Compilation and Translation Bureau of the Works of Marx, Engels, Lenin and Stalin
701	1991.09.11	Telephone translation device
702	1991.10.25	*Complete Translations of Famous Chinese Classics Through the Ages* published — Fifty representative classics in the classics, history, philosophy and belles lettres from pre-Qin to Qing selected; full series to be completed in 1995
703	1991.11.11	This too is a form of translation art
704	1991.12.04	Marx's *Historical Notebooks* translated into Chinese — This important work leaves rich material and key evidence for later generations to understand Marx's view of history and his historical method
705	1991.12.04	Thoughts prompted by *The Comprehensive Vernacular Translation of the Comprehensive Mirror for Aid in Government*
706	1991.12.07	Selected translations of deciphered Tang and Song songs to be published
707	1991.12.11	Facing the wall for over ten years to crack an ancient riddle — Yin Boling renders the Stone Drum inscriptions as a vernacular epic
708	1991.12.16	Hut of thatched roof and lamplight — To Mr. Chen Wangdao, the first translator of *The Communist Manifesto* into Chinese
709	1992.01.04	Sixty-year-old farmer Li Quanzhen adapts and sings the Peking opera *Ganlusi (甘露寺)*
710	1992.01.05	Records of "firsts" in translating and publishing the works of Marx and Engels

711	1992.01.14	Li Peng tells delegates to the National Conference of CITS tour guides and interpreters: China's tourism industry has great potential for development
712	1992.01.21	Publicity Department of the CPC Central Committee commends the compilers and translators of the second Chinese edition of the *Complete Works of Lenin*
713	1992.02.04	*Essence of the Twenty-Six Histories in Modern Translation* well received
714	1992.02.23	The first person to translate Mao Zedong's works into English
715	1992.02.24	A precious document unfolding a historical panorama — Written for the publication of the Chinese translation of Marx's *Historical Notebooks (early 1880s)*
716	1992.03.11	The translator's obsession must savour its flavour — Interview with Luo Xiwen, translator of traditional Chinese medical classics
717	1992.04.04	Recreating the life of a great translator
718	1992.04.08	First Japanese–Chinese Translation Award announced in Beijing
719	1992.04.11	Chasing a translation dream at Yilin — A profile of Yilin Publishing House
720	1992.05.06	Selecting the essence to show the grand panorama, rich in text and images — Reflections on compiling and translating the illustrated *History of World Literature*
721	1992.05.09	Complete Chinese translation of the works of Gibran
722	1992.05.27	*Oil, Money and Power* translated into Chinese
723	1992.06.12	Translation service company established in Jiamusi
724	1992.06.27	Gaoli Company launches high-tech English–Chinese and Japanese–Chinese computer translation systems
725	1992.07.19	Intelligent machine translation system passes appraisal
726	1992.08.31	*Selected Translations of Japanese Scholars' Studies on Chinese History* published
727	1992.10.12	The "heavenly script" of Dunhuang musical notation deciphered
728	1992.10.15	Good translations of Tang poetry
729	1992.11.07	A telephone with translation function
730	1992.11.08	Italy translates and publishes a collection of Guo Moruo's poems
731	1992.12.06	Before and after the translation and publication of the Chinese edition of Marx's *Historical Notebooks* — In memory of Comrade Zhang Youyu
732	1993.01.15	Automatic telephone translation system successfully tested
733	1993.01.28	New photocopier with translation function
734	1993.01.30	"Ni hao" becomes "hello" — A telephone that can translate by itself
735	1993.02.21	Who can crack eight mysterious scripts?
736	1993.02.22	The whole story of translating and publishing the Chinese edition of Marx's *Historical Notebooks* — In memory of Comrade Zhang Youyu
737	1993.03.02	Work on the second Chinese edition of the *Complete Works of Marx and Engels* launched — Jiang Zemin, Yang Shangkun and Li Peng inscribe congratulations for the 40th anniversary of the Central Compilation and

		Translation Bureau
738	1993.03.18	China Foreign Translation and Publishing Corporation celebrates its 20th anniversary
739	1993.03.20	Female interpreter and waitress (photo)
740	1993.04.10	Chen Zhaoxiong makes major breakthroughs in machine translation — Intelligent MT theory and technology set five world firsts
741	1993.05.02	Helping more Chinese people understand Samaranch — Written on the eve of the Chinese publication of *Samaranch and the Olympic Movement*
742	1993.05.14	China National Radio adopts a new English name
743	1993.05.29	Chongqing University builds an archive database of contemporary Chinese translators
744	1993.06.12	Complete Chinese translation of the Mongolian epic *Jangar* published
745	1993.06.15	Exhibition of Guo Moruo's writings and translations held
746	1993.06.26	*Comprehensive Vernacular Translation of the Essential Twenty-Five Histories* published
747	1993.07.28	"Bi's Translations of Zola's Classics" series published
748	1993.08.02	*Modern Translations of Ancient Chinese Classics* series begins to appear
749	1993.08.09	Germany develops a spoken-language translation device
750	1993.08.12	A sincere friend — Preface to the Chinese translation of *Eighteen Years of Japan–China Relations*
751	1993.09.18	ChinaSoft Corporation releases Chinese–English and Chinese–Japanese machine translation systems
752	1993.10.22	The "Heavenly Scripture" is rendered in Kazakh, a blessing for Muslims — The *Qur'an* now has a Kazakh-language edition
753	1993.10.22	The birth of a translated work
754	1993.10.22	A charming new translated edition to appreciate
755	1993.10.25	*Overseas Studies on Deng Xiaoping* translation series published
756	1993.10.28	Adding a touch of warmth to the human world — On Zhu Guangqian's translation of Vico's *New Science*
757	1993.12.03	"Translator," a bridge of friendship and cooperation
758	1993.12.11	*The Socialist Road to Power* published in Chinese translation
759	1993.12.17	Interpreters take part in cross-provincial exchange activities
760	1993.12.27	Long-term work in language and translation
761	1993.12.27	"English — a bridge to the world"
762	1994.01.05	Translator and interpreter competitions held nationwide
763	1994.01.25	Pan Hannian: one of the earliest translators of *Capital* and *The Communist Manifesto*
764	1994.03.07	European Community develops multilingual translation system
765	1994.03.22	The Bible translations of Schereschewsky and others
766	1994.04.19	The man who translated *Laozi* and the *Book of Songs* into English —

		Professor Wang Rongpei of Dalian University of Foreign Languages
767	1994.04.22	Commercial Press devoted to introducing the intellectual and cultural essences of all nations — "Chinese Translations of World Academic Classics" series reaches 300 titles
768	1994.04.29	Mathematical master deciphers famous code
769	1994.05.26	"Kuaiyitong Cup" tennis tournament held
770	1994.06.11	Chinese translation of *History of Japan's Ministry of International Trade and Industry Policy* published
771	1994.06.14	Chinese and U.S. publishers sign copyright agreement in Beijing — Chinese edition of *World Encyclopaedia* to begin compilation and translation
772	1994.07.13	Chinese translation of *The Memoirs of Paik Beom (Baekbeom Ilji)* published
773	1994.07.15	First Chinese translation of *History of Political Philosophy* published
774	1994.07.22	Multifunctional flight-data recorder decoding equipment unveiled
775	1994.07.29	Complete Chinese translation of *Ulysses*
776	1994.08.02	Belated brilliance — Brief note on *The "Bi's Zola Translations" Selected Library of Famous Translations of Famous Works*
777	1994.08.02	Telegraph operator's carelessness: a happy piece of news becomes bad news
778	1994.09.20	Princess Sirindhorn translates Chinese novel *Butterfly*; Thai edition released
779	1994.10.08	After the softball match, reporters battle interpreters; young table-tennis player plays star
780	1994.10.08	New version of the "Translation Star" machine released
781	1994.11.02	Modern translations of ancient texts must not be done perfunctorily
782	1994.11.19	Nearly a thousand volumes of Dongba pictographic classics completely translated
783	1994.12.10	English translation of *Romance of the Three Kingdoms* published — English versions of the four great classical novels of Chinese literature now complete
784	1995.01.16	Many languages in the European Union keep "translation bridges" busy
785	1995.01.24	On the issue of modern translations of ancient texts
786	1995.02.01	How should an open China treat foreign cultures? — Reflections prompted by the publication of *Chinese Translations of World Academic Classics*
787	1995.02.04	Hangzhou enthusiastically supports the dubbed-film industry
788	1995.02.09	One thousand volumes of Dongba classics translated
789	1995.03.01	Is the translation of "zhishi chanquan" as "intellectual property rights" correct?
790	1995.04.03	Importing a foreign edition costs far less than sending delegations abroad — Slump in publication of translated science and technology books cause

		for concern
791	1995.04.20	Chinese and foreign scholars discuss *Ulysses* — Xiao Qian tells in detail how he translated the book
792	1995.05.13	On the retranslation of famous works
793	1995.05.29	Selecting one hundred of the most representative books in the history of Chinese civilisation, translating them into foreign languages and sending them abroad to give the world a pleasant surprise — Notes from the *Great Chinese Library* symposium
794	1995.06.01	Farewell ceremony for Denise's remains held in Beijing — Li Fengbai–Denise Outstanding Foreign Book Translation Fund to be established
795	1995.06.25	Sixteen years of painstaking work — Interview with Sandra Lavagnino, translator of *The Literary Mind and the Carving of Dragons*
796	1995.06.25	Use of transliterated loanwords needs to be standardised
797	1995.07.11	A major undertaking for the translation and publishing sector
798	1995.08.01	Asian Translators Forum convenes in Beijing
799	1995.08.29	What will happen to translated books after China joins the Berne Copyright Convention? — Electronic publishers: great potential in co-publishing with foreign partners
800	1995.09.02	African woman holding a translation device
801	1995.10.16	Chinese and foreign gems weave a "world spiritual park" — Three hundred titles now published in the "Chinese Translations of World Academic Classics" series
802	1995.10.17	Devoting a lifelong ambition to translation — *The Iliad* and its translator Luo Niansheng
803	1995.10.24	Major breakthroughs in our machine translation research — Full-text English–Chinese / Chinese–English computer translation system developed and put into practical use
804	1995.10.27	An important project in the Party's ideological and theoretical work — Report on compiling and translating the new editions of Marxist–Leninist works
805	1995.10.27	Latest achievements in compiling and researching Marxist classics — New editions of Marxist–Leninist works published
806	1995.10.28	Rich fruits of the career of compiling Marxist–Leninist works — Congratulations on the publication and distribution of the new editions
807	1995.10.29	New translation of the complete Andersen fairy tales launched
808	1995.11.24	Launch ceremony and seminar for the new translation of *Don Quixote* held in Beijing
809	1995.12.04	Writers, translators and publishing workers call for respect for authors' and translators' rights
810	1995.12.13	Translation and publication project for the *Complete Works of Einstein* launched

811	1995.12.21	New achievements of the career of compiling Marxist–Leninist works (I) — Introducing the second Chinese edition of *Selected Works of Marx and Engels*
812	1995.12.22	New achievements of the career of compiling Marxist–Leninist works (II) — Introducing the third Chinese edition of *Selected Works of Lenin*
813	1995.12.26	New achievements of the career of compiling Marxist–Leninist works (III) — Introducing the second Chinese edition of the *Complete Works of Marx and Engels*
814	1996.01.30	Bringing in "stones from other hills" — "Chinese Translations of World Academic Classics" series reaches 260 titles
815	1996.03.07	Celebrating the publication of the complete translation of *The Human Comedy*
816	1996.04.02	Xi'an Translation Training College has trained more than ten thousand students
817	1996.04.10	Have you ever thought of me? — The Tongyi translation software speaks
818	1996.04.28	Translating Chinese literature — Interview with Ukrainian translator Chirko
819	1996.06.10	"Huanqiutong" Chinese–English translation software launched
820	1996.06.29	Second national conference on minority-literature translation held in Chifeng, Inner Mongolia
821	1996.08.06	Translators and experts hold forum on translation
822	1996.08.09	A brief discussion of "not worthy of English translation"
823	1996.08.22	Shenhua shine in Asian Club Cup, crush Kuaiyitong 7–1
824	1996.09.05	In Asian Club Cup, Shanghai Shenhua again defeat Kuaiyitong
825	1996.09.11	Chinese translation of the epic *Manas* basically completed — Experts say the northern Tianshan region is the homeland of *Manas*
826	1996.11.01	Wang Rongpei completes English translations of four ancient Chinese classics
827	1996.11.07	Tongyi software offers fast two-way translation
828	1996.11.11	Complete Korean translation of *Records of the Grand Historian* published in Seoul
829	1996.11.18	Sichuan launches English–Chinese translation device
830	1996.11.29	*The Rise of China's First Dynasty — Decoding the Mystery of the Origins of Chinese Civilization and the State*
831	1996.12.08	On the translation of the term "karaoke"
832	1997.01.22	Erudite yet accessible, pleasing both refined and popular taste — Chinese translations in the "Masterpieces of Great Scientists" series released
833	1997.01.27	Should the post and telecommunications bureau bear liability for mistranslating a telegram?
834	1997.02.05	Rethinking science and the fate of humanity — The exploration of the "Science and Humanity Translation Series"

835	1997.02.12	*Essentials of Business Administration* translation series published
836	1997.02.15	Fourteenth edition of *Economics* translated and published
837	1997.05.04	Let history bear witness — Jiangsu People's Publishing House speeds up translation and publication of *The Rabe Diary*
838	1997.05.14	The road of Tongyi translation software
839	1997.06.26	Chinese translations of all Hong Kong statute laws completed — Chinese and English legal texts now enjoy equal status
840	1997.06.28	No need to hire interpreters for court any more
841	1997.08.18	When will the "Goldbach conjecture" in ancient Chinese history be cracked?
842	1997.08.25	Xi'an Translation Training College meets social needs — Ten years of strict teaching produce tens of thousands of successful students
843	1997.09.04	After sixty years under seal, *The Rabe Diary* appears as testimony — Chinese translation published
844	1997.10.05	On my translation
845	1997.11.18	Multiple genres flourishing, many languages flying side by side — Fifth Minority Literature Creation Awards honour 60 writers and 3 translators from 24 ethnic groups; works by Lhoba, Jino and Tatar writers make the list for the first time
846	1997.11.24	Pocket-sized electronic translation device successfully developed
847	1997.11.27	Confucian classics know no borders — the Four Books translated into Hebrew
848	1997.12.10	A bridge to understanding American culture — Chinese translation of *The Random House College Dictionary* published
849	1997.12.13	*Selected Translations of Modern Classics in Literature and History* series
850	1997.12.16	Symposium on Wang Jinling's translation practice held in Beijing
851	1998.01.12	*Encyclopedia of Human Rights* translated and published
852	1998.02.21	The signatures to *The Communist Manifesto* and its first English translation
853	1998.03.05	Letting cultural classics speak in an engaging voice — on reading the "Wild Camel Translation Series"
854	1998.03.31	Internet translation device
855	1998.06.12	Another translation software package hits the market
856	1998.07.02	To awaken genuine self-respect — On the appearance of the Chinese translation of the English edition of *The Rape of Nanking*
857	1998.07.13	Tianjin "Tongyi" supports information-engineering projects
858	1998.07.26	Pitfalls in translated terms
859	1998.08.28	Translation in crisis — A second look at language problems in the European Union
860	1998.09.29	Princess Sirindhorn's translation *The Pen Blossoms* published
861	1998.10.13	Opening up the uncultivated field of private higher education to train versatile high-level talent — A report on Xi'an Translation Training College

862	1998.11.02	“Knowledge Economy Classics in Chinese Translation” series published
863	1998.11.07	Serving as interpreter for Mr. Yuanshan Zhengying
864	1998.11.09	Writings of Deng Xiaoping already translated into seven minority languages
865	1998.11.10	Emphasising listening, speaking, reading, writing and translation — College general English reforms show initial results
866	1998.12.04	International translation seminar for the 21st century held
867	1998.12.08	More than 500,000 people engaged in translation work in our country
868	1998.12.29	Chinese translation of the complete *Darwin’s Theory of Evolution* now available in full
869	1999.01.06	Writer and translator Ye Junjian passes away
870	1999.01.29	Review of the annotated modern-vernacular edition of *Gangjian Yizhi Lu*
871	1999.02.12	Renowned writer and translator Xiao Qian passes away
872	1999.03.09	Translated texts in short supply
873	1999.04.21	New verse translation of *Three Hundred Tang Poems* by Xu Fang published
874	1999.05.18	Chinese translation of *Illustrated Archaeological Records of the Western Regions* published
875	1999.06.15	*A Thousand Carefully Translated Poems and Songs from Past Dynasties*
876	1999.06.18	Some views on modern translations of ancient books
877	1999.06.18	Current state of modern translations of ancient books is worrying
878	1999.07.16	A master’s pen translating famous pieces — Review of *Collected Translations of Bing Xin*
879	1999.07.24	Translation software repositions itself
880	1999.09.07	Sparks of truth awaken the people — Preface to the “Torchbearers Translation Series”
881	1999.09.29	Chinese translation of the sixteenth edition of *Economics* published
882	1999.10.10	The first Japanese to translate *Selected Works of Mao Tse-tung*
883	1999.11.05	Chinese translation of Mrs. Rabin’s memoir published
884	2000.01.03	*Chinese translation of the Qur’an by Ma Qianyun* published
885	2000.01.07	Foreign visitors tour the Li River — Interpreters not needed
886	2000.02.13	Xiao Qian (1910–1999), outstanding writer, journalist and translator
887	2000.03.05	A deputy serving as interpreter for fellow deputies
888	2000.03.08	Mistranslated telegram and delayed delivery — Telecom and postal services cannot shirk responsibility
889	2000.03.25	Reflections on the fact that ninety percent of translated works fail to meet the mark
890	2000.04.08	Japanese edition of *Li Youcai’s Courtroom Stories*
891	2000.04.22	Pale, wooden “translationese”
892	2000.05.11	*Translation Studies*
893	2000.06.10	The bearing of a great translator — In memory of Ge Baoquan

894	2000.06.15	Jinxiang County: Farmers compete for foreign-language interpreter posts
895	2000.07.10	“Classic University Texts in Translation: Sociology” series published
896	2000.07.18	Zhiyang Net builds an online translation centre
897	2000.08.04	Translation work and talent training deserve greater attention
898	2000.08.06	Hope is nurtured here — A record of the development of Xi’an Translation College
899	2000.09.02	A new understanding of ancient world civilizations — Review of the “Foreign Archaeological and Cultural Classics in Translation” series
900	2000.09.05	“Xi’an Translation College Cup” competition for outstanding Olympic news reports to be held
901	2000.10.07	Introducing the “Leisure Studies Translation Series”
902	2000.10.14	“‘Xiyi Cup’ Olympic best-news competition opens for submissions”
903	2000.10.26	Winners announced in the “Xiyi Cup” Olympic best-news competition
904	2000.11.30	Latest Chinese translation of WTO legal texts published
905	2000.12.06	Renowned poet and translator Bian Zhilin passes away
906	2000.12.09	Renewing Chinese translations of Shakespeare’s plays — On the Yilin edition of *The Complete Works of Shakespeare*
907	2000.12.18	Chinese joins the global family of languages for voice translation
908	2000.12.21	National press tennis invitational to open; *The Glory of the Motherland, the Pride of the People* published; “Bid for Olympics Translators’ Day” activities launched in Beijing
909	2001.01.13	National commendation conference for senior Chinese translators held in Beijing; Qian Qichen and others present certificates to fifty translators
910	2001.01.16	Bidirectional Chinese–English translation on web pages achieved — bilingual browser breaks through the online language barrier
911	2001.01.24	U.S. develops wearable translation device
912	2001.02.09	Breakthrough achieved in speech translation technology
913	2001.03.09	Simultaneous interpreting in minority languages
914	2001.03.31	Easily crossing online language barriers — major breakthrough in web translation research
915	2001.04.13	Case of infringement of publishing and copyright on works by Ba Jin and others concluded — 14 authors and translators and People’s Literature Publishing House win
916	2001.04.14	“Yilin Children’s Library” series offered to readers
917	2001.05.14	“Business Tong” mistranslation turns “school” into “arsenal”
918	2001.05.24	China and the European Union cooperate to train high-level translators and interpreters
919	2001.07.02	Passing the sacred flame in a mountain village — Chen Wangdao and the birth of the first Chinese translation of *The Communist Manifesto*
920	2001.07.05	Aligning the curriculum with market demand — Dalian Translation College carefully trains talent for society

921	2001.07.14	A new annotated edition for future readers — On *A Vernacular Parallel-Prose Translation of Wenxin Diaolong*
922	2001.07.21	Chinese translation of *The Net* published
923	2001.08.21	Germany unveils simultaneous spoken-language translation system
924	2001.09.05	Jinling Translation Agency grows by focusing on "three strengthenings"
925	2001.12.08	A window onto contemporary Western academic thought — Brief review of the *Contemporary Western Academic Classics in Translation* series
926	2002.01.17	First Chinese translations in the "American Law Library" series released
927	2002.02.06	China and the United States jointly develop high-performance compiler system
928	2002.03.06	Translation work in minority languages gets under way
929	2002.04.11	*The Lord of the Rings* repeatedly pirated — Yilin Press offers 100,000-yuan reward to trace the source
930	2002.04.21	Xiamen standardises foreign-language translations in public signage
931	2002.07.04	*Classics of Public Law in Translation* series published
932	2002.07.16	Training practically oriented talent — Lessons from the running of Xi'an Translation College
933	2002.07.27	Xiao Qian and his translations
934	2002.11.11	Yanbian cadres go into villages to translate and explain the 16th Party Congress report
935	2002.12.26	Renowned translator Jiang Lu passes away
936	2003.01.05	Symposium held on the new Chinese translation of *Logic*
937	2003.01.10	"Marxism Studies Translation Series"
938	2003.01.14	Blossoming students at the foot of Cuihua Mountain — A report on Xi'an Translation College
939	2003.03.14	"Close-at-hand" interpreters
940	2003.04.08	Literary translation and two metaphors
941	2003.04.08	Literature and translation
942	2003.06.01	New Chinese translations of Milan Kundera's works published
943	2003.07.10	Translator qualification certification moves toward standardisation
944	2003.08.07	How "hot graduates" are trained — Interview with Ding Zuyi, president of Xi'an Translation College
945	2003.09.05	Beijing Language and Culture University opens a Chinese–Japanese simultaneous interpreting classroom
946	2003.09.29	Over 350 experts and scholars spend ten years compiling and translating — *Complete Nobel Prize Lectures* published
947	2003.10.15	*Dream of the Red Chamber* has been translated into twenty-seven languages
948	2003.10.25	At the symposium marking the 50th anniversary of the Central Compilation and Translation Bureau, Liu Yunshan stresses strengthening basic Marxist theory research to better serve reform, opening-up and modernisation
949	2003.11.13	Cheng Fangwu and the new translation of *The Communist Manifesto*

950	2003.11.20	Opening up a new stage in compilation and translation work in the course of making Marxism Chinese
951	2003.11.21	Dubbed foreign films must not lose their individuality in voice-over
952	2003.12.28	Ten years of honing for a single sword — Reflections on reading *New Annotations and New Translation of Wenxin Diaolong*
953	2004.01.16	Chinese translation of *The Secrets of Scientific Discovery* published
954	2004.03.03	Case of dictionary piracy against Shanghai Translation Publishing House concluded
955	2004.03.10	The hard work of translation
956	2004.03.17	High reward offered for translating the “oriental heavenly script”
957	2004.03.21	Beholding its splendour — On the publication of new Chinese translations of Kant’s three Critiques on the 200th anniversary of his death
958	2004.04.04	Designated textbooks for the National Translation Professional Qualification Examination
959	2004.05.25	Over thirty years by lamplight to produce six million words of translation — Luo Shiwen, first to translate the entire *Compendium of Materia Medica* into English
960	2004.06.01	Telling Andersen’s fairy-tale life — Interview with translator Lin Hua
961	2004.08.22	I served as interpreter for Comrade Xiaoping
962	2004.08.26	The busy world of Chinese translation
963	2004.08.30	Complete Chinese translation of the epic *Jangar* published in Urumqi
964	2004.09.24	New progress in the compilation, translation and study of Marxist classics
965	2004.09.25	The pros and cons of retranslation and collaborative translation
966	2004.09.25	Leading figures discuss literary translation
967	2004.10.19	A layman talks about translation
968	2004.11.05	Remarkable achievements in our army’s military translation work
969	2004.11.06	During his visit to the exhibition on China’s translation achievements, Liu Yunshan calls for supporting and promoting China’s translation cause to serve economic construction and reform and opening-up
970	2004.11.09	China’s translation industry: large but not strong, with worrying error rates — overall quality low and shoddy work widespread; foreign-language translation mistakes “everywhere to be found”, chiefly due to shortage of high-level talents
971	2004.11.09	Translated publications should discard the vulgar and retain the refined
972	2004.11.12	Unable to endure loneliness, few successors and short-sighted publishing — the current state of literary translation urgently needs to change
973	2004.11.16	Translated literature calls out for criticism
974	2004.11.17	Complete modern translation of the Twenty-Four Histories presented to the National Library
975	2004.12.07	The embarrassment and grievances of translators
976	2004.12.12	Poetry and prose alike outstanding, translations forming a body of work —

		On reading *Collected Works of Li Jiye*
977	2004.12.21	Manila: Chinese translation appears at the airport (photo)
978	2004.12.21	The translator's profession is like acting
979	2005.01.06	Chinese translation of *The Piano Teacher*, representative work of the 2004 Nobel literature laureate, published
980	2005.01.11	Learning to decode falsehoods
981	2005.01.18	With no Excellence in Translation Award at the Lu Xun Literature Prize and no first prize in translation contests, in an era when everyone is learning foreign languages, China's literary translation field lacks successors
982	2005.01.20	Translation is a craft
983	2005.01.28	Promoting dialogue between Chinese and Western philosophy through high-quality translation — Summary of the symposium on translation issues in Sino–Western philosophical exchange
984	2005.02.04	Why is there a generation gap in literary translation?
985	2005.02.25	Guizhou scholars decipher four volumes of ancient Shui pictographic books
986	2005.04.11	Zhengzhou: deaf-mute citizens vote as sign-language interpreters work busily (photo)
987	2005.04.29	Many gaps remain in literary translation
988	2005.05.23	Translation publishing industry: worries behind the glitz
989	2005.06.09	From 1 September, translation quality will be judged by the new "standards"
990	2005.06.26	Strindberg, great and mysterious — Translator's postscript to the Chinese edition of *Collected Works of Strindberg*
991	2005.07.05	Ten years of Cao Xueqin's toil and five rounds of revision produced *Dream of the Red Chamber*; over 200 years later, scholar Li Zhi-Hua spent 27 years translating it into French — a tireless dream-seeker of *Redology*
992	2005.07.13	The Shui people's encyclopaedia and a living fossil for decoding Xia–Shang culture — Shui script: picking up the memories of civilisation
993	2005.07.22	Audio-visual exports urgently need to break through "bottlenecks" — serious problems in translation, copyright, marketing networks and market competition
994	2005.08.04	China wins the right to host the 18th World Translation Congress
995	2005.08.15	All are hourly workers, yet pay differs by hundreds of times — conference interpreters and wedding staff earn high wages while domestic service pay is low; Ningbo introduces guideline rates for hourly work
996	2005.09.01	*Library of Chinese Classics*: a grand collection of Chinese–English bilingual texts; authoritative translations of Chinese cultural classics s
997	2005.09.20	Burning the candle of life to translate and study Andersen's works — Lin Hua walking through the fairy-tale forest
998	2005.09.22	A key to open the door of European culture — On Yang Guangsheng's edited and translated *Greek Mythology*

999	2005.10.07	“Enterprise Theory Translation Series”
1000	2005.10.10	Building bridges of communication — Interview with Zhu Jingwen, UN Chinese interpreter
1001	2005.10.27	China’s book imports outnumber exports by about nine to one — more policy support to help publications “go global” with translation subsidies, export tax rebates and funding for overseas book fairs
1002	2005.11.30	Mobile phones and translation devices expected to merge into one
1003	2006.01.15	In Ningxia, 2,000 rural youths become “village interpreters”
1004	2006.02.28	100 masterpieces ‘going global’: contemporary literature ‘translated’ abroad
1005	2006.03.04	Translating Tibetan
1006	2006.03.26	Faithfully recreating the original style — introducing the new Chinese translation of *Lolita*
1007	2006.04.09	New fruit of China–Italy academic and cultural exchange — “Croce’s Classic Works on Historiography” translation series published
1008	2006.04.09	Mu Dan’s translated poems and prose systematically published for the first time
1009	2006.04.13	Reciting famous British poems on Shanghai’s metro as the city builds a “cultural subway” — English translations of Tang poetry also avidly read on the London Underground
1010	2006.06.16	Popularising the classics must also move with the times — brief review of the abridged illustrated edition of *Capital*
1011	2006.08.20	Reflections of an old translator
1012	2006.11.05	Publishing insiders discuss the “Yilin phenomenon”
1013	2006.11.05	Speech at the opening ceremony of the Beijing Summit of the Forum on China–Africa Cooperation (translation) (4 November 2006)
1014	2006.11.05	Speech at the opening ceremony of the Beijing Summit of the Forum on China–Africa Cooperation (translation) (4 November 2006) (continued)
1015	2006.11.05	New translation of *Zhenguan Zhengyao* published
1016	2006.11.13	China to introduce 100 contemporary literary masterpieces to the world: first batch to ‘travel’ to three countries
1017	2006.12.12	This dragon is not that dragon — experts propose a new English name for the Chinese dragon
1018	2006.12.22	*Deliberative Democracy Translation Series*
1019	2007.01.05	Translation service providers for the Beijing Olympic Games confirmed
1020	2007.01.07	Moving beyond “compilation-and-translation mode” — overview of the National Symposium on Strategies for the Study and Teaching of Foreign Fine Arts
1021	2007.01.14	Versions of translated literature
1022	2007.01.28	New Chinese translation of *Small World* published
1023	2007.02.01	Changing the “one-way street” of cultural exchange — one hundred

		contemporary literary masterpieces to “go global”, first batch to be translated and introduced to Russia
1024	2007.03.18	On “Magic Mountain fever” as seen through the Chinese translations
1025	2007.03.28	Russia’s two waves of translating and introducing Confucianism
1026	2007.04.07	Spreading truth, achievements that live on — in deep mourning for Comrade Mao Anqing, translator of Marxist–Leninist classics
1027	2007.04.09	TCM terminology to get standardised English names — over 5,700 English entries already completed
1028	2007.06.11	Chinese literature translated into Urdu in Pakistan for the first time
1029	2007.07.02	Chinese lyric adaptor of “Moscow Nights” visits Russia for the first time
1030	2007.07.06	Carefully selected translations of the *People’s Daily* articles help readers understand the real China — *Must Read: Interesting China* published
1031	2007.08.22	At the book fair that closed on the 21st, two special series drew attention — Shanghai books stride forward in “going global”, accounting for about one-fifth of China’s exported titles, but shortage of translators is the biggest problem
1032	2007.08.23	Over 30,000 volunteers worldwide contribute translations — time-honoured Chinese brands now have foreign names in seven countries
1033	2007.12.23	Chinese translation of *The World Without Us* published
1034	2008.01.01	More than 1,500 terms now have unified translations — TCM terminology gains an international standard
1035	2008.01.16	“Gobelieve” adopted as the English correspondant of Goubuli — how should time-honoured brands be given foreign names?
1036	2008.03.02	World Translation Congress to be held in Shanghai
1037	2008.04.10	Thirty-fifth anniversary of China Foreign Translation and Publishing Corporation
1038	2008.04.19	Renowned translator Sidney Shapiro writes in *China Daily*: a history that must not be forgotten
1039	2008.06.19	*A Reader on Chinese Culture* launched in Beijing — multilingual editions to appear before the Olympics
1040	2008.07.10	Spain eager for sports exchanges with China trains 30 Chinese student interpreters — at the Beijing Olympics, Spanish athletes will “feel just like at home”
1041	2008.07.28	China’s translation robots reach world-leading level
1042	2008.08.05	World Translation Congress opens in Shanghai, its first time in Asia in 55 years
1043	2008.09.11	Elderly Japanese man translates *Human Insect* despite illness
1044	2008.09.24	Taiwan authorities decide to replace “Tongyong pinyin” with “Hanyu pinyin” for Chinese romanisation — a timely and pragmatic move
1045	2008.11.17	Sign-language interpreters help deaf and mute people make phone calls
1046	2008.11.17	Commercial Press to publish a complete series of “Chinese Translations of

		Famous Works”
1047	2008.12.23	Thoughts prompted by Wu Renbao changing his interpreter six times
1048	2009.01.04	Fu Lei’s translation and manuscript papers all donated to the National Library
1049	2009.01.10	Lu Xun Literature Institute holds training seminar for translators of minority literature
1050	2009.03.30	Project to translate and publish Chinese cultural classics launched
1051	2009.04.02	Translation project for *The Cambridge Ancient History* launched
1052	2009.04.06	On the distinction between “simplified adaptation” and “modern translation”
1053	2009.07.18	Chronological complete works of Lu Xun’s writings and translations published
1054	2009.07.28	The Five Classics to be translated into eight foreign languages
1055	2009.08.04	Singapore develops online speech-translation system
1056	2009.09.08	*Yilin* translation journal turns thirty
1057	2009.09.25	Symposium held on the publication of the collectors’ edition of the “Chinese Translations of World Academic Classics” series
1058	2009.09.25	How to overcome the hurdle of translation for culture ‘going global’
1059	2009.10.13	Mongolia publishes “Chinese Classics Translation Series”
1060	2009.11.14	306 veteran translators honoured
1061	2009.11.17	Translation is a bridge — and can also be a barrier (New Discourse: How to help the world understand Chinese culture (III))
1062	2009.11.25	China’s translation community looks forward to a “post-master” era
1063	2009.11.25	He has almost “translated the whole of China” (profile)
1064	2009.11.30	Farewell ceremony in Beijing for renowned translator Yang Xianyi
1065	2009.11.30	Is the bridge of translation still sound? (Culture Watch: Who will introduce China to the world? (I))
1066	2009.12.01	Why has literary translation fallen to “zero first choice”? (Culture Watch: Who will introduce China to the world? (II))
1067	2009.12.07	Winners announced for the inaugural Fu Lei Translation and Publishing Award
1068	2009.12.18	*Public Philosophy* translation series published (project follow-up)
1069	2009.12.28	Some reflections on compiling and translating two essay collections
1070	2009.12.28	Report on the compilation and translation of two essay collections
1071	2010.04.16	Hospitals in Xining urgently need Tibetan interpreters (all-out earthquake relief for disaster victims)
1072	2010.04.19	Tibetan–Chinese volunteer interpreters man “family hotline” phones (front line)
1073	2010.08.04	Helping tourists, guiding queues, interpreting languages… — one “Little Cabbage” Expo volunteer serves hundreds of people a day
1074	2010.08.14	Mistranslation of Naoto Kan’s remarks sparks controversy

1075	2010.08.18	Xinjiang holds advanced training course for key minority-language translators
1076	2010.08.31	Liu Yong of Nanjing Railway Police goes to Shanghai twice in three months to support the Expo — "police interpreter" walks the platforms (Expo gallery)
1077	2010.09.03	Standard Chinese translations set for the moon for the first time
1078	2010.12.03	"Translate with your ideals in mind"
1079	2010.12.10	Central Compilation and Translation Bureau working on second Chinese edition of the *Complete Works of Marx and Engels* based on the historically collated edition
1080	2011.05.23	Chinese and foreign scholars gather in Beijing to discuss translation education
1081	2011.06.24	Two letters from Marx recently acquired by the Central Compilation and Translation Bureau
1082	2011.11.21	"Century of Chinese Translations of Famous Works" series receives national funding for the first time
1083	2012.06.20	China Translation Profession Congress convenes
1084	2012.07.08	Huang Shang's relay of translation (afterword and collection)
1085	2012.10.05	Translation tools can be used but not abused — translation must be up to the mark (On literary theory)
1086	2012.11.11	Simultaneous interpreting carries the spirit across
1087	2012.11.15	Machine translation still no match for humans (Science and Technology Panorama)
1088	2012.12.24	Dancing fingertips connect the silent world (Visiting familiar strangers — inside the new profession of sign-language interpreters)
1089	2013.03.16	192 minority-language translators serve the Two Sessions
1090	2013.05.23	Serving as a volunteer "interpreter" (Moving stories around us — Jumei Duoji, part 4)
1091	2013.07.25	Editing approach and distinctive features of the new editions of *Selected Works of Marx and Engels* and *Selected Works of Lenin*
1092	2013.11.16	Combining literature and linguistics to explore new paths for translating Chinese fiction overseas
1093	2014.01.07	From passively taking on dubbing jobs to proactively building a major audio–visual industry
1094	2014.01.10	From 15 July on, foreign-language translations in public services must follow standardised guidelines
1095	2014.01.27	National multi-language film and television translation and dubbing base inaugurated
1096	2014.04.08	Verse translation of *The Complete Works of Shakespeare* published
1097	2014.04.25	Why is "zero translation" so widespread? (Decoding)
1098	2014.06.06	Multilingual editions of *Excerpts from Xi Jinping's Discussions on*

		Realising the Chinese Dream of the Great Rejuvenation of the Chinese Nation translated and published
1099	2014.07.29	In the new media era, what is worth translating? (Literary and Art Watch — focusing on translation culture)
1100	2014.08.04	Chinese translator wins FIT award for the first time
1101	2014.08.05	Filling the gaps in translating into minor languages (Literary and Art Watch – Focusing on Translation Culture)
1102	2014.08.05	A lifetime of stubborn devotion to translation (Profile)
1103	2014.08.12	Understanding "misreading" in translation dialectically (Literary and Art Watch – Focusing on Translation Culture)
1104	2014.08.20	International symposium on sinologists' literary translation held in Beijing
1105	2014.08.24	Translators need to be 'believers'
1106	2014.09.17	Central Compilation and Translation Bureau holds exhibition on translations of Deng Xiaoping's works
1107	2014.11.02	Seeing Chinese literature through translations of Mo Yan's works
1108	2014.11.03	International conference of sinologist translators held
1109	2015.02.24	Chasing dreams in the boundless "sea of translation," youth forever
1110	2015.03.04	From UN translator to Armed Police soldier (Marching China – Wonderful Stories – Spring Festival at the Grassroots)
1111	2015.03.19	Translation: how to tell China's story well to the world (In-depth Focus)
1112	2015.04.22	Chinese-into-foreign translation now outweighs foreign-into-Chinese
1113	2015.04.23	Important documents of the Party's 18th Central Committee Fourth Plenum translated and published in multiple languages
1114	2015.05.07	Sixty-four years compiling and translating the classics — faith forges nobility (Scholar Profile)
1115	2015.05.11	The rise in Chinese-into-foreign translation is no surprise
1116	2015.05.25	Why do imported blockbusters repeatedly run into "translation-gate"?
1117	2015.06.27	Literary reciprocal translation for better understanding (Jointly Creating a New Future for China–Russia Media Development)
1118	2015.08.26	Twenty foreign writers, translators and publishers receive special honours
1119	2015.09.01	China–foreign publishing and translation roundtable held
1120	2015.10.28	Don't let the facts be lost in "translation" (Quick Commentary)
1121	2015.12.12	Inaugural "Reading China" literary translation awards presented in Moscow
1122	2016.01.11	Contemporary masterpieces go straight to the world; literary translation connects hearts
1123	2016.06.11	Mr. Jingru, who annotated and translated *Guwen Guanzhi*
1124	2016.06.12	Expert seminar on China–foreign film and TV translation and dubbing cooperation held
1125	2016.06.20	Preserving the first Chinese translation of *The Communist Manifesto* (Glorious Course)
1126	2016.09.19	Provincial-level "red-headed" government documents must translate

		official jargon into plain language
1127	2016.10.09	Complete Russian translation of *The Peony Pavilion* published
1128	2016.11.05	Launch ceremony for the “Chinese Classics in Arabic Translation” series held in Beijing
1129	2016.11.15	Sworn to defend with their lives the first Chinese translation of *The Communist Manifesto*
1130	2016.11.23	“Association of Arab Translators and Sinologists” founded in Cairo
1131	2016.11.27	Invisible translators (Five Continents Teahouse)
1132	2017.01.08	Translation is hard; translating poetry is even harder (Five Continents Teahouse)
1133	2017.01.23	Service desk installs software to help foreigners translate; petrol stations send ginger soup to warm the journey home (2017 Spring Festival Travel Stories, Part Six)
1134	2017.04.14	Signage can act as a localised “translation” (Everyone Talks – Pragmatically Advancing Garbage Sorting II)
1135	2017.05.07	Translators should be defenders of their authors
1136	2017.05.23	Acting as “translator” for judges in deciding cases (In-depth Reading)
1137	2017.06.26	National standard on English translation and writing in public services released (News Brief)
1138	2017.07.04	How can translation and publishing keep pace with the times? (Youth Culture Forum)
1139	2017.07.14	Growing together with my translations
1140	2017.08.01	China’s four great classical novels now all translated into Malay and published
1141	2017.08.09	Cultural translation and interpretation help promote Chinese culture going global (Big Names’ Columns)
1142	2017.08.09	Telling China’s story well abroad requires innovation in external translation (New Knowledge, New Insights)
1143	2017.09.19	China and Ukraine sign cooperation agreement on translation, dubbing and broadcasting of Chinese TV dramas and films
1144	2017.11.14	“How did I become Braudel’s Chinese translator?”
1145	2017.11.23	Culture “going global” calls for a quality-conscious approach to translation (In-depth Observation)
1146	2017.11.28	Jointly translating and publishing volume two of *Xi Jinping: The Governance of China*
1147	2018.02.28	Smart translation really is magical (On the Front Line of the 2018 Spring Festival Travel Rush)
1148	2018.03.08	Livestreams adopt “smart translation”; audio now has a “transcriber”
1149	2018.04.19	Filmmakers from 24 countries train in film and TV translation and dubbing
1150	2018.05.01	Geng N.: a kind elder and doughty warrior of the translation world (Books and People)

1151	2018.05.31	Passing on the tradition of translating Chinese literature from generation to generation
1152	2018.08.23	Training course on Sino-foreign literary publishing and translation cooperation opens
1153	2018.08.30	Chinese–Hungarian edition of *Xinhua Dictionary* translation and publication project launched
1154	2018.11.29	Initial translation of *Gesar* completed
1155	2018.11.30	Zhuang-language intelligent translation software released
1156	2018.12.06	2018 annual conference of the Translators Association of China held in Beijing (Mini Reading)
1157	2019.01.08	Complete translation of the *Twenty-Four Histories*
1158	2019.01.27	Czech sinologist Kral's research and translations deeply shape perceptions of Chinese culture — an honour well deserved
1159	2019.02.15	Translating Chinese classics and promoting cultural exchange, Mongolian Confucius Institute dean wins many awards: "China's reform and opening-up gave me this opportunity"
1160	2019.02.22	Translation is about pursuing a "win–win" between two languages (Master Craftsmen Talk About Their Art)
1161	2019.02.22	Professor Zhong Jikun of Peking University, eminent translator — over sixty years building a cultural bridge between China and the Arab world (On Scholarship)
1162	2019.02.26	Complete translation of the *Twenty-Four Histories*
1163	2019.03.26	Nonagenarian German-language translator and Lifetime Achievement Award winner Song Shusheng — a lifetime of steadfast dedication, all the more weighty (Narration – A Lifetime Devoted to One Thing)
1164	2019.05.14	"Chinese Translations of Famous Works": new era, new mission
1165	2019.05.21	European sinology: exploring the historical and cultural factors behind China's development (Global View)
1166	2019.05.22	Reciprocal translation of books: we know each other better (Telling Asia's Story Well)
1167	2019.06.21	Translation and research enhance each other (Masters Talk About Their Craft)
1168	2019.07.02	With the translator's pen, building bridges to the world (Masters Talk About Their Craft)
1169	2019.07.14	On translations of the *Analects*
1170	2019.08.11	Good translation also needs "creation" (Five Continents Teahouse)
1171	2019.08.13	Lebanon's Digital Future Publishing has translated over 200 Chinese books in recent years — "Giving wings to the spread of outstanding Chinese titles"
1172	2019.08.20	Making literary translation and introduction more brilliant (Warm News, Hot Comment – Choose One Thing, Devote a Lifetime (14))
1173	2019.08.21	Thirteenth Special Book Award of China presented — 15 foreign writers,

		translators and publishers honoured
1174	2019.09.03	China–Russia classic and modern literature mutual translation project has translated and published 94 works
1175	2020.04.17	There are no shortcuts in translation (Master Craftsmen Talk About Their Art)
1176	2020.04.29	Volunteering with my skills, fighting the epidemic through "translation" (Warm News, Hot Comment)
1177	2020.06.14	The power of translation (Five Continents Teahouse)
1178	2020.06.21	Yara Misri, devoted to introducing contemporary Chinese literature — feeling the pulse of today's China up close (Overseas Guests Talk about China)
1179	2020.08.03	"The taste of truth is very sweet" (People's Forum) — marking the centenary of the first Chinese translation of *The Communist Manifesto* (I)
1180	2020.08.04	"Using the power of faith to create a better future" (People's Forum) — marking the centenary of the first Chinese translation of *The Communist Manifesto* (II)
1181	2020.08.05	"Only by remaining true to our original aspiration can we accomplish our mission" (People's Forum) — marking the centenary of the first Chinese translation of *The Communist Manifesto* (III)
1182	2020.08.06	"Our road will only grow broader and broader" (People's Forum) — marking the centenary of the first Chinese translation of *The Communist Manifesto* (IV)
1183	2020.08.07	"Making new and greater contributions to humanity" (People's Forum) — marking the centenary of the first Chinese translation of *The Communist Manifesto* (V)
1184	2020.08.09	"The mission of translation is communication and exchange, and serving the reader" (Overseas Guests Talk about China)
1185	2020.08.10	"Only the Communist Party of China can lead China" (People's Forum) — marking the centenary of the first Chinese translation of *The Communist Manifesto* (VI)
1186	2020.08.16	Greek translator Sotiris Chalikias — "Helping Greek readers understand the real China" (Overseas Guests Talk about China)
1187	2020.10.28	Three generations of compilers and translators firmly holding the front of Marxist–Leninist classics — letting truth travel through time and space (Narration – A Lifetime Devoted to One Thing)
1188	2020.11.04	Several reportages on poverty alleviation to be translated and published
1189	2020.12.20	First "Russian–Chinese Literary Diplomatic Translation Award" ceremony held in Beijing
1190	2021.01.06	China and Pakistan sign a memorandum on reciprocal translation and publication of classical works
1191	2021.01.19	China and South Korea sign a memorandum on reciprocal translation and

		publication of classical works
1192	2021.03.03	Publishing institutions from 17 countries sign memorandums with China to jointly translate and publish volume three of *Xi Jinping: The Governance of China*
1193	2021.03.17	China and Iran sign memorandum on mutual translation and publication of classic works — using books to promote civilisational exchange and mutual learning
1194	2021.04.06	Continuously strengthening capacity for Chinese-into-foreign translation (Opinion)
1195	2021.04.13	Deeply cultivating literary translation to enhance cultural exchange (Forum on Creative Transformation and Innovative Development)
1196	2021.04.27	China and Laos sign memorandum on mutual translation and publication of classic works, injecting cultural impetus into building a China–Laos community of shared future
1197	2021.05.10	Jointly safeguarding humanity's cultural diversity — interview with Egyptian sinologist and translator Faragani
1198	2021.08.22	"Chinese philosophy has moved me as never before" (Translator's Voice)
1199	2021.10.10	"Literary translation is more than a profession" (Translator's Voice)
1200	2021.10.20	It is a joy and an honour (Translator's Voice)
1201	2021.11.14	Perceiving Chinese culture through poetry (Translator's Voice)
1202	2021.11.21	A good translator must attend the "university of society" (Translator's Voice)
1203	2021.11.26	China and Armenia sign memorandum on the mutual translation and publication of classic works, opening a new stage of cultural exchange and mutual learning between the two countries
1204	2021.12.11	Foreign-language and minority-language translations of the main documents of the Sixth Plenary Session of the 19th CPC Central Committee published and distributed
1205	2022.01.11	First complete Dutch translation of *Dream of the Red Chamber* published
1206	2022.02.07	China and Kyrgyzstan sign memorandum on the mutual translation and publication of classic works, opening a new stage of cultural exchange and mutual learning between the two countries
1207	2022.02.10	Tibetan–Chinese *Gesar* translation series published and distributed
1208	2022.02.11	Building a rainbow to connect hearts (Translator & Book)
1209	2022.02.16	Domestic smart ski-wax trucks, intelligent robots and portable smart translation devices debut — "Made in China, Smartly" shines at the Beijing Winter Olympics (New Perspective)
1210	2022.02.17	Literary translation: between linguistic intuition and aesthetic beauty (Translator & Book)
1211	2022.03.20	Enabling readers to feel the vastness of culture (Translator & Book)
1212	2022.03.30	My translations of Maupassant (Translator & Book)

1213	2022.03.31	China and Azerbaijan sign memorandum on the mutual translation and publication of classic works, opening a new stage of cultural exchange and mutual learning between the two countries
1214	2022.04.06	A "honey-gatherer" in the garden of literature (Translator & Book)
1215	2022.05.13	Ever-flowing living waters (Translator & Book)
1216	2022.06.26	China and Mongolia sign memorandum on the mutual translation and publication of classic works, promoting cultural exchange and mutual learning between the two countries
1217	2022.07.05	Me and *Andersen's Fairy Tales* (Translator & Book)
1218	2022.07.08	Tianjin University of Technology "Jingyan Chuangyi" team develops real-time sign-language translation system — using artificial intelligence to understand sign language (Decoding – Exploring New Professions)
1219	2022.08.02	Deciphering ancient Egyptian hieroglyphs
1220	2022.08.16	Only friendship is the true treasure of the world (Translator & Book)
1221	2022.09.27	Renowned translator and children's writer Ren Rongrong — "I have spent my whole life writing books for children"
1222	2022.11.01	China and Yemen sign memorandum on the mutual translation and publication of classic works, promoting cultural exchange and mutual learning between the two countries
1223	2022.11.16	A bridge of literary translation spanning China and Thailand
1224	2023.01.06	"The world is like an ocean, the times like a strong wind" (Translator & Book) — On translating *Abai*
1225	2023.01.13	A conversation across time and space (Translator & Book)
1226	2023.02.10	Mutual literary translation builds a bridge for exchanges between Chinese and Arab civilisations
1227	2023.02.21	Drawing on Peking opera librettos to translate an epic masterpiece (Translator & Book)
1228	2023.03.15	China and Thailand sign memorandum on the mutual translation and publication of classic works, promoting cultural exchange and mutual learning between the two countries
1229	2023.03.28	A brief record of translating the works of Kenzaburo Oe (Translator & Book)
1230	2023.04.04	Annual conference of the Translators Association of China held in Beijing
1231	2023.04.24	Conveying love and light (Translator & Book) — Written on the 80th anniversary of the publication of the fairy tale *The Little Prince*
1232	2023.08.03	China and Jordan sign memorandum on the mutual translation and publication of classic works, promoting cultural exchange and mutual learning between the two countries
1233	2023.08.09	A poetic garden, a bridge of friendship (Translator & Book) — Impressions from translating Martin Walser
1234	2023.08.29	"Without entering the garden, how could one know how lovely spring is?"

		(Translator & Book) — Reflections inspired by the German translation of *On Gardens (Collector's Edition)*
1235	2023.09.06	Forum on the International Communication of Chinese Literature and Sixth International Symposium on Sinologists' Literary Translation opens in Nanjing
1236	2023.09.11	Academic forum on coordinated development of translation associations in Beijing–Tianjin–Hebei held
1237	2023.09.27	China and Nepal sign memorandum on the mutual translation and publication of classic works, opening a new stage of cultural exchange and mutual learning between the two countries
1238	2023.10.03	China and Saudi Arabia sign implementation plan for projects in literature, publishing and translation in Riyadh
1239	2023.11.27	High-level forum on innovation in external discourse in the new era and awards ceremony of the 35th Han Suyin International Translation Contest held in Beijing
1240	2023.12.19	China and Vietnam sign memorandum on the mutual translation and publication of classic works
